# A Modular Chemoenzymatic Cascade Simplifies Divergent Synthesis of Natural and Unnatural Benzylisoquinoline Alkaloids

**DOI:** 10.1002/advs.202513926

**Published:** 2025-10-20

**Authors:** Huiling Liu, Zhenbo Yuan, Yue Gao, Fei Li, Zhiwei Deng, Yan Zhang, Zhengshan Luo, Changmei Liu, Yijian Rao

**Affiliations:** ^1^ Key Laboratory of Carbohydrate Chemistry and Biotechnology Ministry of Education School of Biotechnology Jiangnan University Wuxi 214122 P. R. China; ^2^ Haixia Institute of Science and Technology School of Future Technology Fujian Agriculture and Forestry University Fuzhou Fujian 350002 P. R. China; ^3^ College of Bee Science and Biomedicine Fujian Agriculture and Forestry University Fuzhou 350002 P. R. China; ^4^ School of Life Sciences and Health Engineering Jiangnan University Wuxi 214122 P. R. China; ^5^ School of Pharmacy Nanjing University of Chinese Medicine Nanjing 210023 P. R. China

**Keywords:** (S)‐glaucine, artificial platform, benzylisoquinoline alkaloids, chemoenzymatic cascade, papaverine

## Abstract

Conventional chemical or biological synthetic methods often encounter challenges in conveniently preparing chiral natural and unnatural products with the same skeleton at a scalable level, due to their complexity with numerous chiral centers or uncharacterized enzymes. Herein, a simplified modular chemoenzymatic platform is designed featuring a streamlined four‐enzyme cascade coupled with a chemical module that involves one or two chemical steps. This platform enables the divergent synthesis of structurally diverse natural and unnatural benzylisoquinoline alkaloids (BIAs), including 1‐benzylisoquinoline, protoberberine, morphinan, and aporphine alkaloids. Moreover, the enantioselectivity of the chiral BIAs is up to 99% *ee*. The titer of papaverine reaches an impressive 2.83 g L^−1^, demonstrating substantial industrial potential. Overall, this work establishes a paradigm for constructing modular chemoenzymatic platforms that simplify the production of structurally diverse natural and unnatural products for drug development.

## Graphical abstract

A simplified chemoenzymatic platform is designed to integrate an enzyme cascade with subsequent chemical steps, enabling the divergent production of diverse benzylisoquinoline alkaloids (BIAs). Demonstrating remarkable versatility and enantioselectivity, four distinct classes of BIAs are efficiently generated, in which the natural medicine papaverine is produced in the titer of 2.83 g L^−1^.

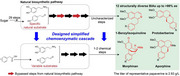

## 1 Introduction

**Figure 1:**
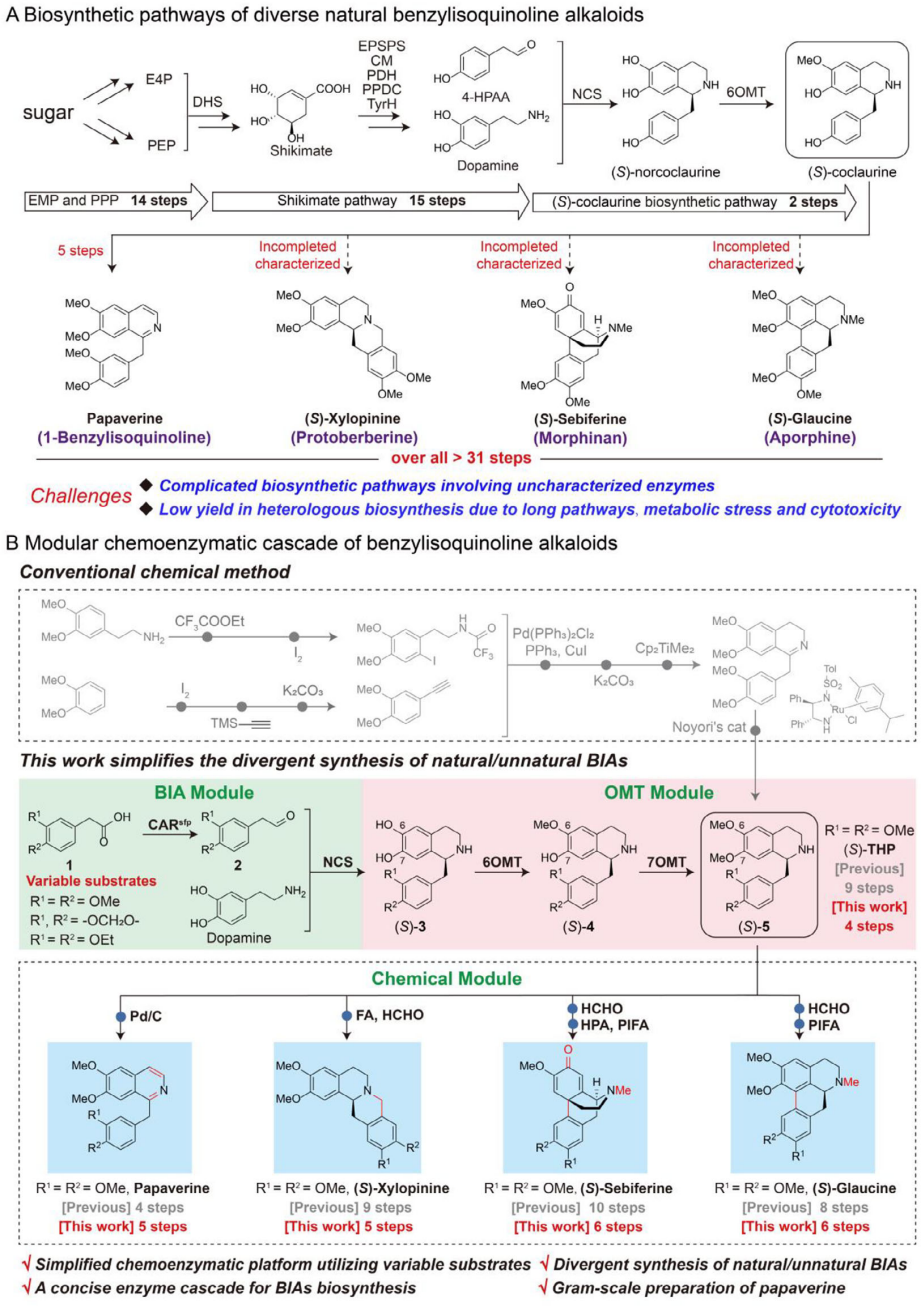
Design of a modular chemoenzymatic synthetic platform for divergent benzylisoquinoline alkaloids. A) Proposed biosynthetic pathways for divergent benzylisoquinoline alkaloids. PPP, pentose phosphate pathway; EMP, Embden‐Meyerhof‐Parnas pathway (glycolysis pathway); E4P, erythrose‐4‐phosphate; PEP, phosphoenolpyruvate; DHS, 3‐deoxy‐D‐arabino‐heptulosonate‐7‐phosphate synthase; EPSPS, 5‐enolpyruvylshikimate‐3‐phosphate synthase; CM, chorismate mutase; PDH, prephenate dehydrogenase; PPDC, phenylpyruvate decarboxylase; TyrH, tyrosine hydroxylase; 4‐HPAA, 4‐hydroxyphenylacetaldehyde; NCS, norcoclaurine synthase; 6OMT, 6‐*O*‐methyltransferase. B) A designed artificial chemoenzymatic cascade of various benzylisoquinoline alkaloids. The number of the reaction steps of chemical methods is colored in gray, while the number of the reaction steps of the designed cascade is colored in red. The chemical steps are highlighted by blue dots in the chemical module. CAR, carboxylic acid reductase; Sfp, phosphopantetheine transferase; 7OMT, 7‐*O*‐methyltransferase; (*S*)‐THP, (*S*)‐tetrahydropapaverine; FA, formic acid; HPA, phosphotungstic acid; PIFA, [bis(trifluoroacetoxy)iodo] benzene.

Plant‐derived alkaloids, such as benzylisoquinoline alkaloids (BIAs), are one of the most important types of natural products with a diverse range of physiological activities (**Figure** **1**).^[^ ^1^, ^2^, ^3^, ^4^, ^5^, ^6^, ^7^ ^]^ Notable BIA examples include papaverine from *Papaver somniferum* with antispasmodic, anticancer, antiviral, and anti‐inflammatory properties,^[^ ^1^, ^8^, ^9^ ^]^ (*S*)‐sebiferine from *Croton flavens* and (*S*)‐xylopinine from *Stephania rotunda* exhibiting antiplasmodial activity,^[^ ^10^, ^11^ ^]^ and (*S*)‐glaucine from *Glaucium flavum* with anti‐inflammatory, analgesic, antitussive, and antiaging effects.^[^ ^12^, ^13^ ^]^ However, many of these compounds are still extracted through traditional methods, which are notably influenced by factors such as low content, climate variability, cultivation practices, and geographical constraints.^[^ ^14^, ^15^, ^16^ ^]^ To address these challenges, continuous efforts are being made to develop novel synthetic methodologies with industrial potential.^[^ ^17^, ^18^, ^19^, ^20^ ^]^ Chemical total synthesis is frequently considered the primary strategy to overcome these issues. However, enantioselective synthesis in industrial applications is often hampered by the complexity of natural products, which typically feature numerous chiral centers and highly reactive functional groups.^[^ ^19^, ^21^ ^]^ These complexities often necessitate the use of heavy metal catalysts, intricate chiral catalysts or ligands, as well as extensive protection and deprotection processes.^[^ ^19^, ^22^, ^23^ ^]^ With advances in synthetic biology and a growing understanding of biosynthetic pathways, heterologous biosynthesis in engineered microorganisms has emerged as a promising alternative for the sustainable production of natural products.^[^ ^24^, ^25^, ^26^, ^27^, ^28^, ^29^ ^]^ However, the broader application of this system for the divergent synthesis of natural products is often limited by our understanding of their biosynthetic pathways, which even can be complicated by the presence of uncharacterized enzymes and the substrate specificity of most enzymes (Figure 1A).^[^ ^4^, ^30^, ^31^, ^32^, ^33^, ^34^ ^]^ Additionally, the efficient synthesis in engineered cells is frequently hindered by the lengthy and complex biosynthetic pathways, the toxicity of intermediates and final products, metabolic stress, as well as the inefficient expression of plant‐derived enzymes.^[^ ^8^, ^34^, ^35^ ^]^ Only a limited number of plant‐derived natural products with industrial potential are produced by this method.^[^ ^4^, ^17^, ^36^ ^]^ Therefore, it is highly desired to develop novel methods to overcome inherent synthetic challenges, such as constructing chiral centers and synthesizing complex natural products with post‐modifications.

Modular chemoenzymatic cascade reactions, which synergize the benefits of chemical methods and biocatalysis, have been proposed as a powerful alternative strategy for synthesizing complex compounds,^[^ ^37^, ^38^, ^39^, ^40^, ^41^ ^]^ involving diterpenoids,^[^ ^42^, ^43^ ^]^ piperidine derivatives,^[^ ^44^ ^]^ and steroids.^[^ ^45^ ^]^ In principle, biocatalytic reactions enable the highly stereoselective and chemoselective biosynthesis of key intermediates, followed by diverse synthetic pathways to produce versatile natural products and their derivatives through various chemical transformations.^[^ ^37^, ^38^, ^44^, ^46^ ^]^ This method simplifies the synthetic process of complex natural products by reducing or eliminating the need for functional group protection and deprotection.^[^ ^47^ ^]^ Therefore, we envision that this strategy could be effectively applied to the efficient synthesis of various plant‐derived BIAs with industrial potential (Figure 1B). Typically, the chemical synthesis of BIAs such as 1‐benzylisoquinolines, protoberberines, morphinans, and aporphines often involves eight to ten steps and the use of costly chiral catalysts or auxiliaries, making these methods commercially unfeasible.^[^ ^22^, ^23^, ^48^, ^49^, ^50^ ^]^ Although attempts have been made to biosynthesize many BIAs in engineered microorganisms, these processes are often complicated, incorporating the Embden‐Meyerhof‐Parnas pathway (EMP), pentose phosphate pathway (PPP), and Shikimate pathway with 29 steps, along with uncharacterized modification steps and difficulties in heterologous expression of plant‐derived enzymes (Figure 1A).^[^ ^8^, ^15^, ^16^, ^31^, ^32^, ^51^ ^]^ As a result, achieving high production of various BIAs still remains a challenge. Thus, we propose designing a concise enzymatic cascade that synthesizes a common chiral intermediate (Figure 1B). Then, this intermediate serves as a starting point for the divergent synthesis of BIAs through only a few chemical steps (Figure 1B), which is difficult to achieve through the multienzyme cascade as previously reported.^[^ ^52^ ^]^ By bypassing the uncharacterized steps, challenges associated with heterologous biosynthesis in engineered microorganisms, this approach could streamline the production process and enhance the efficiency of BIA synthesis.

In this study, we present a proof of concept for a modular chemoenzymatic cascade that enables scalable access to a diverse range of natural and unnatural BIAs using simple and variable substrates in just five to six steps (Figure 1B). Following enzyme engineering to enhance activity toward unnatural substrates, our specifically designed four‐enzyme cascade enables the efficient synthesis of the common intermediate (*S*)‐tetrahydropapaverine ((*S*)‐THP) at a titer of 3.33 g L^−1^ with 98% *ee*. This allows for the high‐yield synthesis of papaverine through a straightforward chemical step, achieving the highest titer of 2.83 g L^−1^ for biological processes.^[^ ^8^ ^]^ Moreover, the concise chemoenzymatic cascade also simplifies the synthesis of three additional types of BIAs: (*S*)‐xylopinine, (*S*)‐sebiferine, and (*S*)‐glaucine. Each of them was produced with over 98% *ee* using only one or two simple chemical steps. Importantly, our platform also accommodates the efficient synthesis of various natural and unnatural BIAs derivatives, including ethyl‐substituted BIAs, which are difficult to access via multienzyme cascades or heterologous biosynthesis owing to the paucity of ethyltransferases and the substrate specificity of downstream enzymes. Therefore, this work not only introduces a novel approach for the divergent synthesis of structurally diverse natural and unnatural BIAs simultaneously but also establishes a foundation for the development of BIA‐based drugs.

## 2 Results and Discussion

### 2.1 Design of a Concise Biocatalytic Cascade to Simplify the Divergent Synthesis of BIAs

To establish a streamlined platform for the simultaneous synthesis of various natural BIAs, four structurally distinct BIAs, papaverine, (*S*)‐xylopinine, (*S*)‐sebiferine, and (*S*)‐glaucine, were selected for retrosynthetic analysis. It reveals that (*S*)‐THP could serve as a pivotal platform intermediate for the divergent synthesis of these BIAs (Figure S1, Supporting Information), requiring minimal downstream chemical modification steps (Figure 1B). Inspired by the biosynthetic pathway of (*S*)‐THP (Figure S2, Supporting Information),^[^ ^8^ ^]^ we thus designed a concise four‐enzyme route starting from commercially available 3,4‐dimethoxyphenylacetic acid (**1a**) to synthesize (*S*)‐THP (Figure 1B). This cascade will circumvent the challenging native steps in the biosynthesis of (*S*)‐THP in microorganisms, including a hydroxylation step of (*S*)‐coclaurine catalyzed by P450 NMCH and two methylation steps at the 3′‐OH and 4′‐OH positions of (*S*)‐3′‐OH‐coclaurine (Figure S2, Supporting Information),^[^ ^8^ ^]^ thereby shortening the biosynthetic pathway. To further simplify the synthesis of distinct BIAs, we then proposed a modular chemoenzymatic cascade that consists of three functional modules. The first BIA module is responsible for the construction of the BIA skeleton. Here, carboxylic acid reductase (CAR) converts the commercially available substrate **1a** and its derivatives to the corresponding aldehydes **2**, which then undergo NCS‐catalyzed Pictet‐Spengler condensation^[^ ^53^ ^]^ with dopamine to form (*S*)‐**3**, establishing the core BIA skeleton. In the OMT module, two *O*‐methyltransferases sequentially methylate the 6‐OH and 7‐OH groups of (*S*)‐**3** to produce (*S*)‐**4** and the common intermediate (*S*)‐**5**, respectively. Finally, the chemical module enables the divergent transformation of (*S*)‐**5** into a variety of natural and unnatural BIAs (Figure 1B).

**Figure 2:**
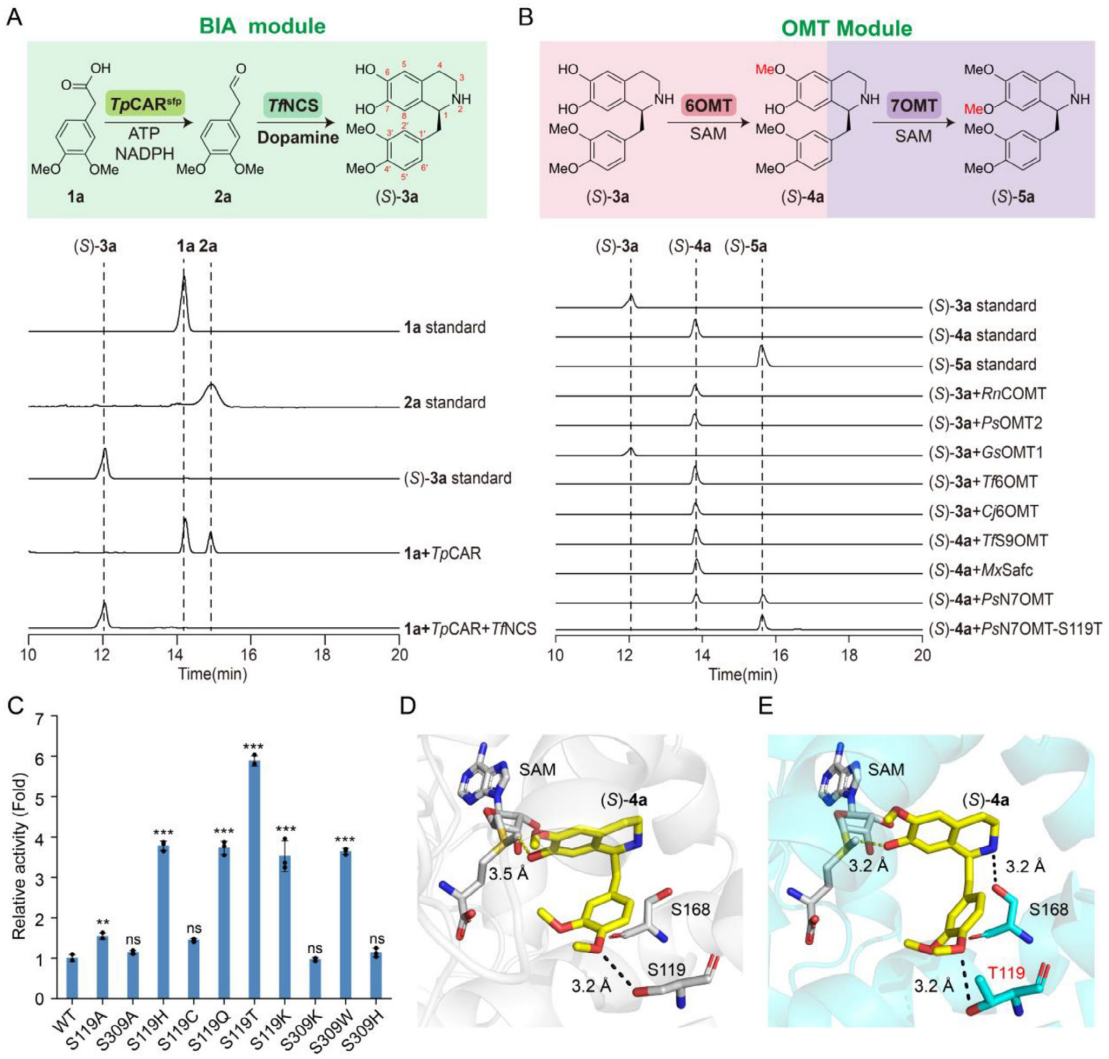
Establishment of a four‐enzyme cascade for the biosynthesis of (*S*)‐THP in vitro. A) Construction of the BIA module in vitro. The catalytic activity of *Tp*CAR and *Tf*NCS was analyzed by high‐performance liquid chromatography (HPLC). CAR activated by *Sfp* is named as CAR^Sfp^. SAM, *S*‐adenosylmethionine. B) Screening of 6‐*O*‐methyltransferases and 7‐*O*‐methyltransferases in vitro. The catalytic activity of 6OMT and 7OMT was analyzed by HPLC. C) Relative catalytic activity of mutants of *Ps*N7OMT. D) Structural model of wild‐type *Ps*N7OMT binding with (*S*)‐**4a**. (*S*)‐**4a** is colored in yellow. SAM, *Ps*N7OMT, and selected residues are colored in gray. E) Structural model of *Ps*N7OMT‐S119T binding with (*S*)‐**4a**. (*S*)‐**4a** is colored in yellow. SAM is colored in gray. *Ps*N7OMT‐S119T and selected residues are shown in cyan. Oxygen, nitrogen, and sulfur atoms are shown in red, blue, and yellow, respectively. The black dashed lines indicate hydrogen bonds. The yellow dash lines indicate the distance between SAM and 7‐OH of (*S*)‐**4a** in wild‐type *Ps*N7OMT and *Ps*N7OMT‐S119T, respectively. All data are presented as mean values of three independent experiments, and the error bars indicate ±sd. Two‐tailed Student's *t*‐test was conducted for analyzing the significant difference (*p* > 0.05, ns, no significance, ^*^ *p* < 0.05, ^**^ *p* < 0.01, ^***^ *p* < 0.001).

Then, the four‐enzyme cascade was first validated for the synthesis of (*S*)‐THP. At first, *Tp*CAR from *Tsukamurella paurometabola* ^[^ ^54^ ^]^ and *Tf*NCS from *Thalictrum flavum* ^[^ ^55^ ^]^ were expressed and purified for the analysis of the synthesis of (*S*)‐**3a** (Figure S3, Supporting Information). It showed that the substrate **1a** was able to convert to **2a** by *Tp*CAR, which could be further transformed to (*S*)‐**3a** by *Tf*NCS, confirming the successful construction of the BIA module (**Figure** **2**A). Next, five 6‐*O*‐methyltransferases, including *Rn*COMT from *Rattus norvegicus*,^[^ ^56^ ^]^ *Ps*OMT2 from *P. somniferum*,^[^ ^57^ ^]^ *Gs*OMT1 from *Gloriosa superba*,^[^ ^16^ ^]^ *Cj*6OMT from *Coptis japonica*,^[^ ^58^ ^]^ and *Tf*6OMT from *T. flavum*,^[^ ^59^ ^]^ were screened to the conversion of (*S*)‐**3a** to (*S*)‐**4a** (Figure 2B; Figure S3, Supporting Information). Except for *Gs*OMT1, the other four methyltransferases were able to catalyze the generation of (*S*)‐**4a**. In addition, the multifunctional methyltransferase *Gf*OMT1‐T178A/K146R/W22L/I258V/L117M/A291V from *Glaucium flavum* ^[^ ^60^ ^]^ was evaluated for its ability to convert (*S*)‐**3a** to (*S*)‐**5a** directly. However, a major peak corresponding to (*S*)‐**4a** and a very weak peak corresponding to (*S*)‐**5a** were observed, indicating the poor activity of this multifunctional methyltransferase to transform (*S*)‐**4a** to (*S*)‐**5a** (Figure S4, Supporting Information). Considering the enzymatic activity and protein expression level (Figure S5, Supporting Information), *Ps*OMT2 was selected for further investigation. Last, *Tf*S9OMT from *T. flavum*,^[^ ^61^ ^]^ *Mx*safc from *Myxococcus xanthus*,^[^ ^62^ ^]^ and *Ps*N7OMT from *P. somniferum* ^[^ ^63^ ^]^ were selected to evaluate their catalytic activity for converting (*S*)‐**4a** to (*S*)‐**5a** (Figure S3, Supporting Information). Among them, only *Ps*N7OMT could transform (*S*)‐**4a** to (*S*)‐THP ((*S*)‐**5a**). However, the incomplete conversion of (*S*)‐**4a** indicates that its catalytic efficiency needs significant improvement (Figure 2B). Nevertheless, the four‐enzyme cascade was successfully constructed for the biosynthesis of the common chiral intermediate (*S*)‐**5a**.

### 2.2 Protein Engineering of *Ps*N7OMT to Improve Its Catalytic Activity

To enhance the catalytic activity of *Ps*N7OMT for the conversion of (*S*)‐**4a** to (*S*)‐**5a**, (*S*)‐**4a** was first docked into the structure model of *Ps*N7OMT built by AlphaFold 3.0.^[^ ^64^ ^]^ Then, alanine scanning was conducted on residues within a 4 Å radius of (*S*)‐**4a**, including I123, S119, S168, M172, D175, N260, H263, S309, M292, D312, M313, and N316 (Figure S6, Supporting Information). The results showed that the catalytic activity of mutants *Ps*N7OMT‐S119A and *Ps*N7OMT‐S309A increased by 1.54‐fold and 1.14‐fold, respectively (Figure 2C; Figure S7, Supporting Information). Then, saturation mutagenesis at position S119 was performed, revealing that the catalytic activity of the variants S119H, S119C, S119Q, S119T, and S119K increased by 3.78‐fold, 1.45‐fold, 3.72‐fold, 5.88‐fold, and 3.52‐fold (Figure 2C; Figure S7, Supporting Information), respectively. For S309, due to the observed large cavity between S309 and (*S*)‐**4a**, it was mutated to lysine, histidine, and tryptophan to increase the steric hindrance. The results showed that the activities of mutants S309W and S309H increased by 3.64‐fold and 1.14‐fold (Figure 2C), respectively. However, the mutants exhibited dramatically decreased activity when they combined with S119 mutants (Figure S7, Supporting Information). The likely reason is that the spatial orientation shift of (*S*)‐**4a** in combinational mutant facilitates the formation of an interaction between the 7‐OH group of (*S*)‐**4a** and S168, as well as alters the distance between the 7‐OH group of (*S*)‐**4a** and SAM, leading to the inactivation of the mutant (Figure S8, Supporting Information). Thus, *Ps*N7OMT‐S119T with the best catalytic activity was chosen for further investigation. Molecular docking analysis suggested that the enhanced catalytic activity was due to the formation of a novel hydrogen bond between (*S*)‐**4a** and S168 in *Ps*N7OMT‐S119T, along with a reduced distance between SAM and the 7‐OH group of (*S*)‐**4a**, decreasing from 3.5 to 3.2 Å (Figure 2D,E).

### 2.3 One‐Pot One‐Step Strategy for the Biosynthesis of the Common Intermediate (*S*)‐THP

**Figure 3:**
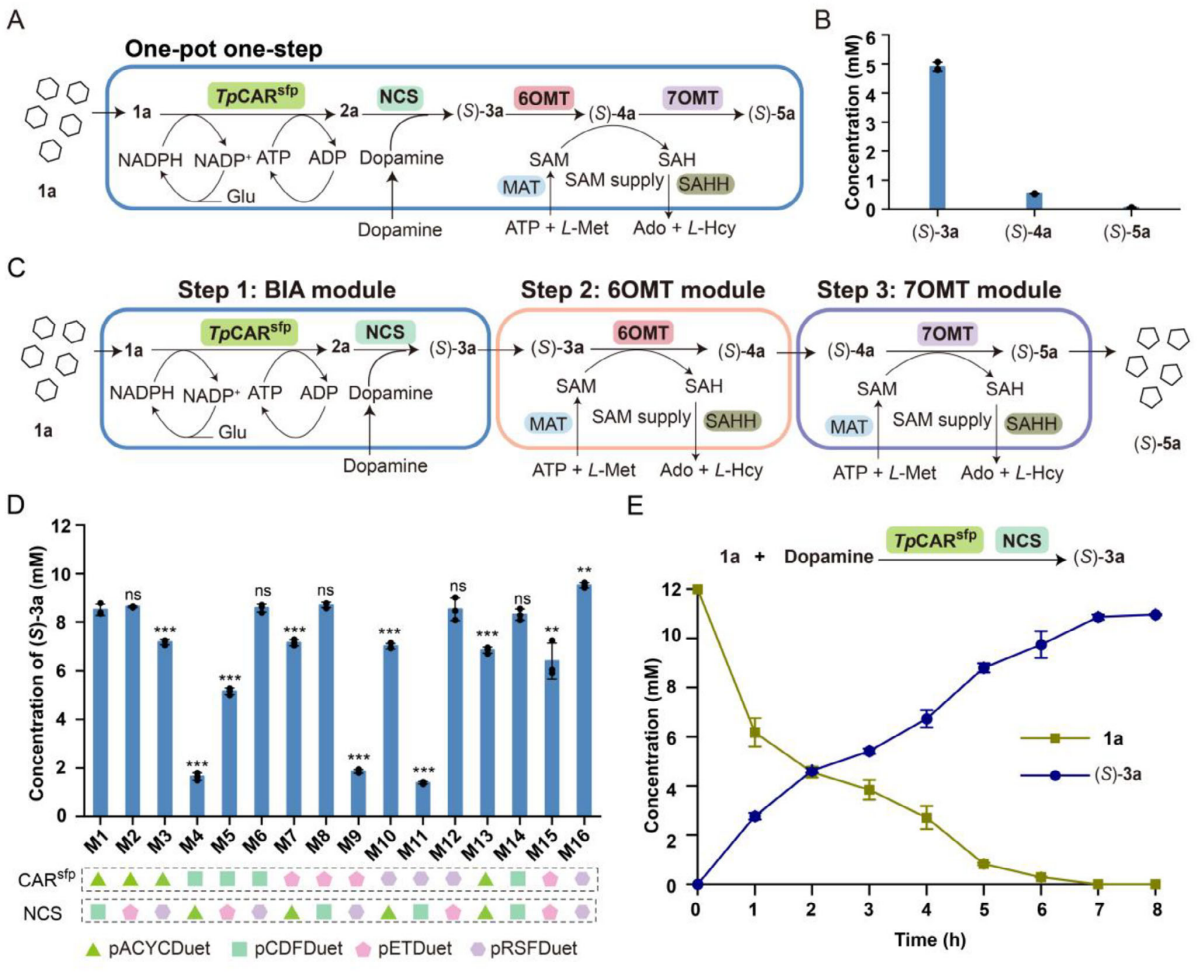
Modular biosynthesis of the intermediates (*S*)‐**3a**, (*S*)‐**4a**, and (*S*)‐**5a**. A) Illustration of the designed one‐pot one‐step whole‐cell catalysis for the preparation of (*S*)‐**5a**. The biosynthesis of (*S*)‐**5a** is catalyzed by CAR^sfp^, NCS, 6OMT, and 7OMT from **1a** and dopamine. *L*‐Met, *L*‐methionine; SAH, *S*‐adenosylhomocysteine; Ado, adenosine; *L*‐Hcy, *L*‐homocysteine; MAT, methionine adenosyltransferase; SAHH, *S*‐adenosylhomocysteine hydrolase. B) The titer of (*S*)‐**3a**, (*S*)‐**4a**, and (*S*)‐**5a** in one‐pot one‐step whole‐cell catalysis. C) Illustration of the designed one‐pot three‐step whole‐cell catalytic biosynthetic pathway for the preparation of (*S*)‐**5a**. The biosynthesis of (*S*)‐**5a** from **1a** and dopamine is catalyzed by CAR^sfp^ and NCS in the BIA module, 6OMT in the 6OMT module, and 7OMT in the 7OMT module. D) The titer of (*S*)‐**3a** in engineered strains M1‐M16. E) Time course of the biotransformation of **1a** and dopamine to (*S*)‐**3a** through the whole‐cell catalytic strain M16. All data are presented as mean values of three independent experiments, and the error bars indicate ±sd. Two‐tailed Student's *t*‐test was conducted for analyzing the significant difference (*p* > 0.05, ns, no significance, ^*^ *p* < 0.05, ^**^ *p* < 0.01, ^***^ *p* < 0.001).

To facilitate the large‐scale production of (*S*)‐**5a**, the designed four‐enzyme cascade was integrated into a whole‐cell catalytic system. This approach offers several advantages, such as the protection of enzymes from harsh reaction conditions, the elimination of the need for protein purification, and costly cofactors such as SAM and NADPH.^[^ ^14^, ^65^ ^]^ Initially, to inhibit the oxidation of aldehydes to alcohols, an engineered *E. coli* strain IAA with deletions of seven endogenous genes (*dkgB*, *yeaE*, *dkgA*, *yqhD*, *yahK*, *yjgB*, and *yqhC*) was constructed as described in previous studies (**Figure** **3**A).^[^ ^14^, ^66^ ^]^ Next, to increase the availability of SAM in *E. coli*, methionine adenosyltransferase (*Ec*MAT) from *E. coli* and *S*‐adenosylhomocysteine hydrolase (*Mm*SAHH) from *Mus musculus* were introduced into the strain IAA as the SAM regeneration system (Figure 3A).^[^ ^67^, ^68^, ^69^ ^]^ Last, to solve the low expression issue of *Ps*N7OMT‐S119T, various solubilizing tags, including SUMO, GST, and MBP, were fused to its *N*‐terminus, resulting in the increased protein expression level by 2.19‐fold, 1.94‐fold, and 1.77‐fold (Figure S9, Supporting Information), respectively. Based on their expression level, SUMO‐*Ps*N7OMT‐S119T (*Ps*N7OMT*) was selected for further studies. Then, the resulting strain BM1 with the co‐expression of *Tp*CAR, *Tf*NCS, *Ps*OMT2, *Ps*N7OMT*, *Ec*MAT, and *Mm*SAHH was applied to synthesize (*S*)‐**5a** through the one‐pot one‐step strategy. However, the yield of (*S*)‐**5a** was only 1% accompanied by substantial accumulation of (*S*)‐**3a** and (*S*)‐**4a** (Figure 3B). Sodium dodecyl sulfate polyacrylamide gel electrophoresis (SDS‐PAGE) analysis of strain BM1 indicated that all the enzymes were expressed at low levels to adversely influence the biosynthesis of (*S*)‐**5a**, probably due to the metabolic burden from excessive expression of exogenous proteins in a single cell (Figure S10, Supporting Information).^[^ ^14^ ^]^ This result indicated that a one‐pot multistep strategy should be developed to enhance the production of (*S*)‐**5a** (Figure 3C).

### 2.4 Modular Biosynthesis of (*S*)‐THP

According to the high accumulation of (*S*)‐**3a** shown in the one‐pot one‐step strategy, we first divided the four‐enzyme cascade into two modules, including the BIA module and the OMT module. For the BIA module, the engineered strain M1 containing *Tp*CAR, Sfp, and *Tf*NCS was constructed using two compatible pDuet vectors (Tables S1 and S2, Supporting Information). The effect of substrate concentration (dopamine and **1a**) on the titer of (*S*)‐**3a** was subsequently examined, revealing that a maximum concentration of 12 mm for each substrate resulted in a yield of 71% for (*S*)‐**3a** (Figure S11, Supporting Information). Next, a series of engineered strains featuring different plasmids was constructed, and reaction conditions were optimized to enhance the titer of (*S*)‐**3a**. The results showed that the engineered strain M16 (OD_600_ = 18) could produce 10.96 mm (*S*)‐**3a** (91% yield) from 12 mm dopamine and **1a** at pH 7.5 and 30 °C for 8 h (Figure 3D,E; Figure S12, Supporting Information). Notably, (*S*)‐**3a** predominantly accumulated in the reaction supernatant, which facilitates the synthesis of downstream products through centrifugation (Figure S12, Supporting Information). For the OMT module, the engineered strains containing *Ps*OMT2, *Ps*N7OMT*, *Mm*SAHH, and *Ec*MAT were used to test the capacity of converting (*S*)‐**3a** into (*S*)‐**5a**. Despite employing twelve engineered strains for the biosynthesis of (*S*)‐**5a** from (*S*)‐**3a**, only 2.88 mm of (*S*)‐**5a** was obtained in a conversion rate of 24% with the large accumulation of (*S*)‐**4a** (Figure S13, Supporting Information), suggesting the complex OMT module should be further divided into two separate steps.

**Figure 4:**
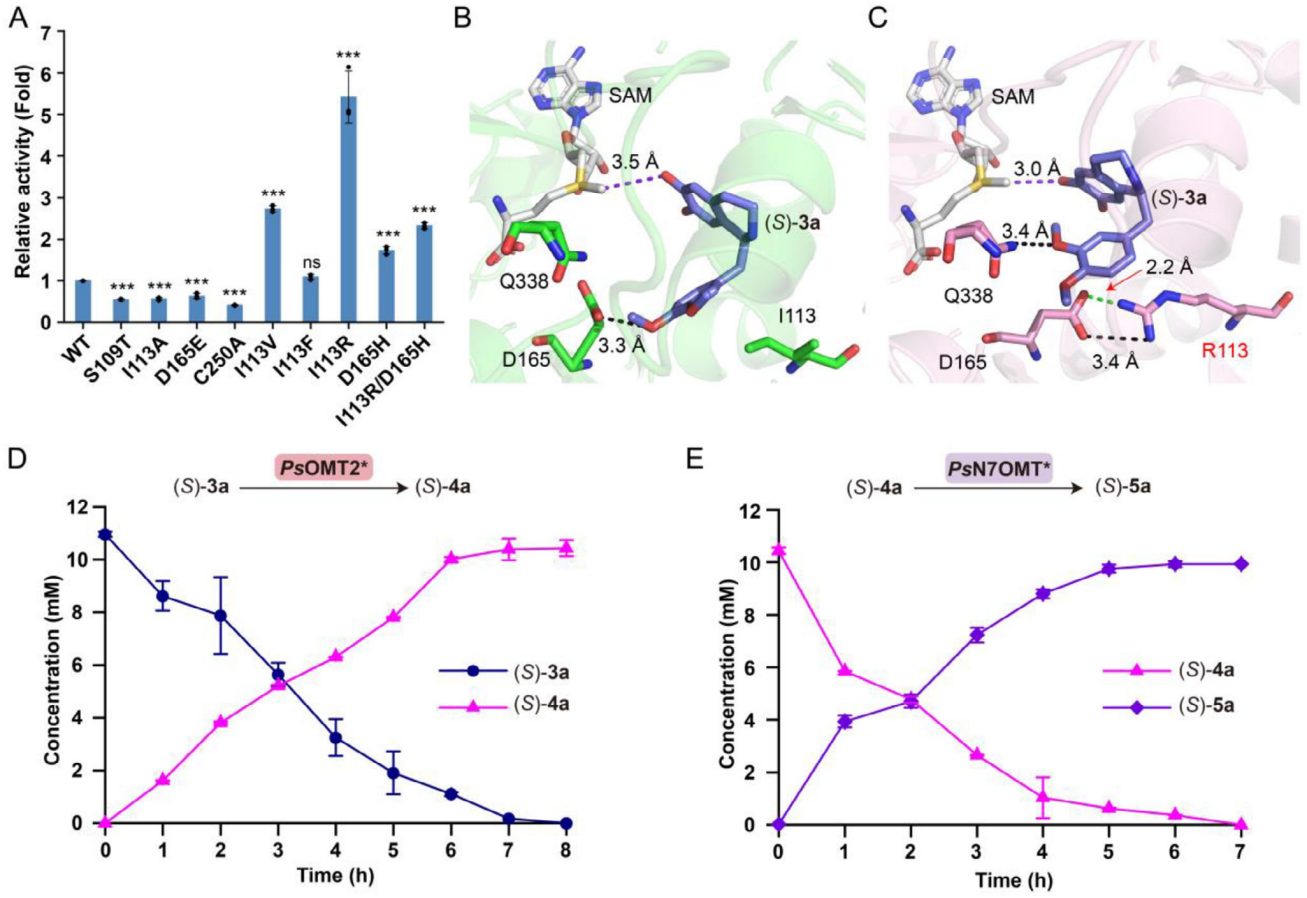
Rational design of *Ps*OMT2 for the efficient biosynthesis of (*S*)‐THP. A) Relative catalytic activity of mutants of *Ps*OMT2. B) Structural model of wild‐type *Ps*OMT2 binding with (*S*)‐**3a**. SAM is colored in gray. (*S*)‐**3a** is colored in purple. *Ps*OMT2 and selected residues in wild‐type *Ps*OMT2 are colored in green. C) Structural model of *Ps*OMT2‐I113R binding with (*S*)‐**3a**. SAM is colored in gray. (*S*)‐**3a** is colored in purple. *Ps*OMT2‐I113R and selected residues are colored in pink. Oxygen, nitrogen, and sulfur atoms are colored in red, blue, and yellow, respectively. The black dashed lines indicate hydrogen bonds, and the green dashed line indicates a salt bridge. The pink dashed lines indicate the distance between SAM and the 6‐OH group of (*S*)‐**3a** in wild‐type *Ps*OMT2 and *Ps*OMT2‐I113R, respectively. D) Time course of the biotransformation of (*S*)‐**3a** to (*S*)‐**4a** through the whole‐cell catalyst MA1. E) Time course of the biotransformation of (*S*)‐**4a** to (*S*)‐**5a** through the whole‐cell catalyst MB2. All data are presented as mean values of three independent experiments, and the error bars indicate ± sd. Two‐tailed Student's *t*‐test was conducted for analyzing the significant difference (*p* > 0.05, ns, no significance, ^*^ *p* < 0.05, ^**^ *p* < 0.01, ^***^ *p* < 0.001).

Then, the OMT module was further divided into the 6OMT and the 7OMT module for the synthesis of (*S*)‐**5a** (Figure 3C). For the 6OMT module, *Ps*OMT2, *Ec*MAT, and *Mm*SAHH were expressed in the engineered strain IAA (designated as MA0) (Table S1, Supporting Information). However, the yield of (*S*)‐**4a** was only 61%, with unreacted substrate remaining (Figure S14A, Supporting Information). This low yield likely resulted from insufficient enzymatic activity of *Ps*OMT2. To address this, structure‐guided engineering of *Ps*OMT2 was undertaken. Molecular docking analyses identified candidate residues interacting with (*S*)‐**3a** within a 4 Å radius via van der Waals force, including I113, F158, M162, L299, and M303, all of which were mutated to alanine (Figure S14B, Supporting Information). Additionally, amino acids S109, D165, D302, and Q338, which could potentially form hydrogen bonds with (*S*)‐**3a**, were substituted with residues of similar properties and steric hindrance to maintain hydrogen bonding. Despite these efforts, none of the mutations led to an increase in catalytic activity (**Figure** **4**A; Figure S14C, Supporting Information). However, based on the above results, it is suggested that I113 should be mutated to residues with larger steric hindrance, while D165 should be replaced with polar residues such as histidine, lysine, tyrosine, or arginine. Indeed, the mutants *Ps*OMT2‐I113V, *Ps*OMT2‐I113F, and *Ps*OMT2‐I113R exhibited increases in enzymatic activity of 2.73‐fold, 1.10‐fold, and 5.42‐fold, respectively, while the mutant *Ps*OMT2‐D165H resulted in a 1.73‐fold increase in activity. However, combinational mutants did not yield further enhancements (Figure 4A; Figure S14C, Supporting Information). Then, the mutant *Ps*OMT2‐I113R was selected. Molecular docking of *Ps*OMT2‐I113R with (*S*)‐**3a** revealed the presence of a 2.2 Å salt bridge and a 3.4 Å hydrogen bond between R113 and D165, as well as a new 3.4 Å hydrogen bond between the amino acid residue Q338 and the 3′‐OMe group of (*S*)‐**3a** (Figure 4B,C).

Notably, the reshaped substrate‐binding pocket reduced the distance between SAM and the 6‐OH group of (*S*)‐**3a** from 3.5 Å in *Ps*OMT2 to 3.0 Å in the mutant (Figure 4B,C; Figure S15 and Table S3, Supporting Information). This suggested that these newly formed interactions and increased spatial proximity contributed to the enhanced activity of the mutant *Ps*OMT2‐I113R. Next, the mutant *Ps*OMT2‐I113R (*Ps*OMT2*), along with *Ec*MAT and *Mm*SAHH, was introduced into the engineered strain IAA to construct four engineered strains with different plasmids (MA1‐MA4) (Figure S16A, Supporting Information). All strains successfully produced (*S*)‐**4a**, with strain MA1 yielding the highest titer (Figure S16B, Supporting Information). After optimizing pH, temperature, and the strain concentration, 10.43 mm (*S*)‐**4a** was obtained in 95% yield from (*S*)‐**3a** at pH 8.5 and 35 °C for 8 h using engineered strain MA1 (OD_600_ = 15) (Figure 4D; Figure S16, Supporting Information).

For the 7OMT module, four engineered strains with different plasmids (MB1‐MB4) expressing *Ps*N7OMT* with increasing expression level, along with an SAM supply system, were constructed for the biosynthesis of (*S*)‐**5a** from (*S*)‐**4a** (Figure S17A, Supporting Information). The strain MB2 showed the best activity (Figure S17B, Supporting Information). After optimizing pH, temperature, and strain concentration, 9.94 mm (a titer of 3.33 g L^−1^) (*S*)‐**5a** was obtained in 95% yield from (*S*)‐**4a** at pH 8.5 and 30 °C for 7 h by strain MB2 (OD_600_ = 12) (Figure 4E; Figure S17, Supporting Information). The overall production of (*S*)‐**5a** achieved 81% of the theoretical yield of **1a**, representing the highest reported titer for a biological process.

### 2.5 Scale‐Up Preparation of (*S*)‐THP and Its Unnatural Derivatives

**Figure 5:**
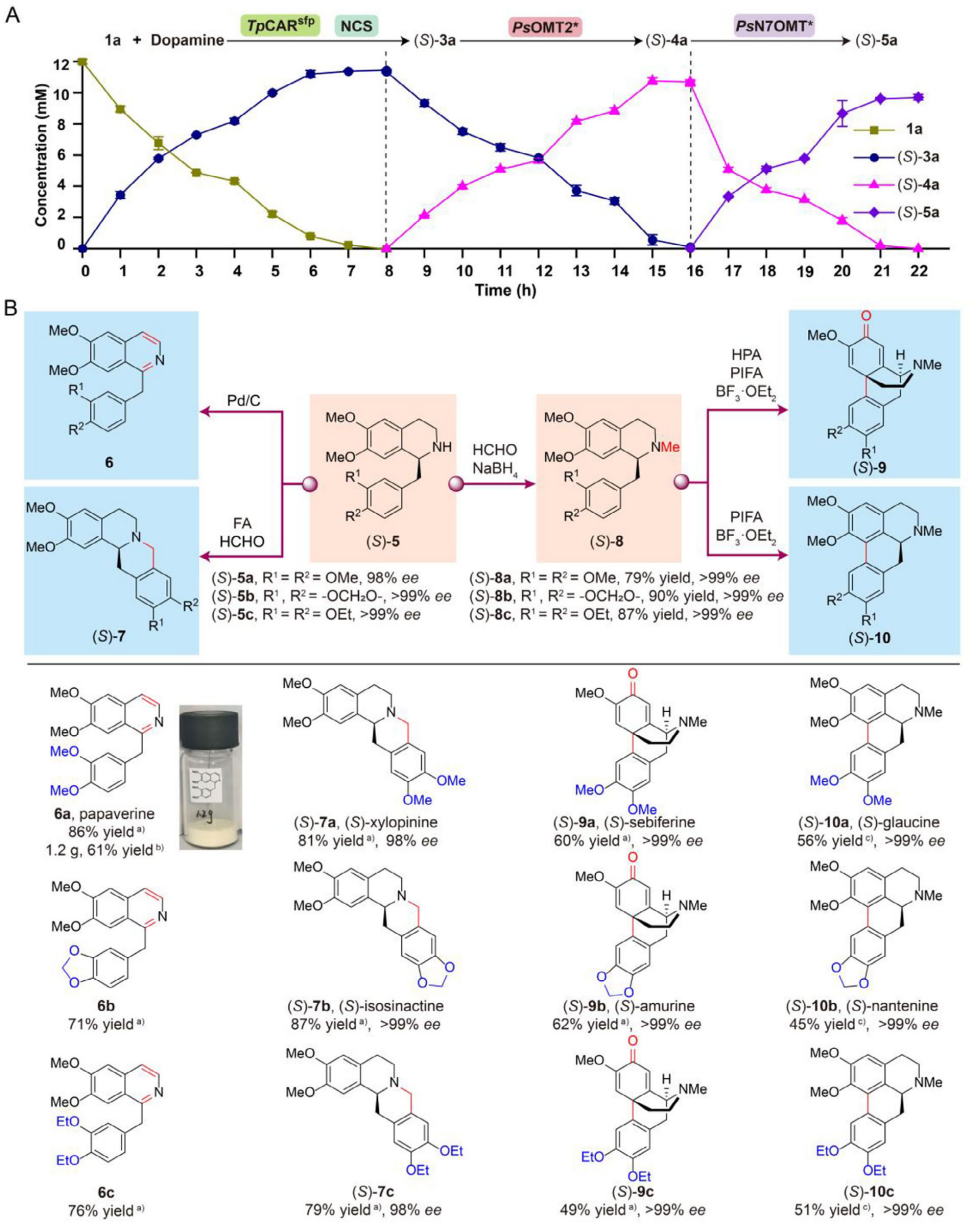
A modular chemoenzymatic platform for the divergent synthesis of natural and unnatural BIAs. A) Time course of the biotransformation from **1a** and dopamine to (*S*)‐**5a** in a 300 mL reaction system. All data are presented as mean values of three independent experiments, and the error bars indicate ± sd. B) The chemical module for the synthesis of natural and unnatural BIAs from (*S*)‐**5a** or its derivatives. ^a)^ Yields of **6a‐c** and (*S*)‐**7a‐c** are calculated based on the corresponding substrates (*S*)‐**5**, and yields of (*S*)‐**9a‐c** are calculated based on the corresponding substrates (*S*)‐**8** via gravimetric measurement. ^b)^ The gram‐scale preparation of papaverine. ^c)^ Yields of (*S*)‐**10a‐c** are calculated based on the corresponding substrates (*S*)‐**8** via HPLC.

With the optimization of the three modules completed, we investigated the feasibility of scale‐up preparation of (*S*)‐**5a** in a 300 mL reaction system using the optimized engineering strains M16, MA1, and MB2. As shown in **Figure** **5**A, a substantial titer of 3.33 g L^−1^ of (*S*)‐**5a** was achieved with an impressive yield of 81% and a 98% *ee* from 12 mm **1a** and dopamine. This yield was comparable to that obtained in the smaller reaction system (5 mL), demonstrating the excellent scalability of the designed multienzyme cascade. Importantly, the cascade was also effective for the synthesis of derivatives of (*S*)‐**5a**. (*S*)‐1‐(1,3‐benzodioxol‐5‐ylmethyl)‐1,2,3,4‐tetrahydro‐6,7‐dimethoxyisoquinoline ((*S*)‐**5b**) was produced with a titer of 3.05 g L^−1^, achieving 78% of the theoretical yield of **1b** and over 99% *ee* (Figures S18 and S19, Supporting Information). (*S*)‐1‐(3,4‐diethoxybenzyl)‐6,7‐dimethoxy‐1,2,3,4‐tetrahydroisoquinoline ((*S*)‐**5c**) was synthesized with a titer of 3.79 g L^−1^, resulting in an 85% of the theoretical yield of **1c** and over 99% *ee* (Figures S20 and S21, Supporting Information). These results demonstrate that the designed biocatalytic platform possesses significant potential for large‐scale production of (*S*)‐THP and its derivatives, offering sustainable methods for the synthesis of various natural and unnatural BIAs.

### 2.6 Divergent Synthesis of Natural and Unnatural BIAs

With the high production and stereoselectivity of (*S*)‐THP and its derivatives established, we then used them for the synthesis of four structurally diverse BIAs, including 1‐benzylisoquinoline, protoberberine, morphinan, and aporphine alkaloids, through one or two chemical steps.^[^ ^9^, ^22^, ^48^ ^]^ Initially, hydrogen peroxide was chosen for the oxidation of (*S*)‐THP to synthesize papaverine (**6a**), following methods described in previous studies.^[^ ^8^ ^]^ However, the resulting yield of papaverine was only 5%, accompanied by a significant amount of by‐products (Table S4 and Figure S22, Supporting Information). Then, commercial Pd/C was selected as the catalyst for this oxidation.^[^ ^9^ ^]^ After optimizing the solvents, catalyst loadings, and temperatures, the yield of papaverine was greatly improved to 86%, exhibiting better results than the method oxidized by hydrogen peroxide^[^ ^8^ ^]^ (Table S4, Supporting Information). Notably, the scale‐up preparation of papaverine was successfully achieved in 61% yield (1.2 g) (Figure 5B). The protoberberine (*S*)‐xylopinine ((*S*)‐**7a**) was also efficiently synthesized from (*S*)‐THP, yielding 81% and 98% *ee* in 37% formalin and formic acid solution at 90 °C for 2 h.^[^ ^22^, ^48^ ^]^ For the morphinan and aporphine alkaloids,^[^ ^22^ ^]^ subsequent transformations enabled the production of morphinan (*S*)‐sebiferine ((*S*)‐**9a**)^[^ ^70^ ^]^ and aporphine (*S*)‐glaucine ((*S*)‐**10a**)^[^ ^71^ ^]^ from (*S*)‐**8a**, which was derived from (*S*)‐**5a** through a simple chemical step. This process yielded 60% with >99% *ee* for (*S*)‐**9a** and 56% with >99% *ee* for (*S*)‐**10a** (Figure 5B). These synthetic pathways are notably shorter than conventional chemical methods, requiring only five to six steps to achieve the stereoselective synthesis of (*S*)‐xylopinine, (*S*)‐sebiferine, and (*S*)‐glaucine (Figure 1B). Additionally, these approaches avoid the use of costly metal catalysts, auxiliary reagents, and substrates, highlighting the efficiency and sustainability of the designed chemoenzymatic platform.

To further demonstrate the robustness and synthetic potential of this artificial platform, (*S*)‐THP derivatives (*S*)‐**5b** with a methylenedioxy bridge (>99% *ee*) and (*S*)‐**5c** with diethoxyl groups (>99% *ee*) prepared by the abovementioned multienzyme cascade, were utilized to synthesize the corresponding 1‐benzylisoquinolines **6b** and **6c**, protoberberines (*S*)‐isosinactine ((*S*)‐**7b**) and (*S*)‐**7c**, morphinans (*S*)‐amurine ((*S*)‐**9b**) and (*S*)**‐9c**, as well as aporphines (*S*)‐nantenine ((*S*)‐**10b**) and (*S*)**‐10c**, respectively. All of them were obtained in 45–87% yields (Figure 5B). Notably, this modular chemoenzymatic platform showed excellent enantioselectivity, generating (*S*)‐**7b‐**(*S*)‐**10b** and (*S*)‐**7c‐**(*S*)‐**10c** with up to >99% *ee*. Overall, these results demonstrate the successful establishment of a streamlined chemoenzymatic platform for the divergent synthesis of structurally diverse BIAs.

## 3 Conclusion

In this work, a simplified chemoenzymatic platform is designed for the divergent synthesis of both natural and unnatural 1‐benzylisoquinolines, protoberberines, morphinans, and aporphines. Through a combination of enzyme discovery, protein engineering, and metabolic engineering, we achieved the efficient and stereoselective biosynthesis of (*S*)‐THP and its derivatives as key intermediates from commercially available substrates via a concise biocatalytic cascade. This innovative approach enables the high production of various natural and unnatural BIAs through only one or two chemical steps. It effectively addresses the challenges associated with complicated enzymatic modifications often encountered with uncharacterized biosynthetic pathways or critical enzymes. Overall, this work highlights the potential of integrating enzymatic and chemical methodologies in drug development, paving the way for novel pharmaceutical discoveries.

## 4 Experimental Section

### Chemicals and Materials

Dopamine hydrochloride, 3,4‐dimethoxyphenylacetic acid, sodium ascorbate, and 3,4‐dimethoxyphenethylamine were purchased from Energy Chemical (Shanghai, China). ATP, *L*‐methionine, 3,4‐diethoxyphenylacetic acid, 2‐(benzo[d][1,3]dioxol‐5‐yl)acetic acid, phosphotungstic acid, hexafluoroisopropanol, and [bis(trifluoroacetoxy)iodo]benzene were purchased from Bide Pharmatech (Shanghai, China). Tryptone and Yeast extract were purchased from OXOID. Other chemical reagents were purchased from China National Pharmaceutical Group Corp (Shanghai, China) (Table S5, Supporting Information). Commercially available reagents were directly used without any further purification, unless otherwise noted.

The genes of *Tp*CAR (accession number: WP_013126039.1) from *T. paurometabola*, *Tf*NCS (accession number: ACO90248.1) from *T. flavum*, *Cj*6OMT (accession number: Q9LEL6.1) from *C. japonica*, *Rn*COMT (accession number: NP_036663.1) from *R. norvegicus*, *Gs*OMT1 (accession number: QLI49050.1) from *G. superba*, *Ps*OMT2 (accession number: NP_001413547.1) from *P. somniferum*, *Tf*6OMT (accession number: Q5C9L7.1) from *T. flavum*, *Tf*S9OMT from *T. flavum* (accession number: AY610512.1), *Mx*safc (accession number: 5LHM_A) from *M. xanthus*, *Ps*N7OMT (accession number: XP_026431254.1) from *P. somniferum*, and *Mm*SAHH (accession number: NP_001291457.1) from *M. musculus* were synthesized and codon‐optimized for *E. coli* BL21 (DE3) by Exsyn‐Bio (Wuxi, China). The gene of *Bs*Sfp was amplified from the genome of *Bacillus subtilis*. The gene of *Ec*MAT was amplified from the genome of *E. coli*. The primers used for gene amplification were purchased from Exsyn‐Bio (Wuxi, China), and all constructed plasmids were sequenced by Genewiz (Suzhou, China).

### Plasmids and Strains Construction

All plasmids and primers used in this study are listed in Tables S2 and S6 (Supporting Information), respectively. For plasmid construction, the fragments of target genes and vectors were amplified by PCR using 2 × Phanta UniFi Master Mix (Vazyme Biotech Co., Ltd., China) and purified using FastPure Gel DNA Extraction Mini Kit (Vazyme Biotech Co., Ltd., China). Then the gene fragments, vector fragments were assembled using 2 × MultiF Seamless Assembly Mix (ABclonal, Wuhan, China), transformed into *E. coli* TOP10, and screened by appropriate antibiotics on LB agar.

### Protein Expression and Purification

For the expression and purification of recombinant proteins, the strains were cultured in 2×YT medium, followed by the induction with 0.1 mm beta‐D‐1‐thiogalactopyranoside (IPTG) at 18 °C for 12 h. After centrifugation, the resuspended pellets in lysis buffer were lysed by the high‐pressure homogenizer, and centrifuged at 18300 rpm for 30 min at 4 °C. Subsequently, the target protein was purified by the NI‐NTA agarose column, desalted to remove imidazole, and stocked at −80 °C for further investigation.

### Enzymatic Activity Assay

For the BIA module, 200 µL reaction mixture containing MgCl_2_ (10 mm), ATP (4 mm), NADP^+^(1 mm), glucose (10 mm), sodium ascorbate (2 mm), substrate **1a** (2 mm), dopamine (2 mm), *Tp*CAR (5 µm), *Tf*NCS (5 µm), and GDH(5 µm) in 50 mm Tris‐HCl buffer (200 mm KCl, pH 7.5), was incubated at 30 °C for 6 h in 1.5 mL centrifuge tube. The reactions were terminated with acetonitrile. The resulting sample was filtered using 0.22 µm filters for HPLC analysis (Waters 2695) by a 2996 photodiode array (PDA) detector at 280 nm wavelength at 25 °C. The column was a C18 reverse‐phase column (4.6 × 250 mm, 5 µm), and the mobile phase contained solvent A (acetonitrile with 0.1% trifluoroacetic acid) and solvent B (water with 0.1% trifluoroacetic acid). A linear gradient elution method (0–1 min, 5% solvent A; 1–21 min, 47% solvent A; 21–21.5 min, 100% solvent A; 21.5–24.5 min 100% solvent A; 24.5–25 min 5% solvent A; 25–30 min 5% solvent A) was used for sample analysis.

For *Ps*OMT2 in the 6OMT module, a 200 µL reaction system consisting of (*S*)‐**3a** (2 mm), SAM (5 mm), MgCl_2_ (10 mm), and protein (5 µm) in 50 mm Tris‐HCl buffer (pH 8.0), was incubated at 35 °C for 30 min. The reactions were terminated with acetonitrile, and the resulting sample was analyzed as described above.

For *Ps*N7OMT in the 7OMT module, a 200 µL reaction system consisting of (*S*)‐**4a** (2 mm), SAM (5 mm), MgCl_2_ (10 mm), and protein (5 µm) in 50 mm Tris‐HCl buffer (pH 8.0), was incubated at 35 °C for 30 min. The reactions were terminated with acetonitrile, and the resulting sample was analyzed as described above.

### Molecular Docking

The proteins structures of *Ps*N7OMT and *Ps*OMT2 were predicted by Alphafold 3.0,^[^ ^64^ ^]^ and the *Ps*N7OMT/SAM and *Ps*OMT2/SAM binary complexes were formed by overlapping with a crystal complex of (*S*)‐norchloropurine 6‐methyltransferase (PDB ID: 5ICE). The substrates (*S*)‐**3a** and (*S*)‐**4a**, after energy minimization, were docked into the above binary complexes using Discovery Studio 2016 software, following the previous protocol.^[^ ^52^, ^72^, ^73^ ^]^

### Whole‐Cell Biocatalysis

For whole‐cell catalysis, the strains were cultured in 2×YT medium, followed by the induction with 0.1 mm IPTG. After 12 h, cells were harvested by centrifugation at 7000 rpm for 7 min. In the one‐pot one‐step method, a total of 5 mL reaction mixture containing substrate **1a** (6 mm) and dopamine (6 mm), MgCl_2_ (20 mm), glucose (20 mm), DMSO (5%), *L*‐methionine (50 mm), sodium ascorbate (15 mm), and ATP (30 mm) in 50 mm Tris‐HCl buffer (200 mm KCl, pH 7.5) was added with strain BM1 (OD_600_ = 21), which was incubated at 30 °C and 250 rpm for 12 h. The reactions were terminated with acetonitrile, and the resulting sample was analyzed as described above.

In the one‐pot three‐step method, a total of 5 mL reaction mixture containing substrate **1a** (12 mm) and dopamine (12 mm), MgCl_2_ (20 mm), glucose (20 mm), DMSO (5%), and sodium ascorbate (15 mm) in 50 mm Tris‐HCl (200 mm KCl, pH 7.5) was added with strain containing BIA module (OD_600_ = 18) in a 50 mL falcon tube, which was incubated at 30 °C for 8 h. The pH of the reaction supernatant obtained after centrifugation was adjusted to 8.5. Then, *L*‐methionine (50 mm) and ATP (15 mm) were added with the strain containing the 6OMT module (OD_600_ = 15) in the supernatant, which was incubated at 35 °C for 8 h. After the reaction of 6OMT, the pH of the reaction supernatant obtained after centrifugation was adjusted to 8.5. Then, *L*‐methionine (50 mm) and ATP (15 mm) were added with the strain containing the 7OMT module (OD_600_ = 12) in the supernatant, which was incubated at 30 °C for 7 h. Finally, the 100 µL reaction mixture was terminated with acetonitrile, and the resulting sample was analyzed as described above. All the above reactions were carried out in a 50 mL Falcon tube. For the scale‐up preparation of (*S*)‐THP and its derivatives, a total of 300 mL reaction mixture was incubated with strains in a 2 L shake flask using the above standard procedure.

### Isolation and Characterization of Intermediates and Products in the Biosynthetic Cascade

For the isolation of (*S*)‐**3a**, (*S*)‐**3b**, and (*S*)‐**3c**, the pH of the whole‐cell catalytic solution was adjusted to 8.5 with saturated Na_2_CO_3_ solution. Then, the mixture was extracted with dichloromethane, concentrated under reduced pressure, and purified by Waters 2775 Separations Module HPLC system. The column was an Agilent ZORBAX Eclipse XDB C18 column, 9.4 × 250 mm, 5 µm. The mobile phase consisted of solvent A (acetonitrile with 0.1% trifluoroacetic acid) and solvent B (water with 0.1% trifluoroacetic acid). An equal gradient semi‐preparative HPLC method (30% solvent A and 70% solvent B) was used for sample preparation. For the preparation of (*S*)‐**4‐c**, (*S*)‐**5‐c**, the pH of the whole‐cell catalytic solution was adjusted to 8.5 with saturated Na_2_CO_3_ solution. Then, the mixture was extracted with dichloromethane, concentrated under reduced pressure, and purified by silica gel column chromatography with petroleum ether: ethyl acetate to afford the desired compounds. The products (*S*)‐**3a‐c**, (*S*)‐**4a‐c**, (*S*)‐**5a‐c**, and (*S*)‐**10a‐c** were quantified by HPLC, and the corresponding standard curves are presented in Figure S23 (Supporting Information). Other products were quantified through gravimetric measurement.

To further characterize the intermediates and products, NMR (nuclear magnetic resonance) data were collected on Bruker Avance spectrometers (400 or 600 MHz for ^1^H NMR, and 101 or 151 MHz for ^13^C NMR), and HRMS (high resolution mass spectrum) data were collected on a Waters Maldi Synapt UPLC‐MS system. All enantioselectivities were analyzed via HPLC on a chiral stationary phase.

### Statistical Analysis

All data are presented as mean values of three independent experiments, and the error bars indicate ±sd. Two‐sided student's *t*‐test was utilized to assess differences between groups in Microsoft Excel, with statistical significance defined as *p* > 0.05, ns, no significance, ^*^ *p* < 0.05, ^**^ *p* < 0.01, ^***^ *p* < 0.001.

## Conflict of Interest

The authors declare no conflict of interest.

## Author Contributions

H.L. and Z.Y. contributed equally to this work. Y.R. supervised and designed the project. H.L., Z.Y., Y.G., F.L., Z.D., Y.Z., Z.L., and C.L. performed research and data analysis. H.L., Z.Y., and Y.R. wrote the paper. All authors read and approved the final manuscript.

## Supporting information



Supporting Information

## Data Availability

The data that support the findings of this study are available in the supplementary material of this article.
