# Supplementary material for: A Modular Chemoenzymatic Cascade Simplifies Divergent Synthesis of Natural and Unnatural Benzylisoquinoline Alkaloids
Source: Adv Sci (Weinh). 2025 Oct 20;13(1):e13926. doi: 10.1002/advs.202513926 (PMC12766994; doi:10.1002/advs.202513926)
Supplement: Supplementary file 1 — Supporting Information [file ADVS-13-e13926-s001.docx]

Supporting Information

A Modular Chemoenzymatic Cascade Simplifies Divergent Synthesis of Natural and Unnatural Benzylisoquinoline Alkaloids

**Authors**

Huiling Liu^†^, Zhenbo Yuan^†^, Yue Gao, Fei Li, Zhiwei Deng, Yan Zhang, Zhengshan Luo, Changmei Liu, and Yijian Rao*

Contents

Sequences of Nucleotides and Proteins Used in This Study 3

Characterization of Products 12

Supplementary Tables 36

Supplementary Figures 46

**Reference** 94

#

# Sequences of Nucleotides and Proteins Used in This Study

Codon-optimized nucleotide sequence of *Tf*NCS expressed in *E. coli* BL21 (*DE3*) [549 bp]

ATGCTGCATCACCAAGGGATTATCAACCAAGTCTCAACCGTCACTAAGGTAATCCACCACGAACTGGAAGTCGCTGCTAGCGCGGACGACATCTGGACCGTGTACTCCTGGCCGGGTCTGGCGAAACACTTACCGGACCTGCTTCCAGGGGCCTTCGAGAAACTTGAGATCATCGGAGACGGGGGGGTTGGGACAATCCTGGACATGACTTTCGTACCGGGGGAGTTTCCACACGAATATAAGGAAAAGTTCATTCTGGTTGACAACGAACATCGCTTAAAGAAAGTGCAAATGATTGAAGGAGGCTATCTTGACTTAGGTGTAACTTACTATATGGATACGATCCACGTTGTTCCAACTGGAAAAGACAGTTGTGTCATTAAGTCAAGTACTGAGTATCATGTCAAGCCGGAGTTCGTAAAGATTGTTGAGCCGCTTATTACAACTGGACCACTGGCCGCTATGGCCGATGCCATTTCGAAACTTGTTCTTGAACATAAGTCCAAGTCGAACTCCGACGAGATCGAAGCTGCGATCATCACTGTCTAA

Protein sequence of *Tf*NCS [182 aa]

MLHHQGIINQVSTVTKVIHHELEVAASADDIWTVYSWPGLAKHLPDLLPGAFEKLEIIGDGGVGTILDMTFVPGEFPHEYKEKFILVDNEHRLKKVQMIEGGYLDLGVTYYMDTIHVVPTGKDSCVIKSSTEYHVKPEFVKIVEPLITTGPLAAMADAISKLVLEHKSKSNSDEIEAAIITV

Codon-optimized nucleotide sequence of *Tp*CAR expressed in *E. coli* BL21 (*DE3*) [3318 bp]

ATGAGTATTGAGACCGTACAAAATGGCGTGCCCGCCGAGGGGTCTGTCCCCCCAGCCGACCAACAAACTGAACGTTTGCCCCAAGTTATTGCTCGCATTTTCGCTCAATTTGCGGACCGCCCCGCATTTGCCACGCGCGAGGCCGGTCCTGGTACCCCTTATGCGACCGTGTCCTATCGTGAGATTTGGCGTCGCGTCACGGCACTTGTAGCCTCTTGGCAATCTGAGGTAGCCCCAGGTGACTTCGTGGCGATCTTGGGATTCACATCTTCTGATTTTGTTACTGTCGATTTAGCTACTACACTTTTAGGAGCTCCAAACGTTCCACTGCAGGCTGGCGCACCAGCCGCGCGCATCGCAACCATCCTTGATGAAACCCGTCCAAAAATTTTGGCCGTTAGCGCCGATCAGGTCGATTTAGCCCAGGAAGCTTTAGCTGAATCAGCTGCCACGCCGCGTGTTGTCGTTTTCGATGGTGAGCGTGACGGCTATGAAGGGATCGAAGCCGATATTTTGTCCGGTAGCGCTTTACCGGCTCCGGAGTTTTTCGCGCCAGAGCCAGGGACTGATCCGCTTGTGACGTTAATCTACACTTCAGGTTCAACCGGCACGCCGAAGGGCGCCATGTACACAGAGCAGCTGGTACGTGACGCGTGGTTGAAAGTCGACTCGATTGTTGATATCGACATGCCCGCAGAGTCATTGCTTCATTTTCTGCCCATGTCGCACATGTATGGTCGCAATTGGCTTATCGCTGGCCTTGCGTCTGGAGGGACGGGATACTTCGCCGGGGCATCGGATATGAGTACCTTATTCGATGACCTGGCGGCTGCACGCCCCACCGCCATTGGCTTAGTGCCACGTGTTTGTGAATTAATCCACCAGCGCTACCTTGCGGTAGAGGCAGATACGGACGCGGAGACGGCTCGCGTAGAGTTGCGTGACCGCGTCCTGGGTGGACGTTTGCAAGCAGCTATGTGTGGAAGTGCTGCGCTGTCATCAGAGTTACAAACATTCATGGAATGGCTTTTAGGCATTGACATTCAAATTGGATACGGAAGTACCGAAGCGGGGGGTGTCATCCGCGACGGGGTAGTAGTCCGCCCACCGGTGACAGAGTATAAACTGATCGATGTCCCAGAATTAGGATATTTTGTCACTGACAGCCCTCATCCGCGCGGGGAGCTTTTGGTGAAATCGACACAGTTGATCCCCGGGTACTATAACAGCGACAAGCGCATCCGTGATGACGAAGGTTTTTATCGTACCGGCGACGTGATGGCTGAATTGGGACCAGACCGCTTGGAGTACGTCGATCGTCGTAGCAATGTGATCAAGTTGGCACAGGGTGAATTTGTACCGATTGCGCAGTTAGAAGCTATTTATGCGGCAGGACCCGATGTGCATCAGATCTTTCTTTACGGGACCAGCGAACGCAGCTATTTGATCGGGGTCGTGGTACCCGCGCCAGGCCCGGATGGAGAAACGGACGCGCAAACCCGTACTCGCGTGTTGGACGGACTGGCGGCTATTGCCCGCGAAAACGACTTGGCCGCTTATGAAGTTCCCCGTGACGTCTTGATTGAACGTGATCCCTTTTCGCAGGAGAACGGACTGCGTAGTGGAATCGGTAAGCTTGTACGCCCTGCACTTATCGCGCGCTACGGTGACCGTCTGCATGATTTATACGCACAAGCGGACACACGTCAACGCGAAGGCTTACGTGCCTTGGATGCGTCCGGACCAATCATCGACACTGTCTTAGGTGCGGCTGCCTTAACCTTAGGCGCTGACATCGCCGATTTTGATGCTGATACACGCTTCGGAGACTTGGGTGGGGATTCATTGTCTGCGCTTAGCTTAGCAACTACATTGGAAGGGCTGTACGACGTTCCAGTACCAGTACAAACGATCGTCGGGCCGACAGCAACTTTAGGAGGTGTAGCACGTCACATCGAGAAGGCTCGTTCAGGAGGCGTGGCTGCCCCCACCGCCGACAGTGTGCACGGGGTTGGAGCCAGCGTAGCACGTGCGACGGACTTGACATTGGAAAAGTTTATCGATCCAGAACTGCTTGCTTTAGCTCCAACATTGCCGGCCGCGACCGGTGAGCCAAATACTGTACTTTTAACAGGTTCGACGGGTTATCTTGGGCGCTTTCTGTTATTAGATTGGTTGCGTCGTGTGGCGCCACATGGGGGGACTGTTATTGCTTTGGTGCGTGGGGCGGATGCCGACGACGCACGCCGTCGTGTGACTGCGGCGATCGGAGATTCTGACCCCGACCTTACTCAAGAGTTCACGAGTCTGGCTGAGCATCACCTGCATGTGATTGCTGGCGACTTTGGGTCGCCGGCCTTAGGATTGGATGACGCGACCTGGTCGGATCTTGCAGGACGTGTAGATCATGTAGTACATTGTGGGGCTCTTGTTAATCATGTCCTGCCATACGACCAGTTATTTGGGCCAAACGTTGTCGCAACTGGTGAGGTTGTCCGTTTAGCGTTGACGACACGCCGCAAGTCAGTCGACTACGTGTCCACTGTCGCTGTCGTTCCTCAGGATGATGGGCGTGTCCTGGTTGAAGATGATGATGTACGTGAGTTAGGTGCGGAGCGCCGCATCGGCGCGGACGCTTATGCTAACGGCTACGCCGTGTCGAAATGGGCGGGGGAGGTTCTGTTGCATGAGGCAGCAGACTTAGCTGATCTGCCAGTACGCGTGTTCCGCTCCGATATGATTTTAGCGCACTCTCGTTTTCACGGTCAATTTAATGAAGTTGACCAATTTACCCGCCTGCTTCTTTCTATTGCGGAAACTGGGTTAGCTCCCGCCTCTTTCTACACACCTGACCCGAGCGGTCATCGTCCGCACTACGATGGTTTACCTGTTGACTTTACCGCTGAGGCAATTACAACTCTGTCAGCGGCAGGGCGTTCTGGATACCGCACCTTCCACGTTTTGAATGCGAATGATGATGGCGTGTCACTTGACAGCTTCGTCGATTGGATTGCGGCAAGTGGCCGTAGCATTGAACGTATCGACGACTATGATACTTGGTTTGCCCGCTTTGAGCAGGCATTACAACAATTACCCGACGAAGCGCGCCAGCGTAGCGTGTTACCTCTTTTGCACGCAGTGCGTGAGCCTGCGCCCGCGGCGGGAACGTCCGCTTTATCGGTGGATCGCTTCCGTGGTGCCGTACGTGAAACTGGTGTGGGTCCAGGAGACATCCCAGTTCTTGACCGTGCACTTATCGAGAAGTATTTGCGTGATTTCGAGACCGCGGGTTGGCTTGCCCCGGGTGCACGTGACTAA

Protein sequence of *Tp*CAR [1105 aa]

MSIETVQNGVPAEGSVPPADQQTERLPQVIARIFAQFADRPAFATREAGPGTPYATVSYREIWRRVTALVASWQSEVAPGDFVAILGFTSSDFVTVDLATTLLGAPNVPLQAGAPAARIATILDETRPKILAVSADQVDLAQEALAESAATPRVVVFDGERDGYEGIEADILSGSALPAPEFFAPEPGTDPLVTLIYTSGSTGTPKGAMYTEQLVRDAWLKVDSIVDIDMPAESLLHFLPMSHMYGRNWLIAGLASGGTGYFAGASDMSTLFDDLAAARPTAIGLVPRVCELIHQRYLAVEADTDAETARVELRDRVLGGRLQAAMCGSAALSSELQTFMEWLLGIDIQIGYGSTEAGGVIRDGVVVRPPVTEYKLIDVPELGYFVTDSPHPRGELLVKSTQLIPGYYNSDKRIRDDEGFYRTGDVMAELGPDRLEYVDRRSNVIKLAQGEFVPIAQLEAIYAAGPDVHQIFLYGTSERSYLIGVVVPAPGPDGETDAQTRTRVLDGLAAIARENDLAAYEVPRDVLIERDPFSQENGLRSGIGKLVRPALIARYGDRLHDLYAQADTRQREGLRALDASGPIIDTVLGAAALTLGADIADFDADTRFGDLGGDSLSALSLATTLEGLYDVPVPVQTIVGPTATLGGVARHIEKARSGGVAAPTADSVHGVGASVARATDLTLEKFIDPELLALAPTLPAATGEPNTVLLTGSTGYLGRFLLLDWLRRVAPHGGTVIALVRGADADDARRRVTAAIGDSDPDLTQEFTSLAEHHLHVIAGDFGSPALGLDDATWSDLAGRVDHVVHCGALVNHVLPYDQLFGPNVVATGEVVRLALTTRRKSVDYVSTVAVVPQDDGRVLVEDDDVRELGAERRIGADAYANGYAVSKWAGEVLLHEAADLADLPVRVFRSDMILAHSRFHGQFNEVDQFTRLLLSIAETGLAPASFYTPDPSGHRPHYDGLPVDFTAEAITTLSAAGRSGYRTFHVLNANDDGVSLDSFVDWIAASGRSIERIDDYDTWFARFEQALQQLPDEARQRSVLPLLHAVREPAPAAGTSALSVDRFRGAVRETGVGPGDIPVLDRALIEKYLRDFETAGWLAPGARD

Codon-optimized nucleotide sequence of *Bs*Sfp expressed in *E. coli* BL21 (*DE3*) [675 bp]

ATGAAGATTTACGGAATTTATATGGACCGCCCGCTTTCACAGGAAGAAAATGAACGGTTCATGTCTTTCATATCACCTGAAAAACGGGAGAAATGCCGGAGATTTTATCATAAAGAAGATGCTCACCGCACCCTGCTGGGAGATGTGCTCGTTCGCTCAGTCATAAGCAGGCAGTATCAGTTGGACAAATCCGATATCCGCTTTAGCACGCAGGAATACGGGAAGCCGTGCATCCCTGATCTTCCCGACGCTCATTTCAACATTTCTCACTCCGGACGCTGGGTCATTTGCGCGTTTGATTCACAGCCGATCGGCATAGATATCGAAAAAACGAAACCGATCAGCCTTGAGATCGCCAAGCGCTTCTTTTCAAAAACAGAGTACAGCGACCTTTTAGCAAAAGACAAGGACGAGCAGACAGACTATTTTTATCATCTATGGTCAATGAAAGAAAGCTTTATCAAACAGGAAGGCAAAGGCTTATCGCTTCCGCTTGATTCCTTTTCAGTGCGCCTGCACCAGGACGGACAAGTATCCATTGAGCTTCCGGACAGCCATTCCCCATGCTATATCAAAACGTATGAGGTCGATCCCGGCTACAAAATGGCTGTATGCGCCGTACACCCTGATTTCCCCGAGGATATCACAATGGTCTCGTACGAAGAGCTTTTATAA

Protein sequence of *Bs*Sfp [224 aa]

MKIYGIYMDRPLSQEENERFMSFISPEKREKCRRFYHKEDAHRTLLGDVLVRSVISRQYQLDKSDIRFSTQEYGKPCIPDLPDAHFNISHSGRWVICAFDSQPIGIDIEKTKPISLEIAKRFFSKTEYSDLLAKDKDEQTDYFYHLWSMKESFIKQEGKGLSLPLDSFSVRLHQDGQVSIELPDSHSPCYIKTYEVDPGYKMAVCAVHPDFPEDITMVSYEELL

Codon-optimized nucleotide sequence of *Ps*OMT2 expressed in *E. coli* BL21 (*DE3*) [1041 bp]

ATGGAAACCGTTTCTAAAATTGACCAACAAAATCAGGCCAAAATCTGGAAGCAGATTTACGGGTTTGCAGAGTCCTTGGTACTGAAATGCGCAGTACAACTGGAGATTGCAGAGACGTTGCATAACAATGTGAAACCTATGTCGTTAAGTGAGCTGGCATCGAAGCTTCCAGTGGCACAGCCGGTGAACGAGGACCGTTTGTTTCGTATTATGCGCTACTTAGTTCACATGGAGTTATTCAAAATCGACGCAACGACTCAGAAGTATTCTCTTGCCCCTCCTGCAAAGTACCTGCTGCGTGGATGGGAGAAATCTATGGTGGATTCTATTTTGTGCATCAATGACAAGGATTTCTTGGCCCCTTGGCACCACTTGGGTGACGGTCTTACTGGAAACTGCGACGCTTTCGAAAAAGCGTTGGGTAAATCCATTTGGGTCTACATGAGTGAAAATCCTGAGAAGAACCAATTGTTCAATGCGGCAATGGCCTGCGATACTCGTCTGGTGACAAGTGCGTTGGCTAATGAATGCAAATCCATCTTCTCAGACGGGATCTCAACTTTGGTTGATGTTGGTGGGGGGACAGGAACAGCAGTAAAAGCTATTTCAAAGGCCTTTCCAGACATCAAATGCACGATCTACGATTTACCCCATGTGATCGCGGATTCTCCCGAAATTCCGAATATCACGAAAATCTCAGGTGATATGTTTAAATCAATCCCCTCTGCAGATGCTATTTTCATGAAATGTATCTTGCATGATTGGAATGACGACGAATGCATTCAAATCTTGAAACGTTGTAAAGAAGCTCTGCCAAAAGTCGGCAAAGTTATCATCGTTGACGTAGTTATTGACATGGATTCGACACATCCATACGCGAAAATTCGTCTTACCCTTGACTTAGATATGATGTTGAACACTGGAGGGAAAGAACGTACTAAGGAGGAATGGAAGACCTTGTTCGACGCCGCGGGCTTCGCCTCCCATAAAGTTACGCAAATTAGCGCGGTGCAAAGTGTTATTGAAGCCTACCCGTACTAA

Protein sequence of *Ps*OMT2 [346 aa]

METVSKIDQQNQAKIWKQIYGFAESLVLKCAVQLEIAETLHNNVKPMSLSELASKLPVAQPVNEDRLFRIMRYLVHMELFKIDATTQKYSLAPPAKYLLRGWEKSMVDSILCINDKDFLAPWHHLGDGLTGNCDAFEKALGKSIWVYMSENPEKNQLFNAAMACDTRLVTSALANECKSIFSDGISTLVDVGGGTGTAVKAISKAFPDIKCTIYDLPHVIADSPEIPNITKISGDMFKSIPSADAIFMKCILHDWNDDECIQILKRCKEALPKVGKVIIVDVVIDMDSTHPYAKIRLTLDLDMMLNTGGKERTKEEWKTLFDAAGFASHKVTQISAVQSVIEAYPY

Codon-optimized nucleotide sequence of *Ps*N7OMT expressed in *E. coli* BL21 (*DE3*) [1074 bp]:

ATGGAGGTGGTTTCTCAGATCGACCAGGAGAACCAGGCAATCATTTGGAAGCAAATCTACGGATTCTCGGAATCGCTTTTGCTGAAATGTGCTGTCCAATGCGAAATTGCGGAGACAATTCATAACCATGGGACTCCTATGAGCATTCTGGAGCTGGCTGCCAAATTGCCTATTGATCAACCAGTAAATATCGACCGCTTGTATCGTGTAATGCGTTACTTAGTACATCAAAAATTATTCAATAAGGAAGTAATTTCCACACTGAACGGTGGGACCGTCCAAGTCACCGAAAAGTATTGGCTTGCCCCACCGGCCAAGTATTTAATTCGTGGCTCCCAACAGTCGATGGTACCCTCGGTATTGGGTATCATTGACGAGGATATGTTCGCTCCGTGGCATATTCTTAAAGATTCTTTGACGGGTGAATGTAATATTTTCGAAACCGCATTGGGCAAGTCAATTTCTGTCTACATGTCAGAGAATCCAGAGATGAATCAGATCAGTAATGGCGCGATGGCCTTCGATAGCGGACTGGTGACATCGCATCTGGTGAATGAATGTAAATCGGTTTTCGGAGATGAGATCAAGACACTGGTAGACGTTGGAGGTGGAACTGGCACGGCCTTGCGTGCGATTTCGAAGGCCTTTCCAAACATTAAATGTACGTTATTTGATTTACCACATGTAATCGCGGATTCACCTGAGATCCCCACGATCACGAAGGTGAGTGGAGATATGTTCAAGTCAATCCCTTCAGCGGATGCTATCTTTATGAAAAACATTCTTCACGATTGGAATGACGACGAATGTATCCAGATCTTGAAACGTTGTAAGGACGTAGTATCCGCGGGTGGTAAGTTAATCATGGTCGAAATGGTGTTAGATGAAGATTCATTCCACCCTTACTCCAAGCTGCGCTTGACGAGCGATATCGACATGATGGTTAACAATGGCGGAAAGGAACGCACGGAAAAGGAGTGGGAGAAGTTATTTGATGCTGCCGGTTTTGCGTCATGCAAATTTACACAAATGAGTGTCGGTTTCGCTGCACAGTCAATCATTGAAGTTTATTAA

Protein sequence of *Ps*N7OMT [357 aa]

MEVVSQIDQENQAIIWKQIYGFSESLLLKCAVQCEIAETIHNHGTPMSILELAAKLPIDQPVNIDRLYRVMRYLVHQKLFNKEVISTLNGGTVQVTEKYWLAPPAKYLIRGSQQSMVPSVLGIIDEDMFAPWHILKDSLTGECNIFETALGKSISVYMSENPEMNQISNGAMAFDSGLVTSHLVNECKSVFGDEIKTLVDVGGGTGTALRAISKAFPNIKCTLFDLPHVIADSPEIPTITKVSGDMFKSIPSADAIFMKNILHDWNDDECIQILKRCKDVVSAGGKLIMVEMVLDEDSFHPYSKLRLTSDIDMMVNNGGKERTEKEWEKLFDAAGFASCKFTQMSVGFAAQSIIEVY

Codon-optimized nucleotide sequence of *Mm*SAHH expressed in *E. coli* BL21 (*DE3*) [1299 bp]

ATGTCTGACAAACTTCCCTATAAGGTAGCCGATATTGGCCTGGCCGCTTGGGGCCGCAAAGCGCTGGACATCGCCGAGAATGAGATGCCCGGCCTGATGCGTATGCGTGAAATGTACAGCGCCAGTAAACCGCTGAAGGGGGCGCGCATTGCGGGTTGTTTACACATGACCGTAGAGACCGCTGTACTTATTGAAACCCTTGTTGCATTGGGAGCGGAAGTACGTTGGTCCTCATGTAATATCTTCAGCACTCAAGACCATGCAGCTGCCGCAATTGCCAAGGCGGGTATCCCGGTATTCGCTTGGAAAGGGGAGACAGATGAAGAGTATCTGTGGTGCATTGAACAGACATTACATTTCAAGGATGGTCCTCTTAACATGATTCTTGATGATGGGGGAGACCTTACGAACTTAATCCACACGAAATACCCACAGCTTCTGTCAGGAATCCGTGGAATCTCAGAAGAGACAACCACAGGAGTTCACAACTTGTACAAAATGATGAGCAATGGGATTTTGAAAGTGCCAGCGATCAACGTGAACGATTCCGTAACTAAGAGCAAATTCGATAACCTGTACGGCTGCCGTGAATCGTTGATCGACGGAATTAAGCGCGCGACCGATGTGATGATTGCGGGGAAGGTTGCGGTAGTTGCGGGGTATGGGGACGTAGGTAAGGGGTGCGCGCAAGCACTTCGCGGTTTTGGAGCTCGTGTAATTATCACAGAGATTGACCCTATTAATGCTCTGCAAGCAGCGATGGAGGGGTACGAAGTCACGACCATGGATGAGGCGTGCAAGGAGGGTAATATTTTCGTCACAACCACGGGCTGTGTAGACATCATTTTAGGGCGTCACTTCGAACAAATGAAGGATGATGCCATTGTGTGTAACATCGGTCATTTCGATGTGGAGATCGACGTTAAGTGGCTTAACGAAAATGCAGTAGAAAAAGTCAACATCAAACCACAGGTGGACCGTTATTGGCTTAAGAATGGGCGTCGTATCATCCTGCTTGCCGAGGGTCGTTTAGTCAACCTTGGGTGTGCGATGGGCCACCCCTCTTTCGTAATGTCCAATAGCTTCACAAATCAAGTGATGGCTCAGATCGAACTGTGGACGCATCCGGACAAATATCCCGTAGGAGTTCATTTTTTGCCGAAAAAGTTGGATGAGGCCGTGGCGGAAGCACATCTTGGAAAACTGAATGTAAAATTGACTAAATTGACTGAAAAGCAAGCGCAATATTTAGGTATGCCGATCAACGGCCCTTTCAAACCCGACCATTATCGTTATTAA

Protein sequence of *Mm*SAHH [432 aa]

MSDKLPYKVADIGLAAWGRKALDIAENEMPGLMRMREMYSASKPLKGARIAGCLHMTVETAVLIETLVALGAEVRWSSCNIFSTQDHAAAAIAKAGIPVFAWKGETDEEYLWCIEQTLHFKDGPLNMILDDGGDLTNLIHTKYPQLLSGIRGISEETTTGVHNLYKMMSNGILKVPAINVNDSVTKSKFDNLYGCRESLIDGIKRATDVMIAGKVAVVAGYGDVGKGCAQALRGFGARVIITEIDPINALQAAMEGYEVTTMDEACKEGNIFVTTTGCVDIILGRHFEQMKDDAIVCNIGHFDVEIDVKWLNENAVEKVNIKPQVDRYWLKNGRRIILLAEGRLVNLGCAMGHPSFVMSNSFTNQVMAQIELWTHPDKYPVGVHFLPKKLDEAVAEAHLGKLNVKLTKLTEKQAQYLGMPINGPFKPDHYRY

Nucleotide sequence of *Ec*MAT [1155 bp]

ATGGCAAAACACCTTTTTACGTCCGAGTCCGTCTCTGAAGGGCATCCTGACAAAATTGCTGACCAAATTTCTGATGCCGTTTTAGACGCGATCCTCGAACAGGATCCGAAAGCACGCGTTGCTTGCGAAACCTACGTAAAAACCGGCATGGTTTTAGTTGGCGGCGAAATCACCACCAGCGCCTGGGTAGACATCGAAGAGATCACCCGTAACACCGTTCGCGAAATTGGCTATGTGCATTCCGACATGGGCTTTGACGCTAACTCCTGTGCGGTTCTGAGCGCTATCGGCAAACAGTCTCCTGACATCAACCAGGGCGTTGACCGTGCCGATCCGCTGGAACAGGGCGCGGGTGACCAGGGTCTGATGTTTGGCTACGCAACTAATGAAACCGACGTGCTGATGCCAGCACCTATCACCTATGCACACCGTCTGGTACAGCGTCAGGCTGAAGTGCGTAAAAACGGCACTCTGCCGTGGCTGCGCCCGGACGCGAAAAGCCAGGTGACTTTTCAGTATGACGACGGCAAAATCGTTGGTATCGATGCTGTCGTGCTTTCCACTCAGCACTCTGAAGAGATCGACCAGAAATCGCTGCAAGAAGCGGTAATGGAAGAGATCATCAAGCCAATTCTGCCCGCTGAATGGCTGACTTCTGCCACCAAATTCTTCATCAACCCGACCGGTCGTTTCGTTATCGGTGGCCCAATGGGTGACTGCGGTCTGACTGGTCGTAAAATTATCGTTGATACCTACGGCGGCATGGCGCGTCACGGTGGCGGTGCATTCTCTGGTAAAGATCCATCAAAAGTGGACCGTTCCGCAGCCTACGCAGCACGTTATGTCGCGAAAAACATCGTTGCTGCTGGCCTGGCCGATCGTTGTGAAATTCAGGTTTCCTACGCAATCGGCGTGGCTGAACCGACCTCCATCATGGTAGAAACTTTCGGTACTGAGAAAGTGCCTTCTGAACAACTGACCCTGCTGGTACGTGAGTTCTTCGACCTGCGCCCATACGGTCTGATTCAGATGCTGGATCTGCTGCACCCGATCTACAAAGAAACCGCAGCATACGGTCACTTTGGTCGTGAACATTTCCCGTGGGAAAAAACCGACAAAGCGCAGCTGCTGCGCGATGCTGCCGGTCTGAAGTAA

Protein sequence of *Ec*MAT [384 aa]

MAKHLFTSESVSEGHPDKIADQISDAVLDAILEQDPKARVACETYVKTGMVLVGGEITTSAWVDIEEITRNTVREIGYVHSDMGFDANSCAVLSAIGKQSPDINQGVDRADPLEQGAGDQGLMFGYATNETDVLMPAPITYAHRLVQRQAEVRKNGTLPWLRPDAKSQVTFQYDDGKIVGIDAVVLSTQHSEEIDQKSLQEAVMEEIIKPILPAEWLTSATKFFINPTGRFVIGGPMGDCGLTGRKIIVDTYGGMARHGGGAFSGKDPSKVDRSAAYAARYVAKNIVAAGLADRCEIQVSYAIGVAEPTSIMVETFGTEKVPSEQLTLLVREFFDLRPYGLIQMLDLLHPIYKETAAYGHFGREHFPWEKTDKAQLLRDAAGLK**Characterization of Products**

**(*S*)-1-(3,4-dimethoxybenzyl)-1,2,3,4-tetrahydroisoquinoline-6,7-diol ((*S*)-3a)**

White soild.

**^1^H NMR (600 MHz, Methanol-*d*_4_)** δ 6.85 (d, *J* = 7.9 Hz, 1H), 6.77 – 6.75 (m, 2H), 6.52 (d, *J* = 4.9 Hz, 2H), 4.54 (dd, *J* = 8.5, 5.9 Hz, 1H), 3.73 (s, 3H), 3.71 (s, 3H), 3.36-3.28 (m, 1H), 3.29 (dd, *J* = 14.5, 5.7 Hz, 1H), 3.18 – 3.13 (m, 1H), 2.92 (dd, *J* = 14.4, 8.6 Hz, 1H), 2.89 – 2.85 (m, 1H), 2.81 (dt, *J* = 17.1, 6.3 Hz, 1H) ppm.

**^13^C NMR (101 MHz, Methanol-*d*_4_)** δ 149.6, 148.8, 145.5, 144.4, 127.7, 122.3, 122.2, 121.7, 114.8, 112.9, 112.8, 112.0, 56.3, 55.1, 55.0, 39.5, 39.4, 24.3 ppm.

**HRMS** (ESI) for [C_18_H_21_NO_4_+H]^+^: 316.1543 (calculated), 316.1553 (found).

**(*S*)-1-(3,4-dimethoxybenzyl)-6-methoxy-1,2,3,4-tetrahydroisoquinolin-7-ol((*S*)-4a)**

Yellow soild.

**^1^H NMR (400 MHz, Methanol-*d_4_*)** δ 6.85 (d, J = 8.0 Hz, 1H), 6.77-6.74 (m, 2H), 6.68 (s, 1H), 6.56 (s, 1H), 4.56 (dd, J = 8.4, 5.9 Hz, 1H), 3.75 (s, 3H), 3.72 (s, 3H), 3.70 (s, 3H),3.40-3.34 (m, 1H), 3.30 (dd, J = 14.5, 5.7 Hz, 1H), 3.18 – 3.15 (m, 1H), 2.99 – 2.92 (m, 2H), 2.90 – 2.84 (m, 1H) ppm.

**^13^C** **NMR (101 MHz, Methanol-*d_4_*)** δ 148.1, 147.4, 146.5, 144.0, 126.3, 122.2, 120.9, 120.4, 111.4, 111.4, 110.6, 109.9, 54.8, 53.7, 53.6, 53.6, 38.0, 37.9, 23.1 ppm.

**HRMS** (ESI) for [C_19_H_23_NO_4_+H]^+^: 330.1700 (calculated), 330.1705 (found).

**(*S*)-Tetrahydropapaverine ((*S*)-5a)**

Yellow liquid, 98% *ee*

**^1^H NMR** **(400 MHz, Chloroform-*d_4_*)** δ 6.83 (d, J = 8.1 Hz, 1H), 6.80 (dd, J = 8.0, 1.5 Hz, 1H), 6.76 (d, J = 1.3 Hz, 1H), 6.67 (s, 1H), 6.60 (s, 1H), 4.12 – 4.09 (m, 1H), 3.87 (s, 3H), 3.86 (s, 6H), 3.84 (s, 3H), 3.19 (ddd, J = 14.2, 8.9, 5.1 Hz, 2H), 2.94 – 2.83 (m, 2H), 2.80 – 2.65 (m, 2H) ppm.

**^13^C NMR (151 MHz, Chloroform-*d_4_*)** δ 148.9, 147.6, 147.4, 146.9, 131.4, 130.5, 127.5, 121.4, 112.3, 111.8, 111.2, 109.3, 56.9, 56.0, 55.9, 55.8, 55.8, 42.2, 41.0, 29.6 ppm.

**HRMS** (ESI) for [C_20_H_24_NO_4_+H]^+^: 344.1856 (calculated), 344.1869 (found).

**HPLC**: Chiral Pak^®^ OD-H, n-hexane (0.1% diethylamine)/isopropanol (0.1% diethylamine) 60/40, flow rate = 1.2 mL/min, uv-vis λ = 280 nm, *t*_R1_ = 11.2 min (major), *t*_R2_ = 16.5 min (minor).


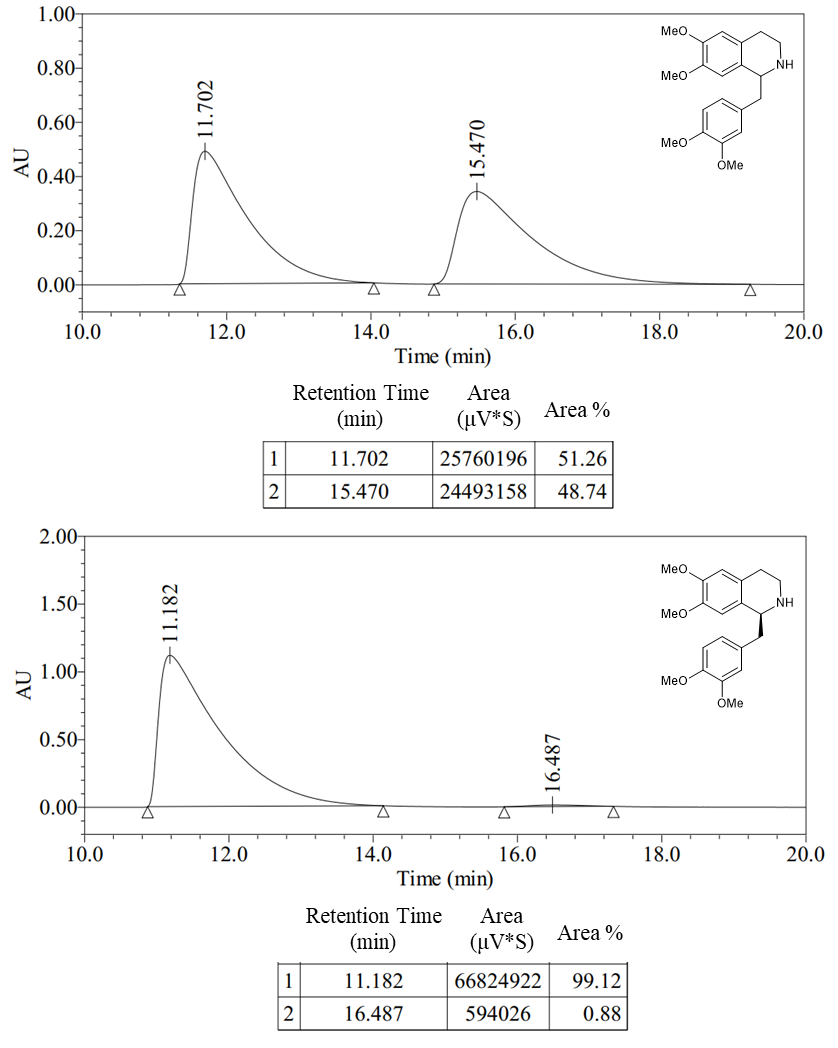


**(*S*)-1-(benzo[d] [1,3] dioxol-5-ylmethyl)-1,2,3,4-tetrahydroisoquinoline-6,7-diol ((*S*)-3b)**

White soild.

**^1^H NMR (400 MHz, Methanol-*d*_4_)** δ 6.84 (d, *J* = 1.2 Hz, 1H), 6.82 (d, *J* = 8.4 Hz, 1H), 6.77 (dd, *J* = 8.0, 1.1 Hz, 1H), 6.62 (s, 2H), 5.96 (s, 2H), 4.59 (dd, *J* = 9.1, 5.6 Hz, 1H), 3.50 – 3.44 (m, 1H), 3.39 (dd, *J* = 14.5, 5.6 Hz, 1H), 3.28 – 3.22 (m, 1H), 3.03 – 2.87 (m, 3H) ppm.

**^13^C NMR (151 MHz, Methanol-*d*_4_)** δ 149.9, 148.8, 147.0, 145.9, 130.1, 124.0, 123.7, 123.6, 116.2, 114.2, 110.5, 109.7, 102.6, 57.9, 41.0, 40.9, 25.8 ppm.

**HRMS** (ESI) for [C_17_H_17_NO_4_+H]^+^: 300.1230 (calculated), 300.1234 (found).

**(*S*)-1-(benzo[d] [1,3] dioxol-5-ylmethyl)-6-methoxy-1,2,3,4-tetrahydroisoquinolin-7-ol ((*S*)-4b)**

Yellow soild.

**^1^H NMR (400 MHz, Chloroform-*d*)** δ 6.79 (s, 1H), 6.77 (d, *J* = 7.8 Hz, 1H), 6.74 (d, *J* = 1.2 Hz, 1H), 6.70 (dd, *J* = 7.8, 1.3 Hz, 1H), 6.57 (s, 1H), 5.94 (s, 2H), 4.03 (dd, *J* = 10.0, 3.6 Hz, 1H), 3.85 (s, 3H), 3.21 – 3.13 (m, 1H), 3.11 (dd, *J* = 13.8, 3.6 Hz, 1H), 2.92 – 2.86 (m, 1H), 2.80 – 2.65 (m, 3H) ppm.

**^13^C NMR (101 MHz, Chloroform-*d*)** δ 147.8, 146.1, 145.2, 143.7, 132.8, 131.2, 126.6, 122.3, 112.0, 111.2, 109.5, 108.3, 100.9, 56.9, 55.9, 42.1, 40.9, 29.6 ppm.

**HRMS** (ESI) for [C_18_H_19_NO_4_+H]^+^: 314.1387 (calculated), 314.1385 (found).

**(*S*)-1-(1,3-benzodioxol-5-ylmethyl)-1,2,3,4-tetrahydro-6,7-dimethoxyisoquinoline ((*S*)-5b)**

Yellow soild, >99% *ee*

**^1^H NMR (400 MHz, Chloroform-*d*)** δ 6.83 – 6.77 (m, 2H), 6.73 (dd, *J* = 7.8, 1.7 Hz, 1H), 6.66 (s, 1H), 6.62 (s, 1H), 5.97 (d, *J* = 1.4 Hz, 2H), 4.12 (dd, *J* = 9.6, 4.3 Hz, 1H), 3.88 (s, 3H), 3.86 (s, 3H), 3.23 (dt, *J* = 11.7, 5.7 Hz, 1H), 3.15 (dd, *J* = 13.8, 4.3 Hz, 1H), 2.99 – 2.90 (m, 1H), 2.86 (dd, *J* = 13.7, 9.6 Hz, 1H), 2.82 – 2.67 (m, 2H) ppm.

**^13^C NMR (151 MHz, Chloroform-*d*)** δ 147.8, 147.4, 147.0, 146.2, 132.7, 130.2, 127.2, 122.3, 111.7, 109.5, 109.3, 108.4, 100.9, 56.9, 56.0, 55.8, 42.3, 40.6, 29.4 ppm.

**HRMS** (ESI) for [C_19_H_21_NO_4_+H]^+^ 328.1543 (calculated), 328.1550 (found).

**HPLC**: Chiral Pak^®^ OD-H, n-hexane (0.1% diethylamine)/isopropanol (0.1% diethylamine) 90/10, flow rate = 1.0 mL/min, uv-vis λ = 280 nm, *t*_R_ = 37.0 min.


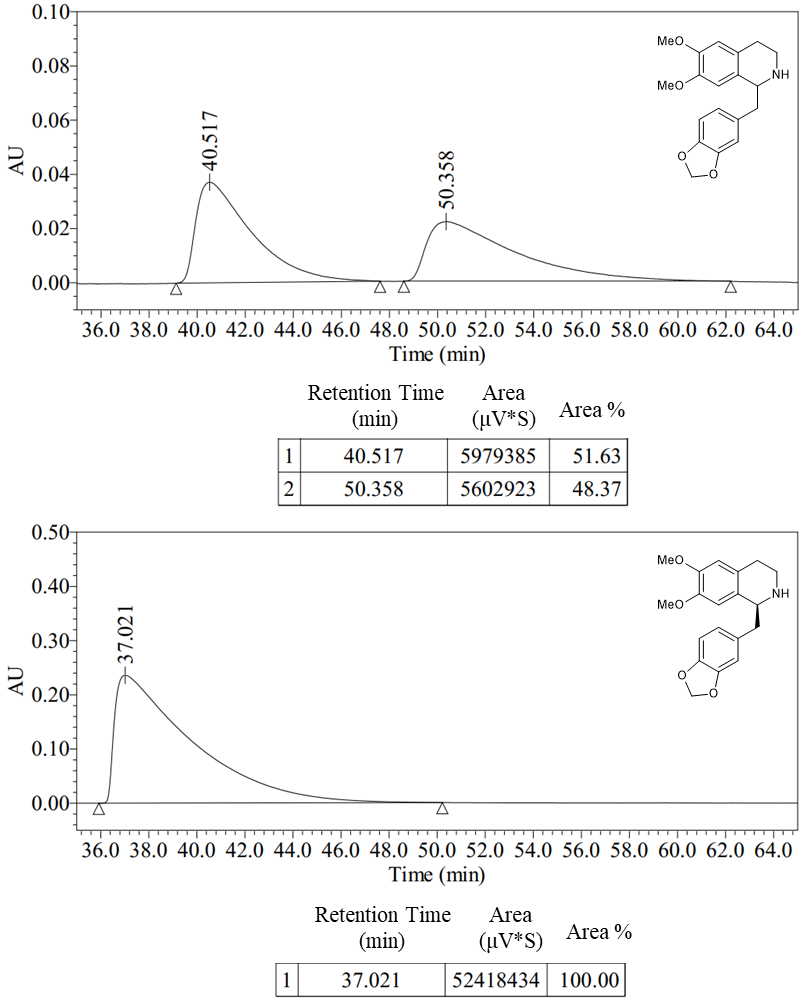


**(*S*)-1-(3,4-diethoxybenzyl)-1,2,3,4-tetrahydroisoquinoline-6,7-diol ((*S*)-3c)**

White soild.

**^1^H NMR (600 MHz, Methanol-*d*_4_)** δ 6.94 (d, *J* = 8.0 Hz, 1H), 6.84 (s, 1H), 6.84 – 6.81 (m, 1H), 6.61 (d, *J* = 4.9 Hz, 2H), 4.62 (dd, *J* = 8.5, 5.7 Hz, 1H), 4.04 (dq, *J* = 17.3, 7.0 Hz, 4H), 3.45 – 3.41 (m, 1H), 3.36 (dd, *J* = 14.5, 5.7 Hz, 1H), 3.27 – 3.20 (m, 1H), 3.00 (dd, *J* = 14.5, 8.6 Hz, 1H), 2.97 – 2.94 (m, 1H), 2.90 (dt, *J* = 17.0, 6.3 Hz, 1H), 1.41 – 1.37 (m, 6H) ppm.

**^13^C NMR (151 MHz, Methanol-*d*_4_)** δ 150.5, 149.7, 146.9, 145.9, 129.2, 123.7, 123.6, 123.2, 116.2, 116.0, 115.3, 114.3, 65.8, 65.7, 57.7, 40.9, 40.8, 25.7, 15.2, 15.2 ppm.

**HRMS** (ESI) for [C_20_H_25_NO_4_+H]^+^ 344.1856 (calculated), 344.1854 (found).

**(*S*)-1-(3,4-diethoxybenzyl)-6-methoxy-1,2,3,4-tetrahydroisoquinolin-7-ol ((*S*)-4c)**

Yellow soild.

**^1^H NMR (600 MHz, Chloroform-*d*)** δ 6.81 (d, *J* = 8.0 Hz, 1H), 6.76 (s, 1H), 6.73 – 6.71 (m, 2H), 6.52 (s, 1H), 4.08 – 4.04 (m, 4H), 4.02 – 4.00 (m, 1H), 4.01 (d, *J* = 7.0 Hz, 1H), 3.74 (s, 3H), 3.19 – 3.15 (m, 1H), 3.08 (dd, *J* = 13.8, 3.7 Hz, 1H), 2.88 – 2.84 (m, 1H), 2.78 – 2.71 (m, 2 H), 2.66 (dt, *J* = 15.9, 5.1 Hz, 1H), 1.42 (dt, *J* = 14.4, 7.0 Hz, 6H) ppm.

**^13^C NMR (151 MHz, Chloroform-*d*)** δ 148.7, 147.3, 145.7, 144.1, 131.3, 130.6, 126.2, 121.5, 114.5, 113.6, 112.5, 111.2, 64.6, 64.4, 56.6, 55.7, 41.6, 41.0, 29.3, 14.9, 14.8 ppm.

**HRMS** (ESI) for [C_21_H_27_NO_4_+H]^+^ 358.2013 (calculated), 358.2028 (found).

**(*S*)-(1-(3,4-diethoxybenzyl)-6,7-dimethoxy-1,2,3,4-tetrahydroisoquinoline) ((*S*)-5c)**

White soild, >99% *ee*

**^1^H NMR (600 MHz, Chloroform-*d*)** δ 6.84 (d, *J* = 8.6 Hz, 1H),6.77 – 6.76 (m, 2H), 6.65 (s, 1H), 6.59 (s, 1H), 4.12 (dd, *J* = 9.2, 4.4 Hz, 1H), 4.09 – 4.05 (m, 4H), 3.86 (s, 3H), 3.83 (s, 3H), 3.20 (dt, *J* = 11.5, 5.5 Hz, 1H), 3.14 (dd, *J* = 13.7, 4.4 Hz, 1H), 2.92 – 2.88 (m, 1H), 2.84 (dd, *J* = 13.7, 9.3 Hz, 1H), 2.76 (dt, *J* = 22.7, 5.9 Hz, 1H), 2.68 (dt, *J* = 16.0, 5.4 Hz, 1H), 1.46 – 1.42 (m, 6H) ppm.

**^13^C NMR (151 MHz, Chloroform-*d*)** δ 148.7, 147.3, 147.3, 146.9, 131.5, 130.5, 127.4, 121.6, 114.5, 113.6, 111.7, 109.3, 64.6, 64.5, 56.9, 56.0, 55.8, 42.2, 40.9, 29.6, 14.9, 14.9 ppm.

**HRMS** (ESI) for [C_22_H_29_NO_4_+H]^+^ 372.2169 (calculated), 372.2158 (found).

**HPLC**: Chiral Pak^®^ OD-H, n-hexane (0.1% diethylamine)/isopropanol (0.1% diethylamine) 65/35, flow rate = 1.0 mL/min, uv-vis λ = 280 nm, *t*_R_ = 13.1 min.


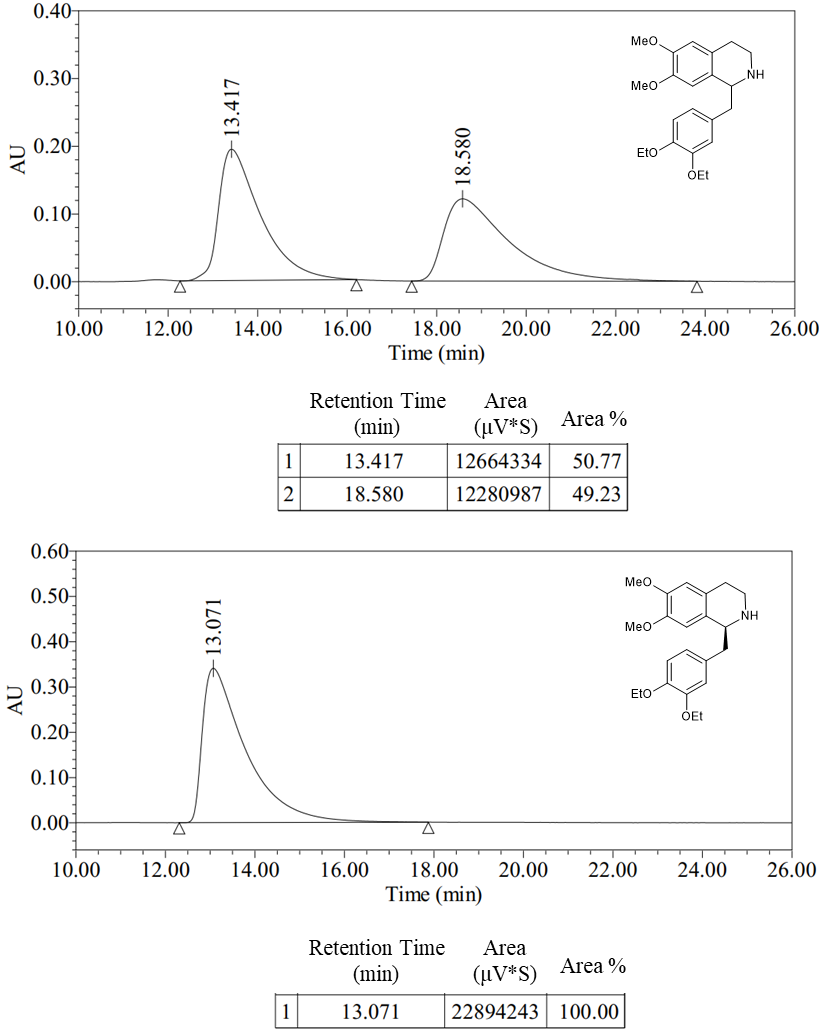


## General procedure for the synthesis of *rac*-5


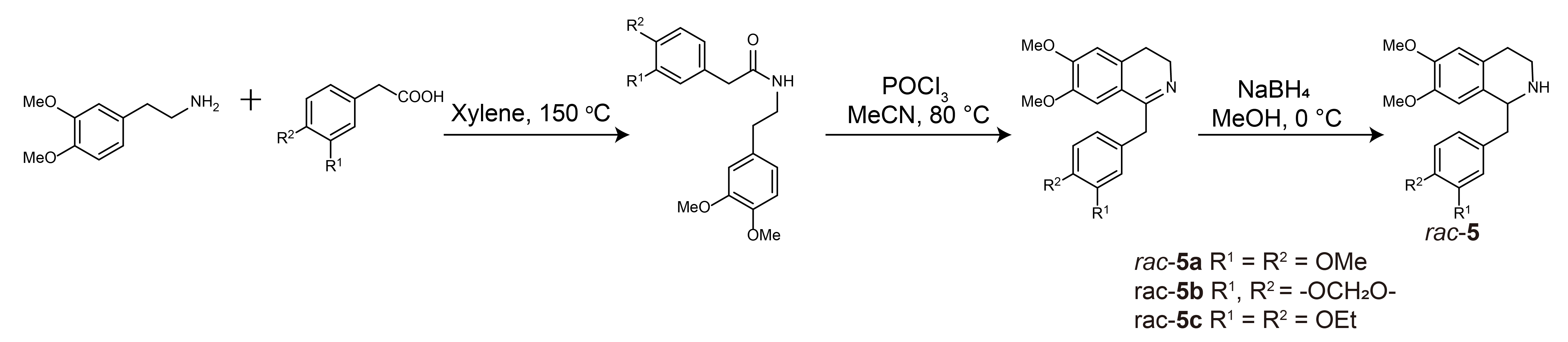


Compounds *rac*-**5a-c** were synthesized according to the literatures^[1,2]^ with modification. 3,4-Dimethoxyphenylethylamine (5 mmol, 1 eq) and substrate **1** (1 eq) were added in xylene (10 mL) to an oven-dried 100 mL round bottom flask. Subsequently, the mixture was refluxed at 150 ^o^C for 24 h. After the reaction was completed, xylene was removed under reduced pressure to afford the crude intermediate used in the next step without further purification.

Then, the residue was dissolved in acetonitrile (10 mL), followed by the addition of POCl_3_ (5 eq). The resulting mixture was refluxed at 80 °C for 2 h. After removing the solvent under reduced pressure, the residue was neutralized using saturated aqueous Na_2_CO_3_, and extracted with dichloromethane for three times. The combined organic layers were combined, washed with brine, dried over anhydrous Na_2_SO_4_, concentrated under reduced pressure, and used in the next step without further purification.

Next, the residue was dissolved in methanol (10 mL) under an argon atmosphere. Sodium borohydride (3 eq) was added into the mixture at 0 ^o^C, which was stirred for 30 min. Subsequently, the mixture was added with water (5 mL) extracted with dichloromethane for three times, and purified by silica gel column chromatography with petroleum ether/ethyl acetate/triethylamine, to afford the desired products.

## General procedure for the synthesis of 6


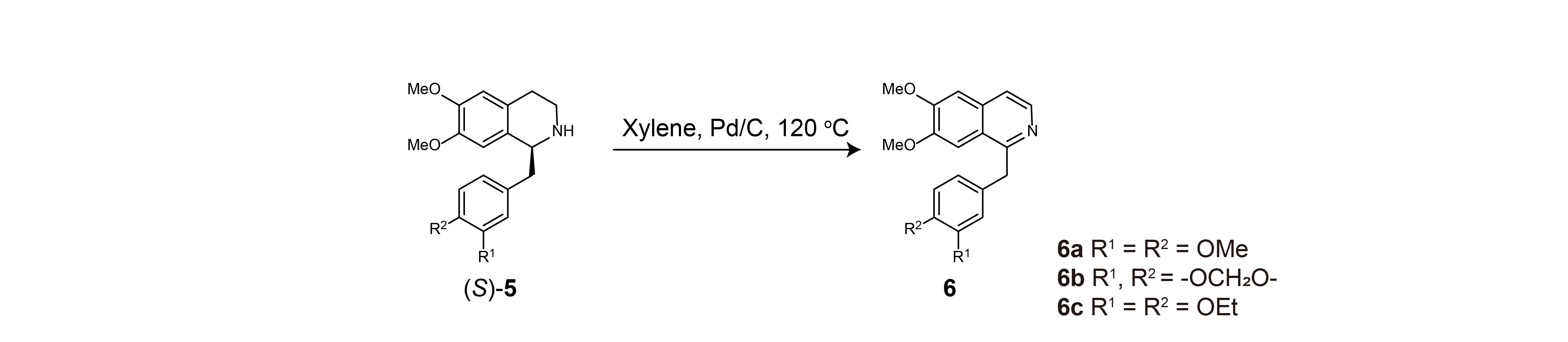


Substrate (*S*)-**5** (0.1 mmol, 1 eq) and Pd/C (1 eq) suspended in xylene (3 mL) was added in an oven-dried 25 mL round bottom flask. Then, the mixture was refluxed at 120 ^o^C for 24 h under argon atmosphere. After the reaction was completed, Pd/C was removed by filtraion, and xylene was removed under reduced pressure^[3]^. Then, the product was purified by silica gel column chromatography with petroleum ether/ethyl acetate/triethylamine, to afford the desired products.

For the gram-scale preparation of **6a**, (*S*)-**5a** (2 g), Pd/C (620 mg) and xylene (175 mL) were add in an oven-dried 1000 mL round bottom flask, which was refluxed at 120 ^o^C for 24 h under argon atmosphere. Pd/C was removed by filtraion, and xylene was removed under reduced pressure. Then, the product was purified by silica gel column chromatography with petroleum ether/ethyl acetate/triethylamine, to afford the desired products.

**Papaverine (6a)**

Yellow soild, 29.2 mg, 86% yield.

**^1^H NMR (400 MHz, Chloroform-*d*)** δ 8.37 (d, *J* = 5.7 Hz, 1H), 7.42 (d, *J* = 5.6 Hz, 1H), 7.34 (s, 1H), 7.05 (s, 1H), 6.82 (d, *J* = 6.7 Hz, 2H), 6.76 (d, *J* = 8.8 Hz, 1H), 4.53 (s, 2H), 4.00 (s, 3H), 3.90 (s, 3H), 3.82 (s, 3H), 3.77 (s, 3H) ppm.

**^13^C NMR (151 MHz, Chloroform-*d*)** δ 157.8, 152.4, 149.8, 149.0, 147.5, 141.0, 133.4, 132.3, 122.9, 120.5, 118.7, 111.9, 111.2, 105.3, 104.2, 56.0, 55.9, 55.9, 55.8, 42.2 ppm.

**HRMS** (ESI) for [C_20_H_21_NO_4_+H]^+^: 340.1543 (calculated), 340.1544 (found).

**1-(benzo[d][1,3]dioxol-5-ylmethyl)-6,7-dimethoxyisoquinoline (6b)**

Yellow soild, 22.9 mg, 71% yield.

**^1^H NMR (400 MHz, Chloroform-*d*)** δ 8.36 (d, *J* = 5.6 Hz, 1H), 7.42 (d, *J* = 5.6 Hz, 1H), 7.30 (s, 1H), 7.05 (s, 1H), 6.77 (d, *J* = 7.9 Hz, 1H), 6.72 – 6.70 (m, 2H), 5.87 (s, 2H), 4.51 (s, 2H), 4.00 (s, 3H), 3.91 (s, 3H) ppm.

**^13^C NMR (101 MHz, Chloroform-*d*)** δ 157.6, 152.5, 149.9, 147.8, 146.0, 141.0, 133.5, 133.5, 122.9, 121.4, 118.7, 109.0, 108.2, 105.3, 104.2, 100.9, 56.0, 55.9, 42.2 ppm.

**HRMS** (ESI) for [C_19_H_17_NO_4_+H]^+^: 324.1230 (calculated), 320.1245 (found).

**1-(3,4-diethoxybenzyl)-6,7-dimethoxyisoquinoline (6c)**

Yellow soild, 28.1 mg, 76% yield.

**^1^H NMR (600 MHz, Chloroform-*d*)** δ 8.29 (d, *J* = 5.6 Hz, 1H), 7.35 (d, *J* = 5.6 Hz, 1H), 7.25 (s, 1H), 6.97 (s, 1H), 6.74 (s, 1H), 6.69 (s, 2H), 4.44 (s, 2H), 3.95 (q, *J* = 7.0 Hz, 2H), 3.92 (s, 3H), 3.89 (q, *J* = 7.0 Hz, 2H), 3.81 (s, 3H), 1.32 (t, *J* = 7.0 Hz, 3H), 1.28 (t, *J* = 7.0 Hz, 3H) ppm.

**^13^C NMR (151 MHz, Chloroform-*d*)** δ 156.9, 151.3, 148.7, 147.7, 146.1, 140.0, 132.4, 131.3, 121.9, 119.6, 117.7, 113.0, 112.5, 104.2, 103.3, 63.5, 63.4, 55.0, 54.8, 41.3, 13.8, 13.8 ppm.

**HRMS** (ESI) for [C_22_H_25_NO_4_+H]^+^: 368.1856 (calculated), 368.1840 (found).

## General procedure for the synthesis of (*S*)-7

**
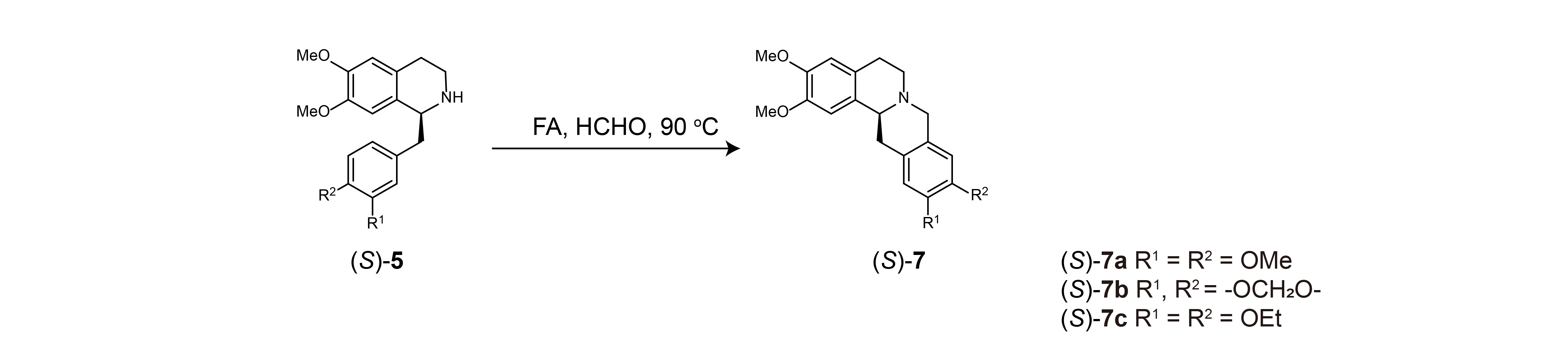
**

Substrates (*S*)-**5** (0.10 mmol, 1 eq), 37% formalin solution (0.6 mL), and formic acid (0.9 mL) were added in an oven-dried 25 mL round bottom flask, which was stirred at 90 ^o^C for 2 h^[4]^. After the reaction was completed, the mixture was neutralized using saturated aqueous Na_2_CO_3_, and extracted with dichloromethane for three times. The combined organic layers were washed with brine, dried over anhydrous Na_2_SO_4_, concentrated under reduced pressure, and purified by silica gel column chromatography with petroleum ether/ethyl acetate/triethylamine, to afford the desired products.

**(*S*)-xylopinine((*S*)-7a)**

Yellow soild, 28.7 mg, 81% yield, 98% *ee*

**^1^H NMR (400 MHz, Chloroform-*d*)** δ 6.75 (s, 1H), 6.67 (s, 1H), 6.62 (s, 1H), 6.58 (s, 1H), 3.95 (d, *J* = 14.5 Hz, 1H), 3.89 (s, 3H), 3.87 (s, 3H), 3.86 (s, 3H), 3.85 (s, 3H), 3.68 (d, *J* = 14.5 Hz, 1H), 3.59 (dd, *J* = 11.3, 3.9 Hz, 1H), 3.25 (dd, *J* = 15.8, 3.9 Hz, 1H), 3.19 – 3.10 (m, 2H), 2.84 (dd, *J* = 7.0, 0.6 Hz, 1H), 2.71 – 2.59 (m, 2H) ppm.

**^13^C NMR (101 MHz, Chloroform-*d*)** δ 147.6, 147.5, 147.4, 147.4, 129.8, 126.7, 126.3, 126.3, 111.3, 111.3, 109.0, 108.5, 59.6, 58.2, 56.0, 55.9, 55.9, 55.8, 51.4, 36.4, 29.0 ppm.

**HRMS** (ESI) for [C_21_H_25_NO_4_+H]^+^: 356.1856 (calculated), 356.1864 (found).

**HPLC**: Chiral Pak^®^ IA, n-hexane/isopropanol 65/35, flow rate = 1.0 mL/min, uv-vis λ = 280 nm, *t*_R1_ = 10.0 min (minor), *t*_R2_ = 20.3 min(major).


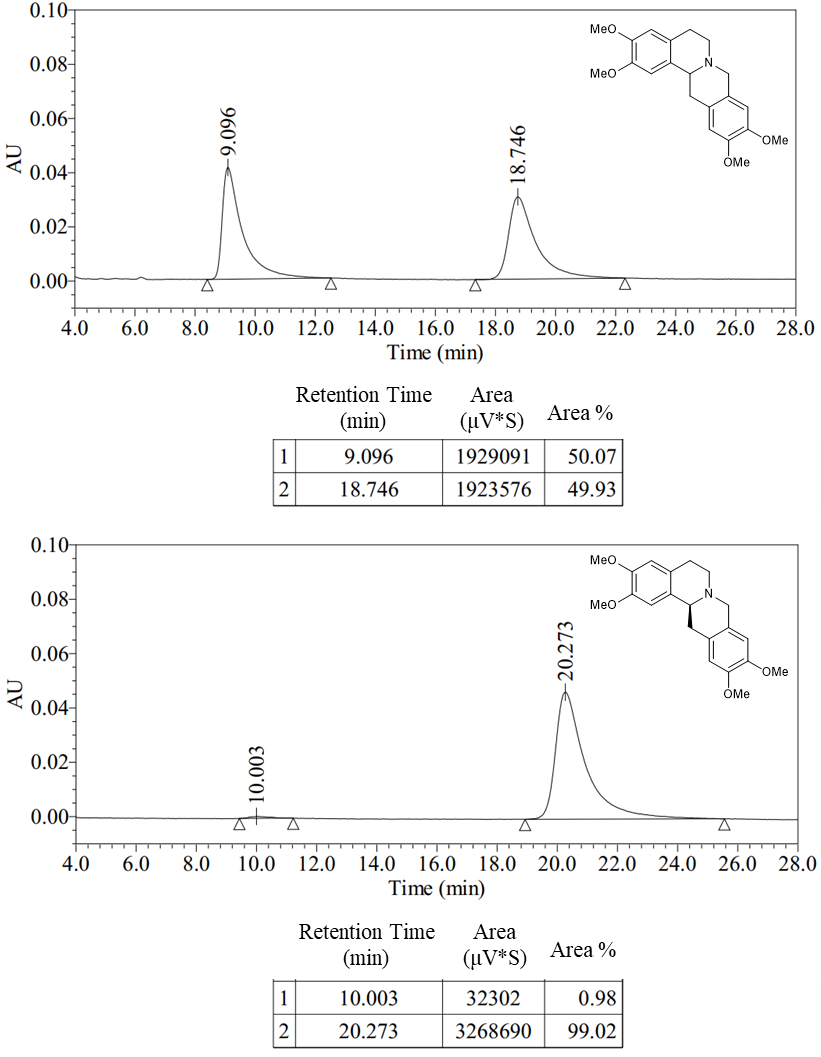


**(*S*)-isosinactine ((*S*)-7b)**

Yellow soild, 29.4 mg, 87% yield, >99% *ee*

**^1^H NMR (400 MHz, Chloroform-*d*)** δ 6.73 (s, 1H), 6.62 (d, *J* = 5.4 Hz, 2H), 6.55 (s, 1H), 5.90 (s, 2H), 3.94 – 3.90 (m, 4H), 3.87 (s, 3H), 3.64 (d, *J* = 14.6 Hz, 1H), 3.56 (dd, *J* = 11.3, 3.9 Hz, 1H), 3.22 (dd, *J* = 16.0, 3.9 Hz, 1H), 3.17 – 3.08 (m, 2H), 2.81 (dd, *J* = 15.5, 11.7 Hz, 1H), 2.69 – 2.56 (m, 2H) ppm.

**^13^C NMR (101 MHz, Chloroform-*d*)** δ 147.5, 147.4, 146.1, 145.8, 129.6, 127.3, 127.3, 126.7, 111.3, 108.5, 108.4, 106.1, 100.6, 59.5, 58.6, 56.1, 55.8, 51.3, 36.8, 29.0 ppm.

**HRMS** (ESI) for [C_20_H_21_NO_4_+H]^+^: 340.1543 (calculated), 340.1542 (found).

**HPLC**: Chiral Pak^®^ IA, n-hexane/isopropanol 65/35, flow rate = 1.0 mL/min, uv-vis λ = 280 nm, *t*_R_ = 13.7 min.


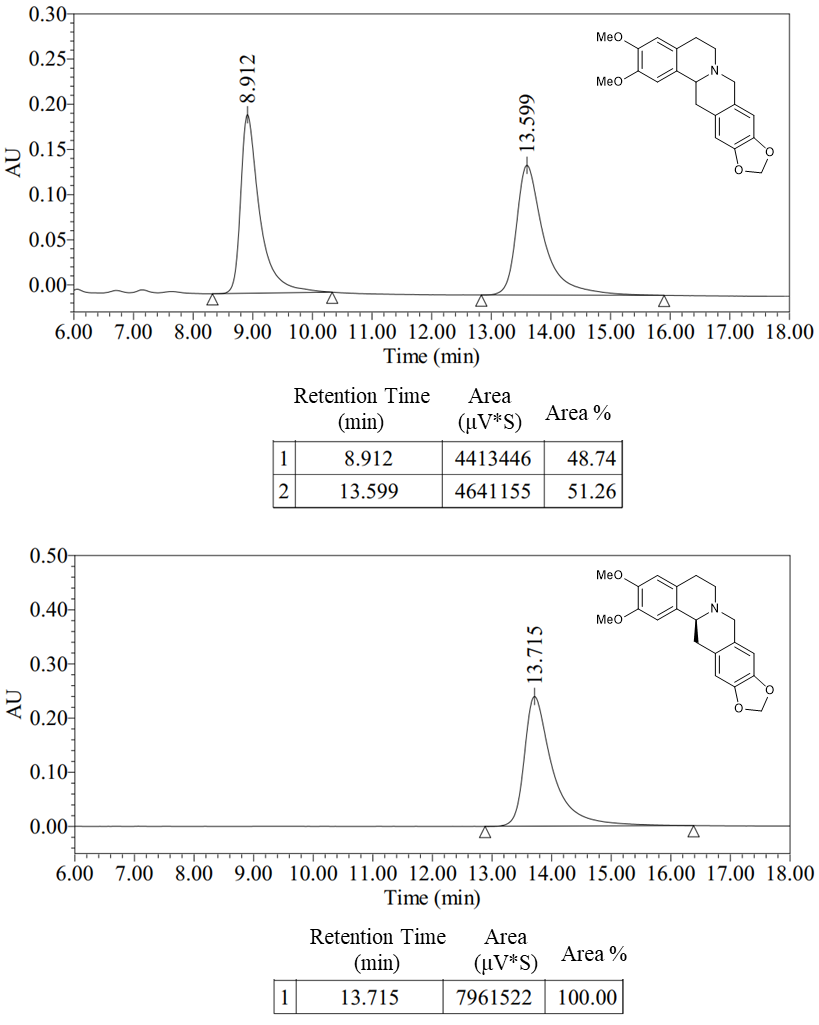


**(*S*)-10,11-diethoxy-2,3-dimethoxy-5,8,13,13a-tetrahydro-6H-isoquinolino[3,2-a]isoquinoline ((*S*)-7c)**

Yellow soild, 30.2 mg, 79% yield, 98% *ee*

**^1^H NMR (600 MHz, Chloroform-*d*)** δ 6.74 (s, 1H), 6.68 (s, 1H), 6.62 (s, 1H), 6.60 (s, 1H), 4.08 – 4.04 (m, 4H), 3.92 (d, *J* = 14.5 Hz, 1H), 3.89 (s, 3H), 3.87 (s, 3H), 3.67 (d, *J* = 14.4 Hz, 1H), 3.59 (dd, *J* = 11.4, 3.9 Hz, 1H), 3.24 (dd, *J* = 15.8, 4.0 Hz, 1H), 3.17 – 3.12 (m, 2H), 2.82 (dd, *J* = 15.4, 11.6 Hz, 1H), 2.69 – 2.60 (m, 2H), 1.45 – 1.43 (m, 6H) ppm.

**^13^C NMR (151 MHz, Chloroform-*d*)** δ 147.4, 147.4, 147.3, 147.0, 129.8, 126.7, 126.5, 126.5, 113.6, 111.3, 111.2, 108.4, 64.6, 64.5, 59.6, 58.3, 56.0, 55.8, 51.4, 36.4, 29.1, 14.9 ppm.

**HRMS** (ESI) for [C_23_H_29_NO_4_+H]^+^: 384.2169 (calculated), 384.2162 (found).

**HPLC**: Chiral Pak^®^ IA, n-hexane/isopropanol 65/35, flow rate = 1.0 mL/min, uv-vis λ = 280 nm, *t*_R1_ = 10.3 min (minor), *t*_R2_ = 19.0 min (major).


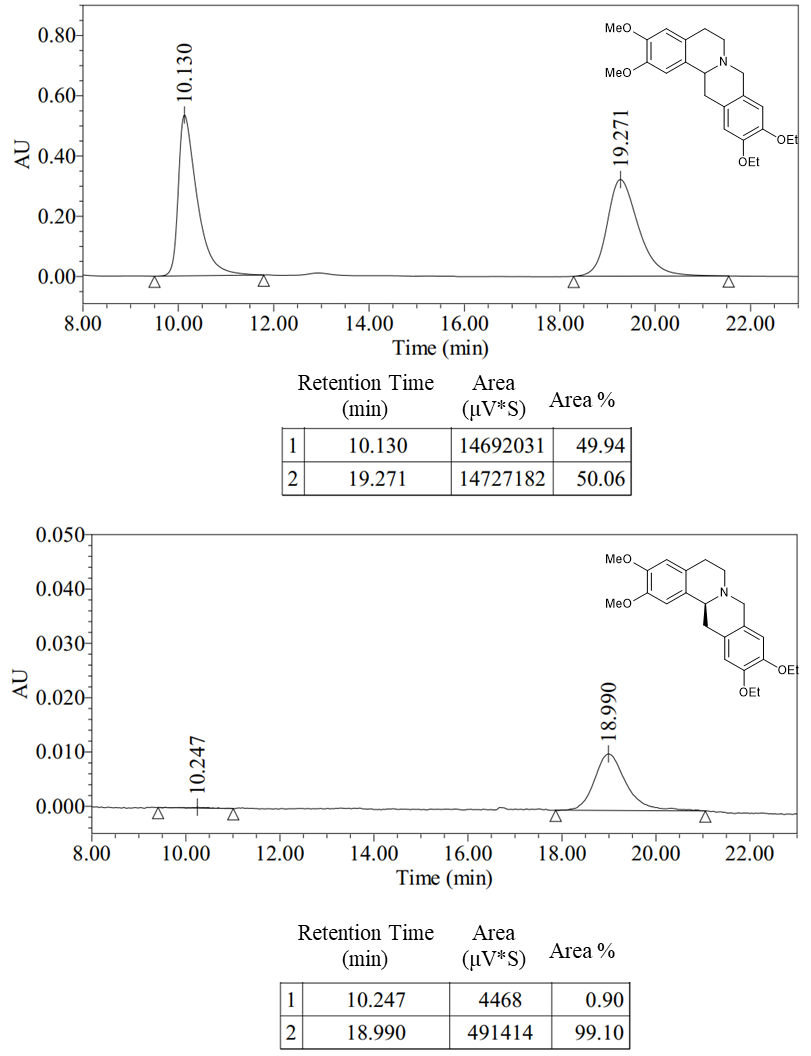


## The synthetic route for the synthesis of (*S*)-9 and (*S*)-10

**
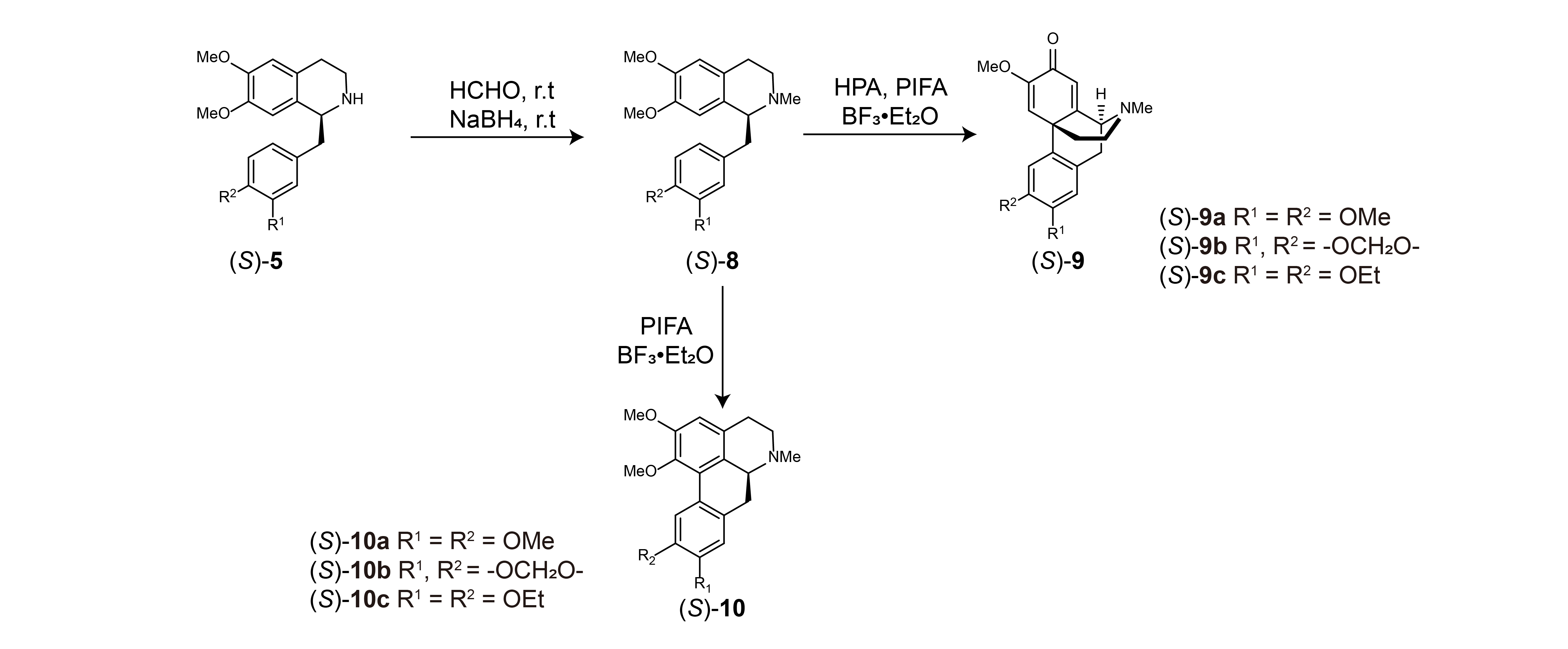
**

**General procedure for the synthesis of (*S*)-8**

In an oven-dried 50 mL round bottom flask, (*S*)-**5** (0.32 mmol, 1 eq) and 37% formalin solution (0.6 mL) were dissolved in methanol (1.8 mL). The mixture was stirred at room temperature for 4 h. Then, sodium borohydride (10 eq) was slowly added to the reaction mixture at 0 °C, followed by stirring for an additional 14 h at room temperature. After the reaction was completed, water (5 mL) was added to the mixture, which was subsequently extracted with dichloromethane for three times^[4]^. The combined organic layers were washed with brine, dried over anhydrous Na_2_SO_4_, concentrated under reduced pressure, and purified by silica gel column chromatography with petroleum ether/ethyl acetate/triethylamine, to afford the desired products.

**(*S*)-Laudanosine ((*S*)-8a)**

White soild, 84.8 mg, 79% yield, >99% *ee*

**^1^H NMR (400 MHz, Chloroform-*d*)** δ 6.77 (d, *J* = 8.1 Hz, 1H), 6.64 (dd, *J* = 8.1, 2.0 Hz, 1H), 6.60 (d, *J* = 2.0 Hz, 1H), 6.56 (s, 1H), 6.06 (s, 1H), 3.85 (s, 3H), 3.84 (s, 3H), 3.79 (s, 3H), 3.69 (dd, *J* = 7.8, 4.9 Hz, 1H), 3.58 (s, 3H), 3.20 – 3.12 (m, 2H), 2.87 – 2.79 (m, 1H), 2.79 – 2.73 (m, 2H), 2.59 (dt, *J* = 15.0, 4.4 Hz, 1H), 2.54 (s, 3H) ppm.

**^13^C NMR (101 MHz, Chloroform-*d*)** δ 148.6, 147.3, 147.3, 146.3, 132.5, 129.2, 126.0, 121.9, 113.0, 111.2, 111.1, 111.0, 64.9, 55.9, 55.8, 55.8, 55.6, 47.0, 42.7, 40.9, 25.5 ppm.

**HRMS** (ESI) for [C_21_H_27_NO_4_+H]^+^: 358.2013 (calculated), 358.2017 (found).

**HPLC**: Chiral Pak^®^ OD-H, n-hexane (0.1% diethylamine)/isopropanol (0.1% diethylamine) 40/60, flow rate = 1.0 mL/min, uv-vis λ = 280 nm, *t*_R_ = 6.8 min.


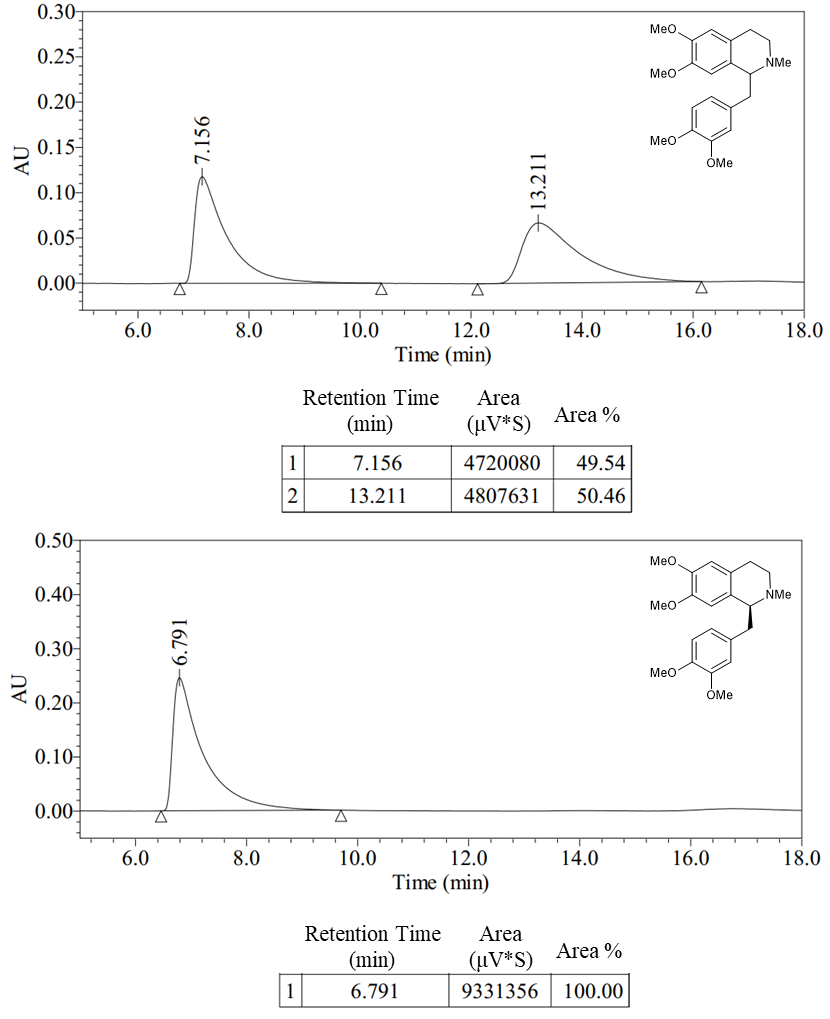


**(*S*)-1-(benzo[d][1,3]dioxol-5-ylmethyl)-6,7-dimethoxy-2-methyl-1,2,3,4-tetrahydroisoquinoline ((*S*)-8b)**

White soild, 91.9 mg, 90% yield, >99% *ee*

**^1^H NMR (400 MHz, Chloroform-*d*)** δ 6.70 (d, *J* = 7.9 Hz, 1H), 6.65 (d, *J* = 1.1 Hz, 1H), 6.55 (d, *J* = 8.2 Hz, 2H), 6.11 (s, 1H), 5.91 (d, *J* = 2.2 Hz, 2H), 3.84 (s, 3H), 3.66 (t, *J* = 6.4 Hz, 1H), 3.63 (s, 3H), 3.17 (ddd, *J* = 12.8, 8.3, 4.8 Hz, 1H), 3.09 (dd, *J* = 13.8, 5.4 Hz, 1H), 2.87 – 2.80 (m, 1H), 2.78 – 2.72 (m, 2H), 2.59 (dt, *J* = 15.6, 4.5 Hz, 1H), 2.51 (s, 3H) ppm.

**^13^C NMR (101 MHz, Chloroform-*d*)** δ 147.4, 147.3, 146.4, 145.7, 133.9, 129.3, 126.0, 122.6, 111.2, 111.0, 110.1, 107.9, 100.7, 64.9, 55.8, 55.6, 46.8, 42.7, 41.1, 25.4 ppm.

**HRMS** (ESI) for [C_20_H_23_NO_4_+H]^+^: 342.1700 (calculated), 342.1706 (found).

**HPLC**: Chiral Pak^®^ OD-H, n-hexane (0.1% diethylamine)/isopropanol (0.1% diethylamine) 85/15 flow rate = 1.0 mL/min, uv-vis λ = 280 nm, *t*_R1_ = 10.0 min (major), *t*_R2_ = 27.0 min (minor).


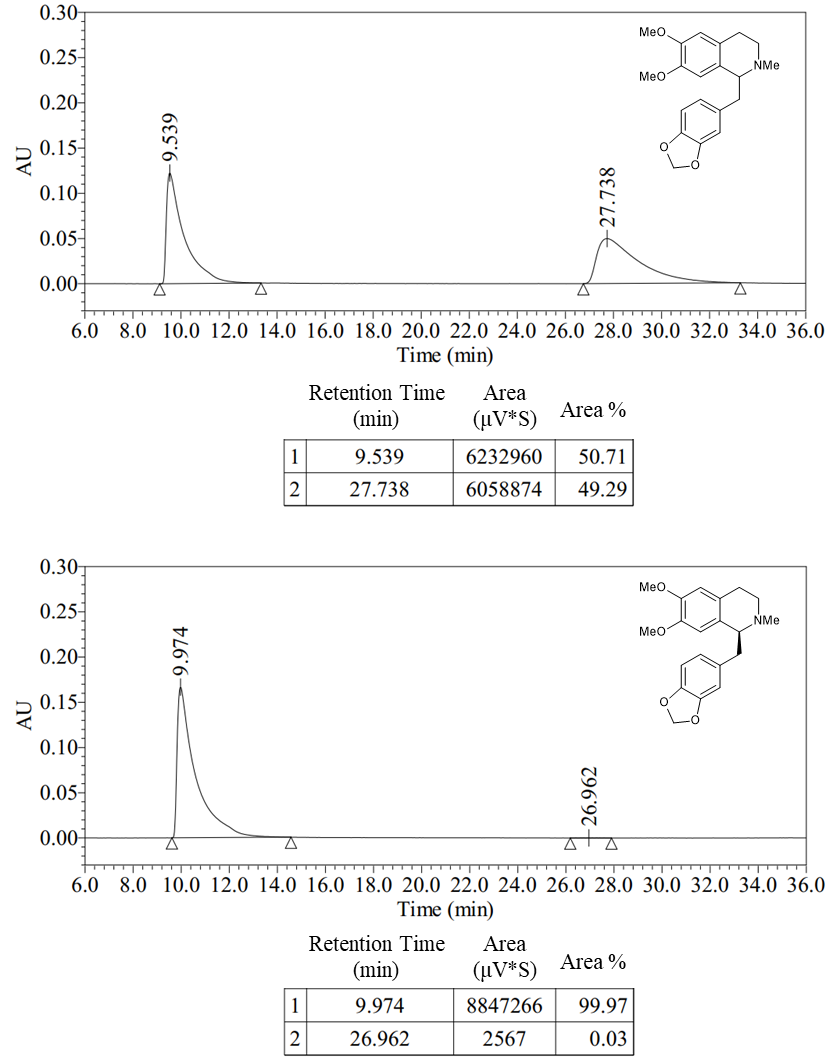


**(*S*)-1-(3,4-diethoxybenzyl)-6,7-dimethoxy-2-methyl-1,2,3,4-tetrahydroisoquinoline ((*S*)-8c)**

White soild, 100.2 mg, 87% yield, >99% *ee*

**^1^H NMR (600 MHz, Chloroform-*d*)** δ 6.77 (d, *J* = 8.1 Hz, 1H), 6.64 (d, *J* = 1.8 Hz, 1H), 6.60 (dd, *J* = 8.2, 1.7 Hz, 1H), 6.55 (s, 1H), 6.03 (s, 1H), 4.05 (qd, *J* = 7.0, 1.7 Hz, 2H), 4.00 (q, *J* = 7.2 Hz, 2H), 3.83 (s, 3H), 3.68 (dd, *J* = 8.0, 4.7 Hz, 1H), 3.56 (s, 3H), 3.19 – 3.12 (m, 2H), 2.85 – 2.80 (m, 1H), 2.78 – 2.72 (m, 2H), 2.59 (dt, *J* = 15.9, 4.7 Hz, 1H), 2.54 (s, 3H), 1.41 (dt, *J* = 13.9, 7.0 Hz, 6H) ppm.

**^13^C NMR (151 MHz, Chloroform-*d*)** δ 148.4, 147.2, 147.0, 146.2, 132.6, 129.3, 125.8, 122.1, 115.2, 113.4, 111.1, 110.9, 64.9, 64.6, 64.4, 55.7, 55.5, 47.0, 42.7, 40.9, 25.6, 14.9, 14.8 ppm.

**HRMS** (ESI) for [C_23_H_31_NO_4_+H]^+^: 386.2326 (calculated), 386.2316 (found).

**HPLC**: Chiral Pak^®^ OD-H, n-hexane (0.1% diethylamine)/isopropanol (0.1% diethylamine) 65/35 flow rate = 1.0 mL/min, uv-vis λ = 280 nm, *t*_R1_ = 6.8 min (major), *t*_R2_ = 12.7 min (minor).


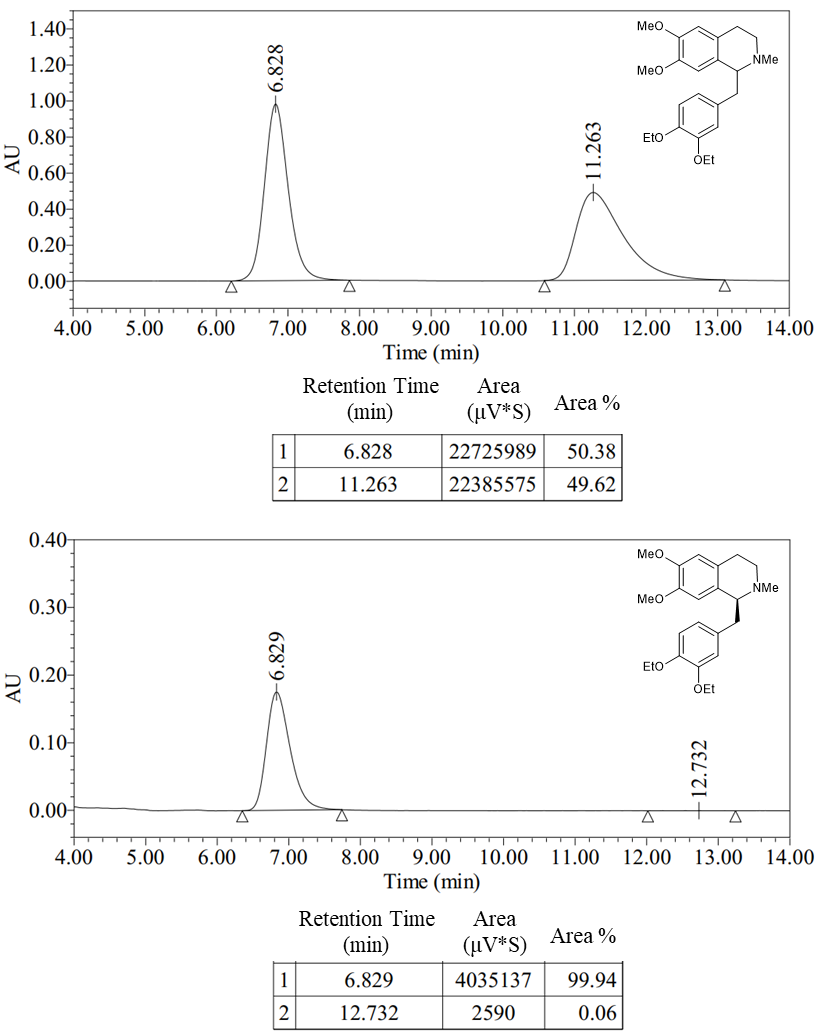


**General procedure for the synthesis of (*S*)-9**

In an oven-dried 50 mL round bottom flask, (*S*)-**8** (0.1 mmol, 1 eq), HPA (100 mg), and BF₃•Et₂O (5 eq) were dissolved in acetonitrile (4 mL), and stirred at -20 °C. Subsequently, PIFA (1 eq) was slowly added to the mixture, which was stirred for another 1 h with the temperature gradually warming from -20°C to 0 °C^[4]^. After the reaction was completed, the reaction was subsequently diluted by ethyl acetate with 10% triethylamine, quenched with saturated aqueous NaHCO_3_, and extracted with ethyl acetate for three times. The combined organic layers were washed with brine, dried over anhydrous Na_2_SO_4_, concentrated under reduced pressure, and purified by silica gel column chromatography using ethyl acetate/triethylamine, to afford the desired product.

**(*S*)-sebiferine ((*S*)-9a)**

Yellow soild, 20.5 mg, 60% yield, >99% *ee*

**^1^H NMR (600 MHz, Chloroform-*d*)** δ 6.81 (s, 1H), 6.64 (s, 1H), 6.36 (s, 1H), 6.34 (s, 1H), 3.89 (s, 3H), 3.87 (s, 3H), 3.81 (s, 3H), 3.71 (d, *J* = 6.1 Hz, 1H), 3.36 (d, *J* = 17.8 Hz, 1H), 3.06 (dd, *J* = 17.8, 6.2 Hz, 1H), 2.63 – 2.55 (m, 2H), 2.48 (s, 3H), 1.98 – 1.93 (m, 1H), 1.85 (dt, *J* = 12.8, 2.8 Hz, 1H) ppm.

**^13^C NMR (151 MHz, Chloroform-*d*)** δ 180.9, 161.6, 151.4, 148.4, 148.0, 130.0, 128.7, 122.3, 118.8, 110.4, 108.6, 60.9, 56.3, 55.9, 55.1, 45.7, 42.3, 41.7, 41.1, 32.7 ppm.

**HRMS** (ESI) for [C_20_H_23_NO_4_+H]^+^: 342.1700 (calculated), 342.1709 (found).

**HPLC**: Chiral Pak^®^ IA, n-hexane/isopropanol 50/50 flow rate = 1.2 mL/min, uv-vis λ = 280 nm, *t*_R1_ = 26.8 min (minor), *t*_R2_ = 40.3 min (major).


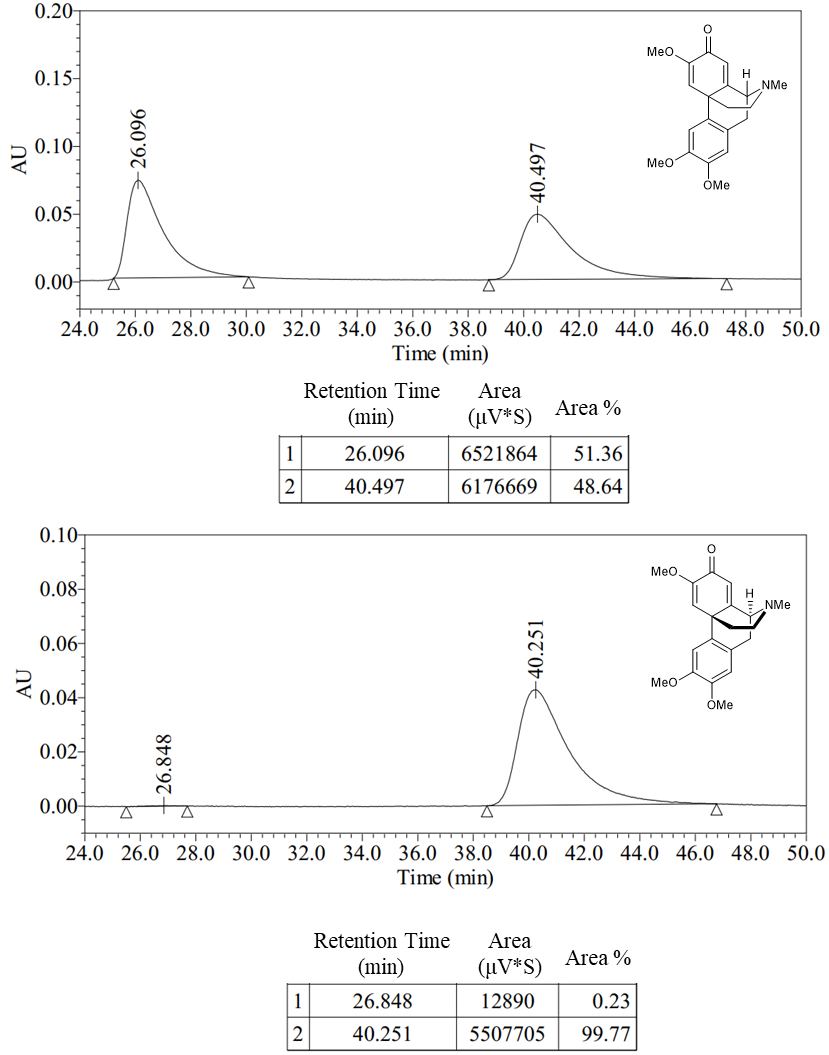


**(*S*)-amurine ((*S*)-9b)**

Yellow soild, 20.2 mg, 62% yield, >99% *ee*

**^1^H NMR (400 MHz, Chloroform-*d*)** δ 6.84 (s, 1H), 6.61 (s, 1H), 6.31 (d, *J* = 4.3 Hz, 2H), 5.93 (d, *J* = 12.6 Hz, 2H), 3.80 (s, 3H), 3.67 (d, *J* = 6.0 Hz, 1H), 3.31 (d, *J* = 17.9 Hz, 1H), 3.00 (dd, *J* = 17.9, 6.2 Hz, 1H), 2.59 – 2.57 (m, 2H), 2.46 (s, 3H), 1.97 – 1.89 (m, 1H), 1.82 (d, *J* = 12.5 Hz, 1H) ppm.

**^13^C NMR (101 MHz, Chloroform-*d*)** δ 180.9, 161.3, 151.4, 146.9, 146.8, 131.0, 129.6, 122.2, 118.8, 107.5, 105.1, 101.2, 60.7, 55.1, 45.7, 42.4, 41.7, 41.2, 32.9 ppm.

**HRMS** (ESI) for [C_19_H_19_NO_4_+H]^+^: 326.1387 (calculated), 326.1388 (found).

**HPLC**: Chiral Pak^®^ IA, n-hexane/isopropanol 50/50 flow rate = 1.0 mL/min, uv-vis λ = 280 nm, *t*_R1_ = 9.8 min (minor), *t*_R2_ = 17.2 min (major).


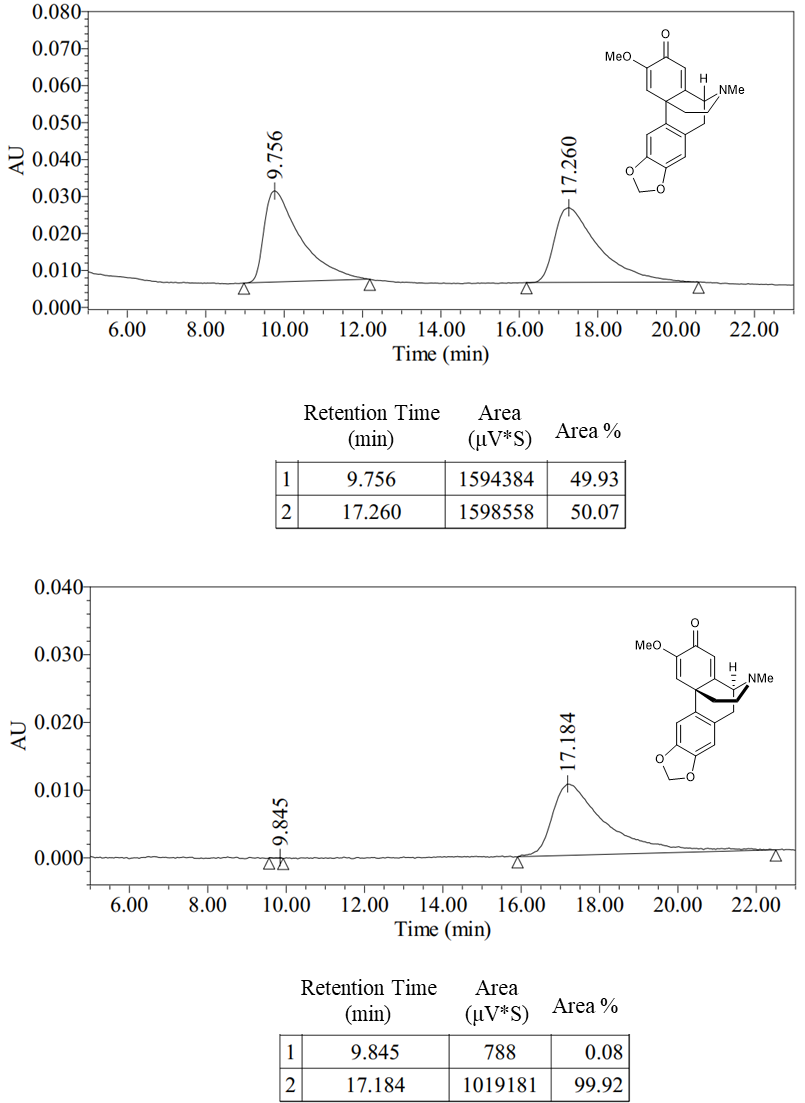


**(4b*S*,9*S*)-2,3-diethoxy-6-methoxy-11-methyl-9,10-dihydro-7H-9,4b-(epiminoethano)phenanthren-7-one ((*S*)-9c)**

Yellow soild, 18.1 mg, 49% yield, >99% *ee*

**^1^H NMR (600 MHz, Chloroform-*d*)** δ 6.85 (s, 1H), 6.64 (s, 1H), 6.34 (s, 1H), 6.33 (s, 1H), 4.11 – 4.03 (m, 4H), 3.80 (s, 3H), 3.69 (d, *J* = 6.1 Hz, 1H), 3.32 (d, *J* = 17.8 Hz, 1H), 3.03 (dd, *J* = 17.8, 6.2 Hz, 1H),2.62 – 2.52 (m, 2H), 2.46 (s, 3H), 1.93 (td, *J* = 12.1, 5.5 Hz, 1H), 1.82 (dt, *J* = 12.6, 2.5 Hz, 1H), 1.46 – 1.43 (m, 6H) ppm.

**^13^C NMR (151 MHz, Chloroform-*d*)** δ 181.0, 161.8, 151.3, 148.4, 147.5, 130.0, 129.2, 122.2, 118.9, 112.3, 111.8, 65.5, 64.5, 60.9, 55.1, 45.7, 42.3, 41.8, 41.3, 32.6, 15.0, 14.8 ppm.

**HRMS** (ESI) for [C_22_H_27_NO_4_+H]^+^: 370.2013 (calculated), 370.2005 (found).

**HPLC**: Chiral Pak^®^ IA, n-hexane/isopropanol 50/50, flow rate = 1.2 mL/min, uv-vis λ = 280 nm, *t*_R1_ = 19.0 min (minor), *t*_R2_ = 82.3 min (major).


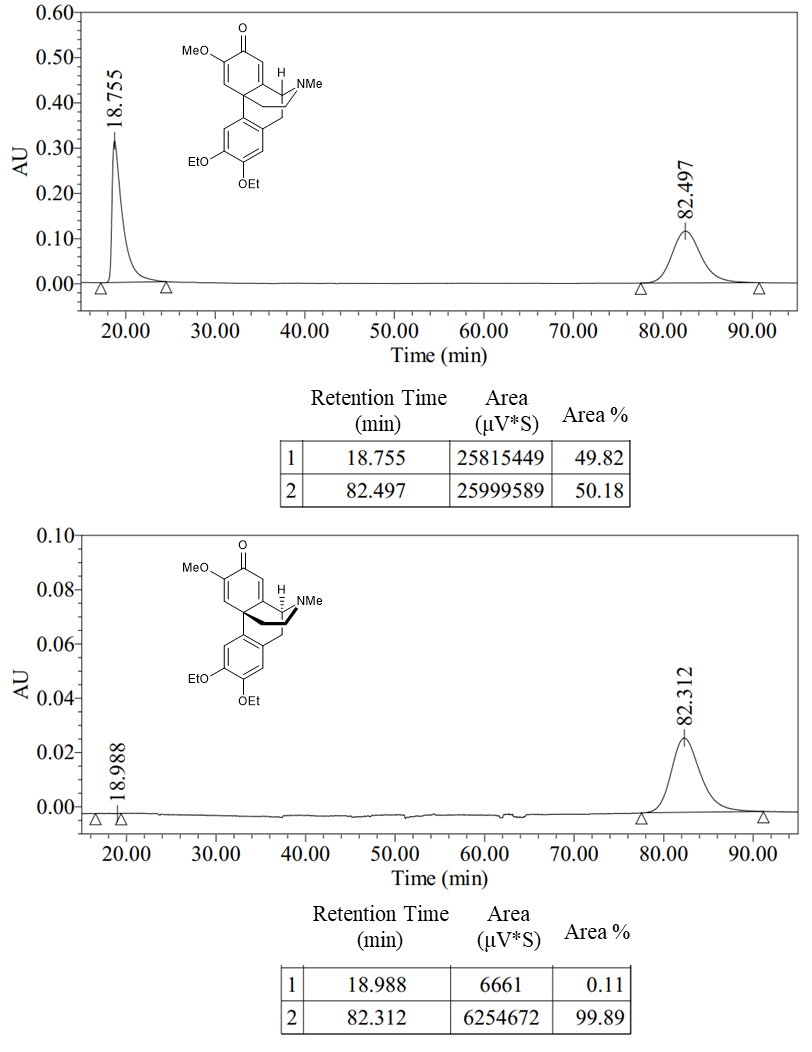


**General procedure for the synthesis of (*S*)-10**

Under argon atmosphere, (*S*)-**8** (0.3 mmol, 1 eq) and BF_3_·OEt_2_ (2.2 eq) were dissolved in hexafluoroisopropanol (HFIP) (12 mL) in an oven-dried 100 mL round bottom flask, followed by the addition of a solution of PIFA (1.2 eq) in HFIP (20 mL)^[5]^. The mixture was stirred for 45 min at room temperature, followed by neutralization with saturated aqueous Na_2_CO_3_. Then, the resulting solution was extracted with ethyl acetate for three times. The combined organic layers were washed with brine, dried over anhydrous Na_2_SO_4_, concentrated under reduced pressure, and purified by neutral alumina column chromatography using dichloromethane/methanol to obtain the crude compound. Then, the crude mixture was purified by semi-preparative HPLC. The mobile phase consisted of acetonitrile (solvent A) and water (solvent B), and a linear gradient elution method (0 min, 10% solvent A; 1-20 min, 100% solvent A; 20-25 min, 100% solvent A; 25-25.5 min 10% solvent A; 25-30 min 10% solvent A) was used to afford the desired products.

The yields of **(*S*)-10** were determined by HPLC based on their standard curves. The mobile phase consisted of solvent A (acetonitrile with 0.1% trifluoroacetic acid) and solvent B (water with 0.1% trifluoroacetic acid), and a linear gradient elution method (0 min, 10% solvent A; 1-20 min, 100% solvent A; 20-25 min, 100% solvent A; 25-25.5 min 10% solvent A; 25-30 min 10% solvent A) was used for sample analysis.

**(*S*)-glaucine ((*S*)-10a)**

Yellow soild, 56% yield, >99% *ee*

**^1^H NMR (600 MHz, Chloroform-*d*)** δ 8.10 (s, 1H), 6.79 (s, 1H), 6.59 (s, 1H), 3.94 (s, 3H), 3.91 (s, 3H), 3.89 (s, 3H), 3.65 (s, 3H), 3.19 – 3.14 (m, 1H), 3.08 – 3.01 (m, 3H), 2.69 (dd, *J* = 16.0, 3.6 Hz, 1H), 2.62 (d, *J* = 14.2 Hz, 1H), 2.57 (s, 3H), 2.53 (td, *J* = 11.9, 3.9 Hz, 1H) ppm.

**^13^C NMR (151 MHz, Chloroform-*d*)** δ 151.9, 147.9, 147.4, 144.2, 129.2, 128.8, 127.0, 126.9, 124.4, 111.5, 110.7, 110.3, 62.5, 60.2, 55.9, 55.8, 55.7, 53.3, 44.0, 34.5, 29.2 ppm.

**HRMS** (ESI) for [C_21_H_25_NO_4_+H]^+^: 356.1856 (calculated), 356.1859 (found).

**HPLC**: Chiral Pak^®^ AD-H, n-hexane/isopropanol 85/15, flow rate = 0.8 mL/min, uv-vis λ = 280 nm, *t*_R_ = 15.4 min.


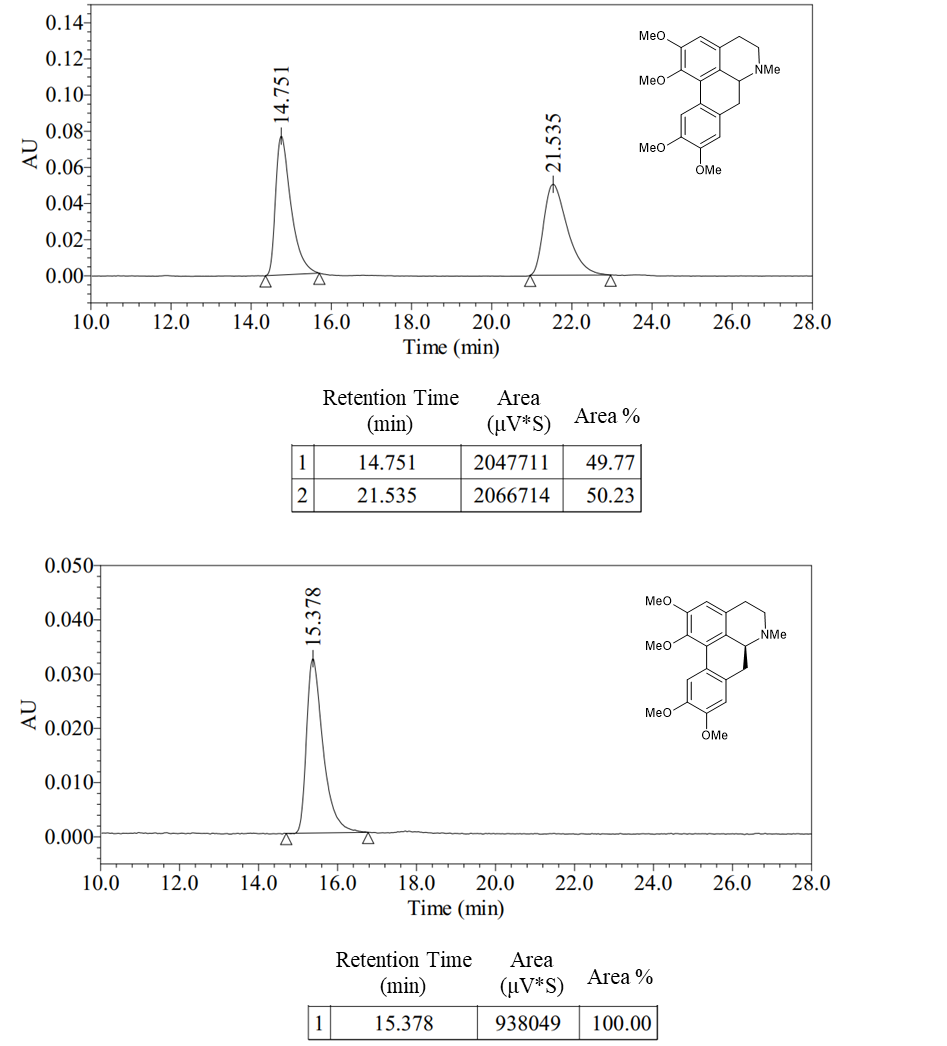


**(*S*)-nantenine ((*S*)-10b)**

Yellow soild, 50% yield, >99% *ee*

**^1^H NMR (600 MHz, Chloroform-*d*)** δ 7.93 (s, 1H), 6.76 (s, 1H), 6.59 (s, 1H), 5.98 (d, *J* = 8.6 Hz, 2H), 3.88 (s, 3H), 3.65 (s, 3H), 3.18 – 3.12 (m, 1H), 3.04 (dd, *J* = 11.4, 5.9 Hz, 1H), 3.00 – 2.96 (m, 1H), 2.56-2.50 (m, 5H) ppm.

**^13^C NMR (101 MHz, Chloroform-*d*)** δ 152.0, 146.5, 146.4, 144.6, 130.8, 128.6, 127.2, 127.0, 125.6, 110.7, 108.9, 108.3, 100.9, 62.5, 60.2, 55.9, 53.2, 43.9, 35.1, 29.2 ppm.

**HRMS** (ESI) for [C_20_H_21_NO_4_+H]^+^: 340.1543 (calculated), 340.1555 (found).

**HPLC**: Chiral Pak^®^ AD-H, n-hexane/isopropanol 95/5, flow rate = 0.6 mL/min, uv-vis λ = 280 nm, *t*_R_ = 36.9 min.


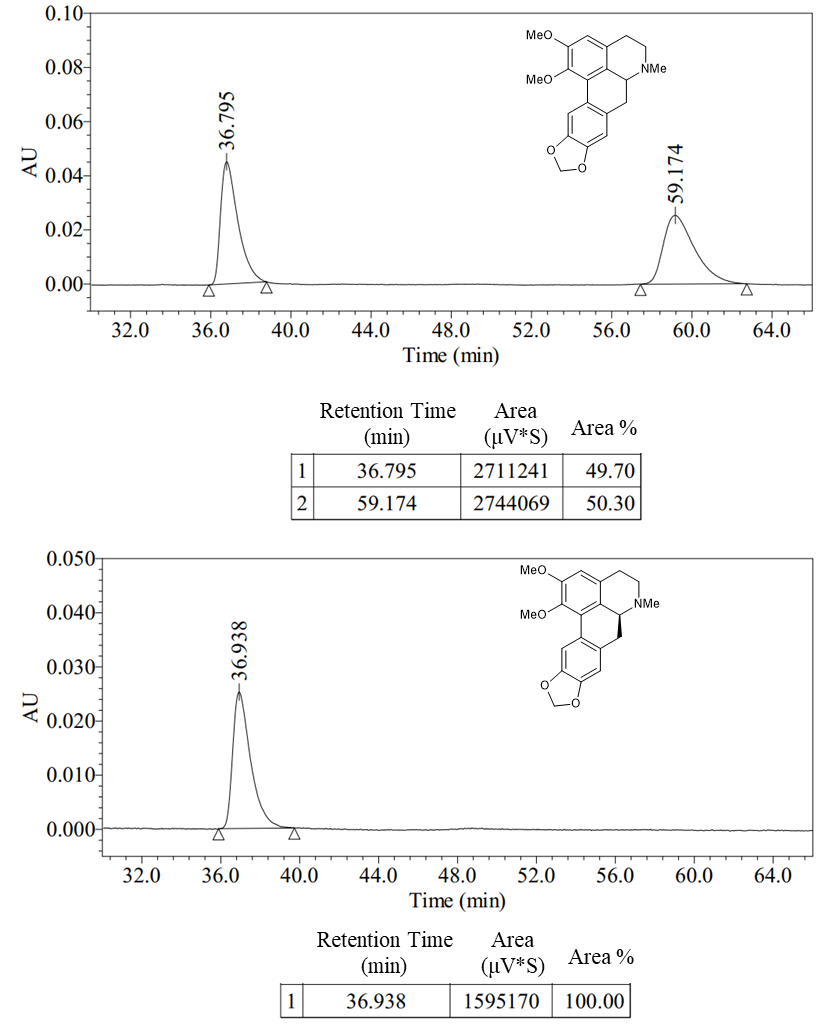


**(*S*)-9,10-diethoxy-1,2-dimethoxy-6-methyl-5,6,6a,7-tetrahydro-4H-dibenzo[de,g]quinoline ((*S*)-10c)**

Yellow soild, 45% yield, >99% *ee*

**^1^H NMR (400 MHz, Chloroform-*d*)** δ 8.07 (s, 1H), 6.77 (s, 1H), 6.58 (s, 1H), 4.19 – 4.10 (m, 4H), 3.88 (s, 3H), 3.64 (s, 3H), 3.20 – 3.11 (m, 1H), 3.07 – 2.97 (m, 3H), 2.67 (dd, *J* = 16.1, 2.5 Hz, 1H), 2.59 (d, *J* = 13.1 Hz, 1H), 2.55 (s, 3H), 2.51 (dd, 1H),1.49 (t, *J* = 3.0 Hz, 3H), 1.46 (t, *J* = 10.2 Hz, 3H) ppm.

**^13^C NMR (101 MHz, Chloroform-*d*)** δ 152.0, 147.9, 147.1, 144.4, 129.3, 128.8, 127.2, 127.1, 124.5, 113.8, 112.8, 110.4, 64.5, 64.4, 62.6, 60.2, 55.8, 53.3, 43.9, 34.5, 29.2, 15.0, 14.9 ppm.

**HRMS** (ESI) for [C_23_H_29_NO_4_+H]^+^: 384.2169 (calculated), 384.2174 (found).

**HPLC**: Chiral Pak^®^ AD-H, n-hexane/isopropanol 95/5, flow rate = 1.0 mL/min, uv-vis λ = 280 nm, *t*_R_ = 16.3 min.


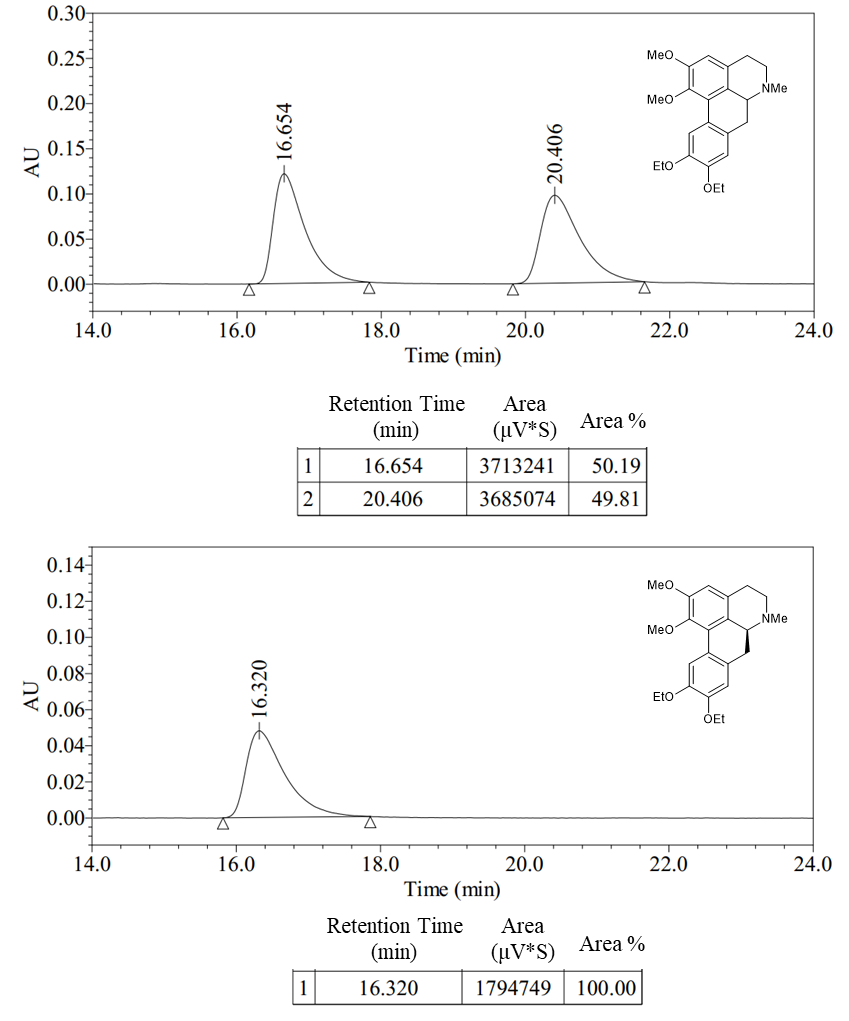


# Supplementary Tables

## **Table S1.** All strains used in this study

| **Strain** | **Description** | **Source** |
| --- | --- | --- |
| *E. coli* BL21(DE3) | *E. coli* str. B F^-^*ompT gal dcm lon hsdSB (rB^-^mB^-^) λ* (DE3 [*lacI lacUV5-T7p07 ind1 sam7 nin5*]) [*malB*^+^]_K-12_(*λ*^S^) | Novagen |
| IAA | *E. coli* BL21 (*DE3*), Δ*dkgB*, Δ*yeaE*, Δ(*yqhC*-*dkgA*), Δ*yahK*, Δ*yjgB* | Stored at Lab |
| BM1 | pRSFuet-1-*TpCAR/BsSfp-TfNCS* and pCDFDuet-1-*PsOMT2-MmSAHH-SUMO-PsN7OMT-S119T-EcMAT* were co-expressed in IAA | This study |
| M1 | pACYCuet-1-*TpCAR/BsSfp* and pCDFDuet-1-*TfNCS* were expressed in IAA | This study |
| M2 | pACYCuet-1-*TpCAR/BsSfp* and pETDuet-1-*TfNCS* were expressed in IAA | This study |
| M3 | pACYCuet-1-*TpCAR/BsSfp* and pRSFDuet-1-*TfNCS* were expressed in IAA | This study |
| M4 | pCDFDuet-1-*TpCAR/BsSfp* and pACYCDuet-1-*TfNCS* were expressed in IAA | This study |
| M5 | pCDFDuet-1-*TpCAR/BsSfp* and pETDuet-1-*TfNCS* were expressed in IAA | Stored at Lab |
| M6 | pCDFDuet-1-*TpCAR/BsSfp* and pRSFDuet-1-*TfNCS* were expressed in IAA | Stored at Lab |
| M7 | pETDuet-1-*TpCAR/BsSfp* and pACYCDuet-1-*TfNCS* were expressed in IAA | This study |
| M8 | pETDuet-1-*TpCAR/BsSfp* and pCDFDuet-1-*TfNCS* were expressed in IAA | Stored at Lab |
| M9 | pETDuet-1-*TpCAR/BsSfp* and pRSFDuet-1-*TfNCS* were expressed in IAA | Stored at Lab |
| M10 | pRSFDuet-1-*TpCAR/BsSfp* and pACYCDuet-1-*TfNCS* were expressed in IAA | This study |
| M11 | pRSFDuet-1-*TpCAR/BsSfp* and pCDFDuet-1-*TfNCS* were expressed in IAA | Stored at Lab |
| M12 | pRSFDuet-1-*TpCAR/BsSfp* and pETDuet-1-*TfNCS* were expressed in IAA | Stored at Lab |
| M13 | pACYCuet-1-*TpCAR/BsSfp-TfNCS* was expressed in IAA | This study |
| M14 | pCDFuet-1-*TpCAR/BsSfp-TfNCS* was expressed in IAA | Stored at Lab |
| M15 | pETuet-1-*TpCAR/BsSfp-TfNCS* was expressed in IAA | Stored at Lab |
| M16 | pRSFuet-1-*TpCAR/BsSfp-TfNCS* was expressed in IAA | Stored at Lab |
| S1 | pACYCuet-1-*PsOMT2*-*MmSAHH* and pCDFDuet-1- *PsN7OMT**- *EcMAT* were expressed in IAA | This study |
| S2 | pACYCuet-1-*PsOMT2*-*MmSAHH* and pETDuet-1-*PsN7OMT**- *EcMA*T were expressed in IAA | This study |
| S3 | pACYCuet-1-*PsOMT2-MmSAHH* and pRSFDuet-1-*PsN7OMT*-EcMAT* were expressed in IAA | This study |
| S4 | pCDFDuet-1-*PsOMT2-MmSAHH* and pACYCDuet-1-*PsN7OMT* -EcMAT* were expressed in IAA | This study |
| S5 | pCDFDuet-1-*PsOMT2-MmSAHH* and pETDuet-1-*PsN7OMT*-EcMAT* were expressed in IAA | This study |
| S6 | pCDFDuet-1-*PsOMT2-MmSAHH* and pRSFDuet-1-*PsN7OMT*-EcMAT* were expressed in IAA | This study |
| S7 | pETDuet-1-*PsOMT2-MmSAHH* and pACYCDuet-1- *PsN7OMT*- EcMAT* were expressed in IAA | This study |
| S8 | pETDuet-1*-PsOMT2-MmSAHH* and pCDFDuet-1-*PsN7OMT*- EcMAT* were expressed in IAA | This study |
| S9 | pETDuet-1-*PsOMT2-MmSAHH* and pRSFDuet-1-*PsN7OMT* - EcMAT* were expressed in IAA | This study |
| S10 | pRSFDuet-1-*PsOMT2-MmSAHH* and pACYCDuet-1-*PsN7OMT*-EcMAT* were expressed in IAA | This study |
| S11 | pRSFDuet-1-*PsOMT2-MmSAHH* and pCDFDuet-1-*PsN7OMT*-EcMAT* were expressed in IAA | This study |
| S12 | pRSFDuet-1-*PsOMT2-MmSAHH* and pETDuet-1-*PsN7OMT*- EcMAT* were expressed in IAA | This study |
| MA0 | pACYCDuet-1-*PsOMT2-MmSAHH-EcMAT* was expressed in IAA | This study |
| MA1 | pACYCDuet-1-*PsOMT2***-MmSAHH-EcMAT* was expressed in IAA | This study |
| MA2 | pCDFDuet-1-*PsOMT2*-MmSAHH-EcMAT* was expressed in IAA | This study |
| MA3 | pETDuet-1-*PsOMT2*-MmSAHH-EcMAT* was expressed in IAA | This study |
| MA4 | pRSFDuet-1-*PsOMT2*-MmSAHH – EcMAT* was expressed in IAA | This study |
| MB1 | pACYCDuet-1-*PsN7OMT*-MmSAHH – EcMAT* was expressed in IAA | This study |
| MB2 | pCDFDuet-1-*PsN7OMT*-MmSAHH – EcMAT* was expressed in IAA | This study |
| MB3 | pETDuet-1-*PsN7OMT*-MmSAHH-EcMAT* was expressed in IAA | This study |
| MB4 | pRSFDuet-1-*PsN7OMT*-MmSAHH-EcMAT* was expressed in IAA | This study |

## **Table S2.** All plasmids used in this study

| Plasmid | Description | Source |
| --- | --- | --- |
| pET-21b(+) | T7 promoter, pBR332 ori, Amp^R^ | Novagen |
| pET-21b(+)-*TfNCS* | pET-21b(+) carring *TfNCS* (6His in C-terminum) | Stored at Lab |
| pRSFDuet-1-*TpCAR*/*BsSfp* | pRSFDuet-1 carring *TpCAR* (6His in Nterminum) and *BsSfp* (No tag in N/C-terminum) | Stored at Lab |
| pET-21b(+)-*PsOMT2* | pET-21b(+) carring *PsOMT2* (6His in C-terminum) | This study |
| pET-21b(+)-*RnCOMT* | pET-21b(+) carring *RnCOMT* (6His in C-terminum) | Stored at Lab |
| pET-21b(+)-*GsOMT1* | pET-21b(+) carring *GsOMT1* (6His in C-terminum) | Stored at Lab |
| pET-21b(+)-*Cj6OMT* | pET-21b(+) carring *Cj6OMT* (6His in C-terminum) | This study |
| pET-21b(+)-*Tf6OMT* | pET-21b(+) carring *Tf6OMT* (6His in C-terminum) | This study |
| pET-21b(+)-*TfS9OMT* | pET-21b(+) carring *TfS9OMT* (6His in C-terminum) | This study |
| pET-21b(+)-*PsN7OMT* | pET-21b(+) carring *PsN7OMT* (6His in C-terminum) | This study |
| pET-21b(+)-*Mxsafc* | pET-21b(+) carring *Mxsafc* (6His in C-terminum) | This study |
| pACYCuet-1-*TpCAR*/*BsSfp* | pACYCDuet-1 carring *TpCAR* and *BsSfp* | This study |
| pCDFDuet-1-*TpCAR*/*BsSfp* | pCDFDuet-1 carring *TpCAR* and *BsSfp* | Stored at Lab |
| pETDuet-1-*TpCAR*/*BsSfp* | pETDuet-1 carring *TpCAR* and *BsSfp* | Stored at Lab |
| pRSFDuet-1-*TpCAR*/*BsSfp* | pRSFDuet-1 carring *TpCAR* and *BsSfp* | Stored at Lab |
| pACYCuet-1-*TfNCS* | pACYCDuet-1 carring *TfNCS* | This study |
| pCDFDuet-1-*TfNCS* | pCDFDuet-1 carring *TfNCS* | Stored at Lab |
| pETDuet-1-*TfNCS* | pETDuet-1 carring *TfNCS* | Stored at Lab |
| pRSFDuet-1-*TfNCS* | pRSFDuet-1 carring *TfNCS* | Stored at Lab |
| pACYCuet-1-*TpCAR*/*BsSfp*-*TfNCS* | pACYCDuet-1 carring *TpCAR*, *BsSfp* and *TfNCS* | This study |
| pCDFuet-1-*TpCAR*/*BsSfp*-*TfNCS* | pCDFDuet-1 carring *TpCAR*, *BsSfp* and *TfNCS* | Stored at Lab |
| pETuet-1-*TpCAR*/*BsSfp*-*TfNCS* | pETDuet-1 carring *TpCAR*, *BsSfp* and  *TfNCS* | Stored at Lab |
| pRSFuet-1-*TpCAR*/*BsSfp*-*TfNCS* | pRSFDuet-1 carring *TpCAR*, *BsSfp* and  *TfNCS* | Stored at Lab |
| pCDFDuet-1-*PsOMT2*-*MmSAHH*-*PsN7OMT*-EcMAT* | pCDFDuet-1 carring *PsOMT2*, *MmSAHH*, *PsN7OMT** and *EcMAT* | This study |
| pACYCDuet-1-*PsOMT2*-MmSAHH-EcMAT* | pACYCDuet-1 carring *PsOMT2-I113R*, *MmSAHH* and *EcMAT* | This study |
| pCDFDuet-1-*PsOMT2*-MmSAHH*-*EcMAT* | pCDFDuet-1 carring *PsOMT2-I113R I113R*, *MmSAHH* and *EcMAT* | This study |
| pETDuet-1-*PsOMT2*-MmSAHH*-*EcMAT* | pETDuet-1 carring *PsOMT2-I113R*, *MmSAHH* and *EcMAT* | This study This study |
| PRSFDuet-1-*PsOMT2**-*MmSAHH*-*EcMAT* | pRSFDuet-1 carring *PsOMT2-I113R*, *MmSAHH* and *EcMAT* | This study |
| pACYCDuet-1-*PsN7OMT**-*MmSAHH*-*EcMAT* | pACYCDuet-1 carring SUMO-*PsN7OMT-S119T*, *MmSAHH* and *EcMAT* | This study |
| pCDFDuet-1-*PsN7OMT**-*MmSAHH*-*EcMAT* | pCDFDuet-1 carring SUMO-*PsN7OMT-S119T*, *MmSAHH* and *EcMAT* | This study |
| pETDuet-1-*PsN7OMT**-*MmSAHH*-*EcMAT* | pETDuet-1 carring SUMO-*PsN7OMT*-*S119T*, *MmSAHH* and *EcMAT* | This study |
| pRSFDuet-1-*PsN7OMT**-*MmSAHH*-*EcMAT* | pRSFDuet-1 carring SUMO-*PsN7OMT*-*S119T*, *MmSAHH* and *EcMAT* | This study |

## **Table S3.** The volume of substrate-binding pocket of *Ps*OMT2

|  | **The volume of substrate-binding pocket (Å^3^)** |
| --- | --- |
| *Ps*OMT2 | 536 |
| *Ps*OMT2* | 482 |

## Table S4. Optimization of reaction conditions for the synthesis of papaverine

| **Entry** | **Conditions** | **Yield (%)** |
| --- | --- | --- |
| 1 | 0.05 M Tris-HCl (pH 9.5) /5% H_2_O_2_ | 5 |
| 2 | ACN/50% [Pd]/80 ℃/N_2_ | 45 |
| 3 | Xylene/50% [Pd]/80 ℃/N_2_ | 51 |
| 4 | Xylene/20% [Pd]/80 ℃/N_2_ | 51 |
| 5 | Xylene/10% [Pd]/80 ℃/N_2_ | 50 |
| 6 | Xylene/10% [Pd]/100 ℃/N_2_ | 70 |
| 7 | Xylene/10% [Pd]/120 ℃/N_2_ | 86 |

## Table S5. The information of the regents used in this study

| Regents | Purity (%) | Source |
| --- | --- | --- |
| Primers |  | Exsyn-bio (Wuxi, China) |
| DNA marker |  | TaKaRa |
| Protein marker |  | TaKaRa |
| PrimerSTAR MAX DNA Polymerase |  | TaKaRa |
| Phanta Flash Master Mix |  | Vazyme (Nanjing, China) |
| Tryptone |  | OXOID |
| Yeast extract |  | OXOID |
| NaCl | 99.8 | China National Pharmaceutical Group Corp (Shanghai, China) |
| Acetonitrile (HPLC) | 99.9 | Adamas-Beta (Shanghai, China) |
| 3,4-dimethoxyphenylacetic acid (1a) | 98 | Energy Chemical (Shanghai, China) |
| Dopamine hydrochloride | 98 | Energy Chemical (Shanghai, China) |
| L-Ascorbic Acid Sodium Salt | 98 | Energy Chemical (Shanghai, China) |
| Adenosine5'-(tetrahydrogen triphosphate), disodiuM salt, trihydrate (9CI) | 99 | Bide Pharmatech (Shanghai, China) |
| *L*-Methionine | 98 | Bide Pharmatech (Shanghai, China) |
| 2-(Benzo[d][1,3]dioxol-5-yl)acetic acid (1b) | 99 | Bide Pharmatech (Shanghai, China) |
| 3,4-diethoxyphenylacetic acid (1c) | 98 | Bide Pharmatech (Shanghai, China) |
| [bis(trifluoroacetoxy)iodo]benzene | 99 | Bide Pharmatech (Shanghai, China) |
| Phosphotungstic acid | AR | Bide Pharmatech (Shanghai, China) |
| Hexafluoroisopropanol | 99 | Bide Pharmatech (Shanghai, China) |
| 2 × Phanta UniFi Master Mix |  | Vazyme (Nanjing, China) |
| 2 × MultiF Seamless Assembly Mix |  | ABclonal (Wuhan, China) |

## Table S6. All primers used in this study

| **Name** | **Sequence** |
| --- | --- |
| Duet-F (MCS1) | TGCTTAAGTCGAACAGAAAGTAATCGTATTG |
| Duet-R (MCS1) | CATGGTATATCTCCTTATTAAAGTTAAACAAAATTATTTCTACAGG |
| Duet-F(MCS2) | AATTAACCTAGGCTGCTGCCACC |
| Duet-R(MCS2) | ATGTATATCTCCTTCTTATACTTGGAATTGTTATCCGC |
| *Tf*NCS-F | ACTTTAATAAGGAGATATACCATGCTGCATCACCAAGGGATTATCA |
| *Tf*NCS-R | GTGGCAGCAGCCTAGGTTAATTAGACAGTGATGATCGCAGCTTCG |
| *Tp*CAR-F | ACTTTAATAAGGAGATATACCCATCACCATCATCACCACAGCCA |
| Sfp-R | TTCTGTTCGACTTAAGCATCATAAAAGCTCTTCGTACGAGACCATTG |
| *Ps*OMT2-F | ACTTTAATAAGGAGATATACCATGGAAACCGTTTCTAAAATTGACCAAC |
| *Ps*OMT2-R | ATGTATATCTCCTTCTTAAAGTTTAGTACGGGTAGGCTTCAATAACACTTTGC |
| *Ec*MAT-F | AAGTATAAGAAGGAGATATACATATGGCAAAACACCTTTTTACGTCCG |
| *Ec*MAT-R | ATGTATATCTCCTTCTTATACTTTCACTTCAGACCGGCAGCATC |
| *Ps*N7OMT-F | GTATAAGAAGGAGATATACATATGGAGGTGGTTTCTCAGATCGAC |
| *Ps*N7OMT-F | GGCAGCAGCCTAGGTTAATTATCAATAAACTTCAATGATTGACTGTGCAGCG |
| SUMO-F | TAAGAAGGAGATATACATATGTCGGACTCAGAAGTCAATCAAGAAGC |
| SUMO-R | TCGATCTGAGAAACCACCTCGCCTCCAATCTGTTCGCGG |
| MBP-F | CTTTAAGAAGGAGATATACATATGAAAATCGAAGAAGGTAAACTGGTAATCTGGA |
| MBP-R | GTCGATCTGAGAAACCACCTCAGTCTGCGCGTCTTTCAGG |
| GST-F | TAAGAAGGAGATATACATATGTCCCCTATACTAGGTTATTGGAAAATTAAGGGCC |
| GST-F | GTCGATCTGAGAAACCACCTCTTTTGGAGGATGGTCGCCACC |
| pET21b-F | ATGTATATCTCCTTCTTAAAGTTAAACAAAATTATTTCTAGAGGGG |
| pET21b-R | CTCGAGCACCACCACCAC |
| *Ps*N7OMT-S119A-F | CAGTCGATGGTACCC**GCT**GTATTGGGTATCATTGACGAG |
| *Ps*N7OMT-S119H-F | CAGTCGATGGTACCC**CAT**GTATTGGGTatCATTGACGAGG |
| *Ps*N7OMT-S119C-F | CAGTCGATGGTACCC**TGT**GTATTGGGTatCATTGACGAGG |
| *Ps*N7OMT-S119Q-F | CAGTCGATGGTACCC**CAG**GTATTGGGTatCATTGACGAGG |
| *Ps*N7OMT-S119T-F | CAGTCGATGGTACCC**ACG**GTATTGGGTatCATTGACGAGG |
| *Ps*N7OMT-S119K-F | CAGTCGATGGTACCC**AAA**GTATTGGGTatCATTGACGAGG |
| *Ps*N7OMT-S119-R | GGGTACCATCGACTGTTGGGAG |
| *Ps*N7OMT-D175A-R | GTCCGCT**AGC**GAAGGCCATCGCG |
| *Ps*N7OMT-D175A-F | CTTC**GCT**AGCGGACTGGTGACATCGCA |
| *Ps*N7OMT-D264A-R | CCA**AGC**GTGAAGAATGTTTTTCATAAAGATAGCATCCG |
| *Ps*N7OMT-D264A-F | CTTCAC**GCT**TGGAATGACGACGAATGTATCCAG |
| *Ps*N7OMT-M292A-F | GTCGAA**GCA**GTGTTAGATGAAGATTCATTCCACCCTTACTC |
| *Ps*N7OMT-M292A-R | CTAACAC**TGC**TTCGACCATGATTAACTTACC |
| *Ps*N7OMT-S309A-F | TGACG**GCC**GATATCGACATGATGGTTAACAATGGCGG |
| *Ps*N7OMT-S309-R | CGTCAAGCGCAGCTTGGAGT |
| *Ps*N7OMT-S309K-F | CAAGCTGCGCTTGACG**AAG**GATATCGACATGATGGTTAACAATGGCGG |
| *Ps*N7OMT-S309H-F | CAAGCTGCGCTTGACG**CAT**GATATCGACATGATGGTTAACAATGGCGG |
| *Ps*N7OMT-M313A-F | ATCGAC**GCA**ATGGTTAACAATGGCGGAAAGGAAC |
| *Ps*N7OMT-M313A-R | TTAACCAT**TGC**GTCGATATCGGCCGTCAA |
| *Ps*N7OMT-N317A-F | GGTTAAC**GCT**GGCGGAAAGGAACGCACG |
| *Ps*N7OMT-N317A-R | TTCCGCC**AGC**GTTAACCATTGCGT |
| *Ps*N7OMT-Q351A-F | GCTGCA**GCA**TCAATCATTGAAGTTTATCTCGAGCACCAC |
| *Ps*N7OMT-Q351A-R | ATGATTGA**TGC**TGCAGCGAAACCG |
| *Ps*OMT2-S109A-F | AAATCTATGGTGGAT**GCC**ATTTTGTGCATCAATGACAAGGATTTCTTGG |
| *Ps*OMT2-S109-R | GCACAAAATAGAATCCACCATAGATTTCTCC |
| *Ps*OMT2-I113A-F | ATTCTATTTTGTGC**GCC**AATGACAAGGATTTCTTGGCCCCT |
| *Ps*OMT2-I113D-F | ATTCTATTTTGTGC**GAC**AATGACAAGGATTTCTTGGCCCCT |
| *Ps*OMT2-I113F-F | ATTCTATTTTGTGC**TTC**AATGACAAGGATTTCTTGGCCCCT |
| *Ps*OMT2-I113H-F | ATTCTATTTTGTGC**CAC**AATGACAAGGATTTCTTGGCCCCT |
| *Ps*OMT2-I113K-F | ATTCTATTTTGTGC**AAA**AATGACAAGGATTTCTTGGCCCCT |
| *Ps*OMT2-I113R-F | ATTCTATTTTGTGC**GTC**AATGACAAGGATTTCTTGGCCCCT |
| *Ps*OMT2-I113V-F | ATTCTATTTTGTGC**GTG**AATGACAAGGATTTCTTGGCCCCT |
| *Ps*OMT2-I113W-F | ATTCTATTTTGTGC**TGG**AATGACAAGGATTTCTTGGCCCCT |
| *Ps*OMT2-I113-R | GCACAAAATAGAATCCACCATAGATTTCTCC |
| *Ps*OMT2-F158A-F | TGAGAAGAACCAATT**GCA**CAATGCGGCAATGGCCTG |
| *Ps*OMT2-F158-R | AATTGGTTCTTCTCAGGATTTTCACTCAT |
| *Ps*OMT2-M162-R | TGCCGCATTGAACAATTGGTTCT |
| *Ps*OMT2-M162A-F | AACCAATTGTTCAATGCG**GCA**GCGGCCTGCGATACTCGTCTGG |
| *Ps*OMT2-D165E-F | GCGGCAATGGCCTGC**GAA**ACTCGTCTGGTGACAAGTGC |
| *Ps*OMT2-D165H-F | GCGGCAATGGCCTGC**CAC**ACTCGTCTGGTGACAAGTGC |
| *Ps*OMT2-D165R-F | GCGGCAATGGCCTGC**CGT**ACTCGTCTGGTGACAAGTGC |
| *Ps*OMT2-D165W-F | GCGGCAATGGCCTGC**TGG**ACTCGTCTGGTGACAAGTGC |
| *Ps*OMT2-D165K-F | GCGGCAATGGCCTGC**AAA**ACTCGTCTGGTGACAAGTGC |
| *Ps*OMT2-D165R | GCAGGCCATTGCCGCATTG |
| *Ps*OMT2-L299A-F | AAAATTCGTCTTACC**GCG**GACTTAGATATGATGTTGAACACTGGAGGG |
| *Ps*OMT2-L299-R | GGTAAGACGAATTTTCGCGTATGGATG |
| *Ps*OMT2-D302-R | TAAGTCAAGGGTAAGACGAATTTTCGCGTAT |
| *Ps*OMT2-D302E-F | CTTACCCTTGACTTA**GAA**ATGATGTTGAACACTGGAGGGAAAGAAC |
| *Ps*OMT2-M303A-F | ACCCTTGACTTAGAT**GCG**ATGTTGAACACTGGAGGGAAAGAAC |
| *Ps*OMT2-M303-R | ATCTAAGTCAAGGGTAAGACGAATTTTCGC |
| *Ps*OMT2-Q338N-F | AAATTAGCGCGGTG**AAC**AGTGTTATTGAAGCCTACCCGTACC |
| *Ps*OMT2-Q338-R | CACCGCGCTAATTTGCGTAAC |

# Supplementary Figures


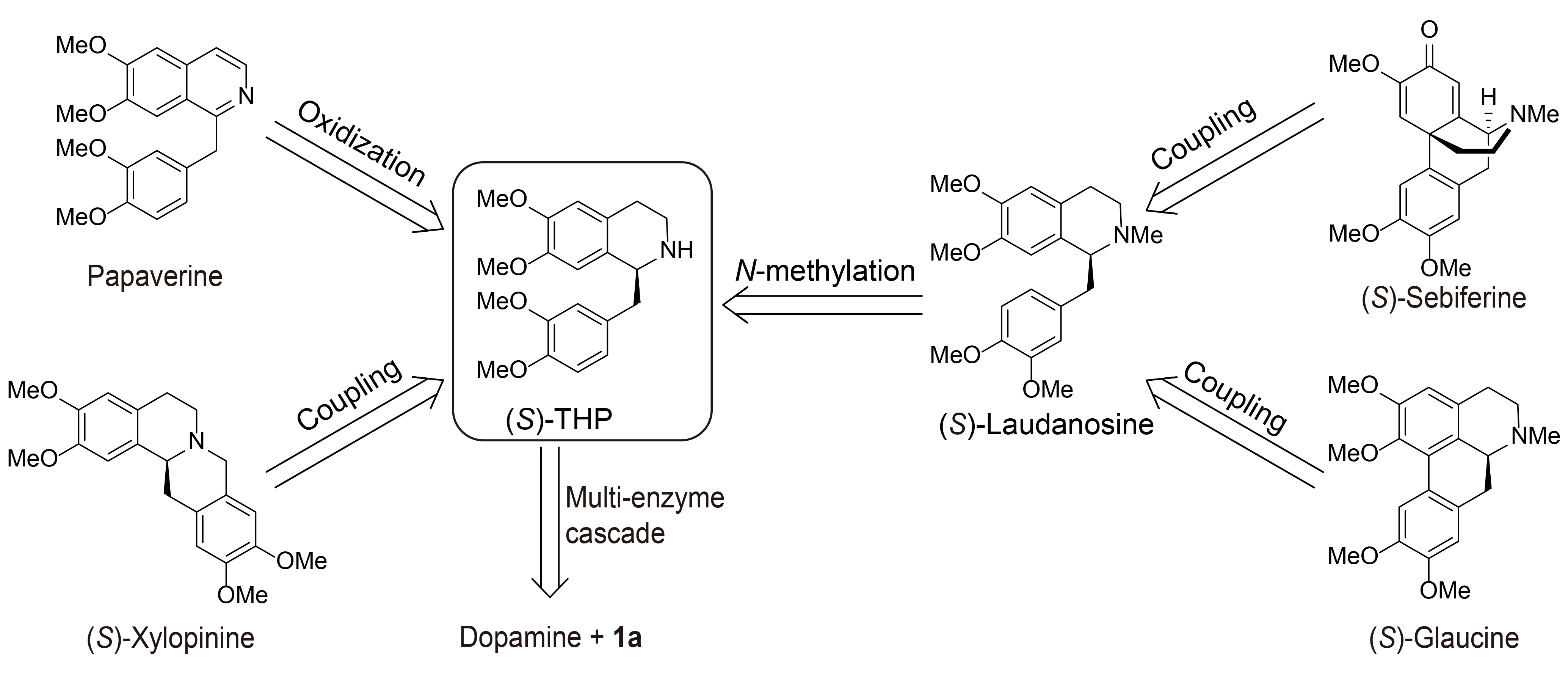


## Figure S1. Retrosynthetic analysis of natural benzylisoquinoline alkaloids.


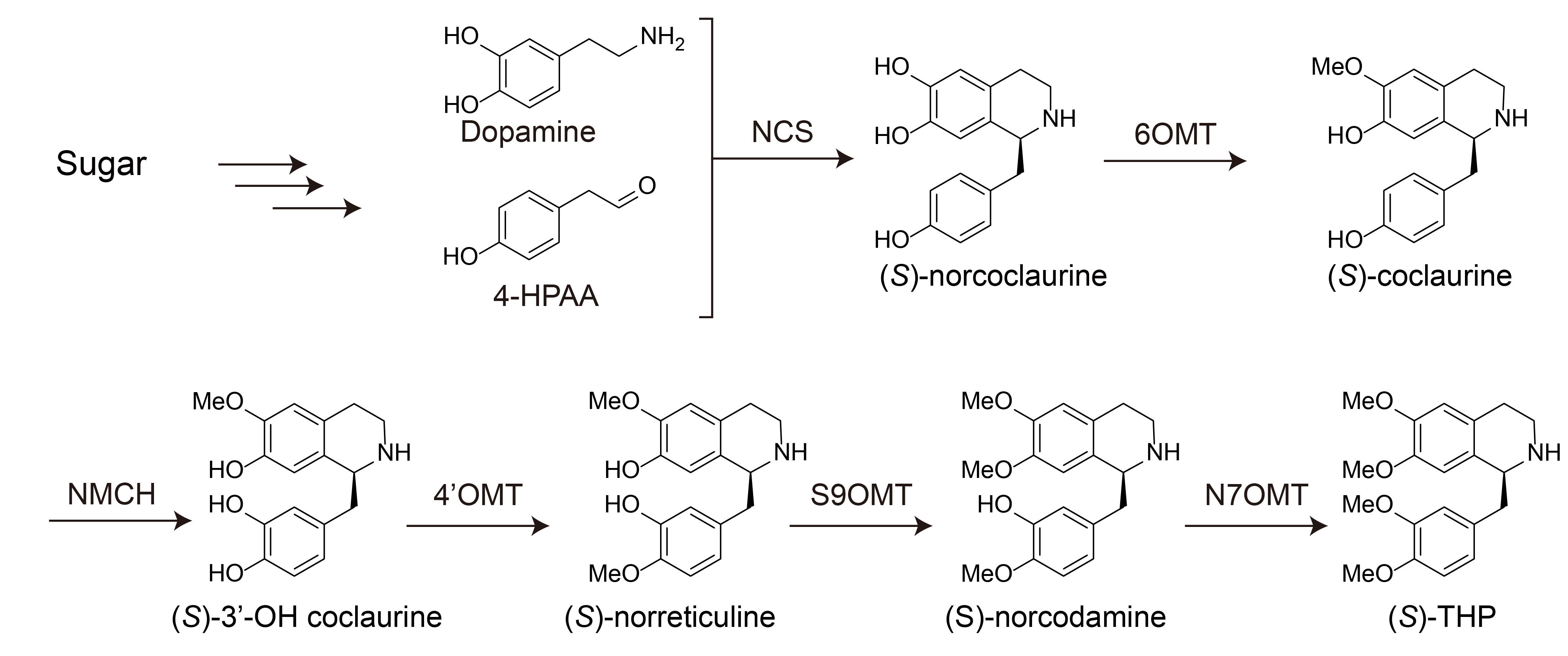


Figure S2. Biosynthetic pathway for (*S*)-THP in *Saccharomyces cerevisiae*. NCS, norcoclaurine synthase. 6OMT, norcoclaurine 6-*O*-methyl transferase. CNMT: coclaurine *N*-methyl transferase. NMCH, *N*-methylcoclaurine hydroxylase. LdM, laudanosine demethylase. CocH, coclaurine hydroxylase. 4’OMT, *N*-methylcoclaurine and coclaurine 4*-O*-methyl transferase. 7OMT, norreticuline and reticuline 7-*O*-methyl transferase. 3’OMT, norlaudanine and laudanine 3-*O*-methyl transferase. 4-HPAA, 4-hydroxyphenylacetaldehyde.

**
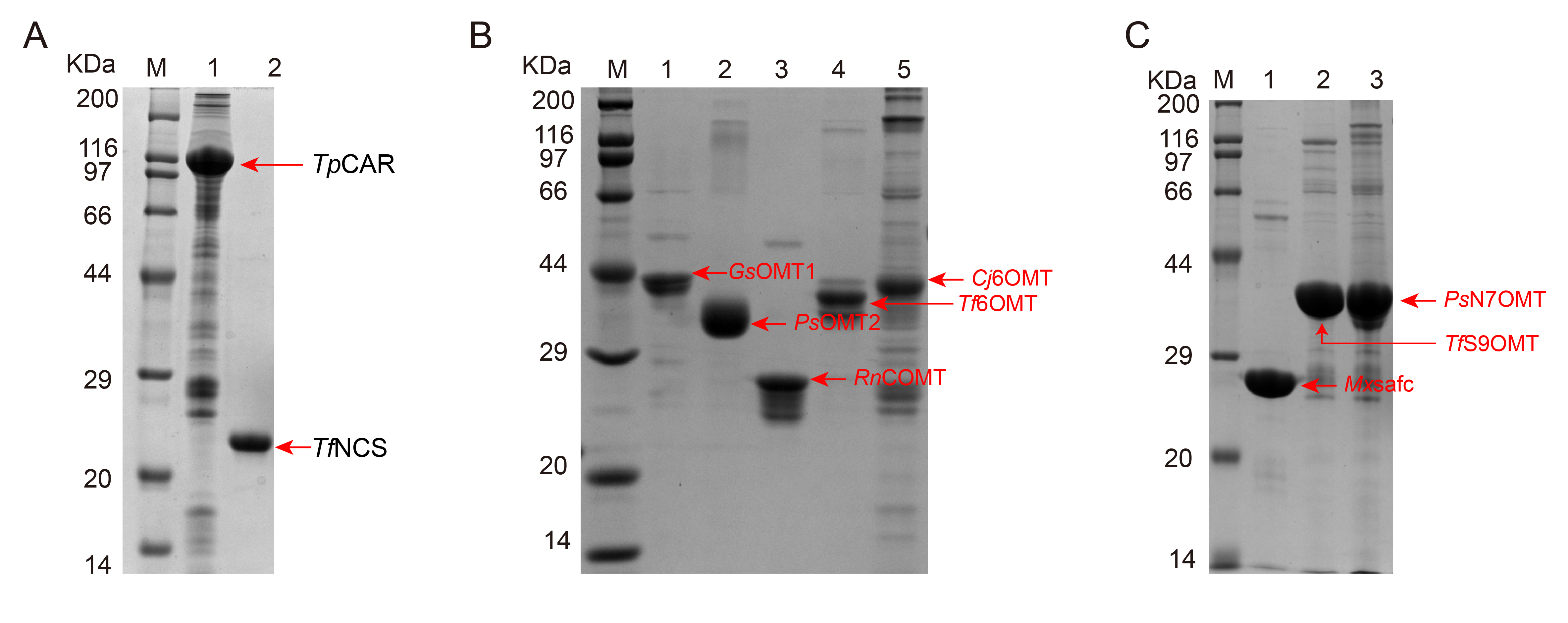
**

Figure S3. SDS-PAGE analysis of purified proteins**.** A) SDS-PAGE analysis of purified *Tp*CAR and *Tf*NCS. M: marker; lane 1: holo-*Tp*CAR; lane 2: *Tf*NCS. B) SDS-PAGE analysis of purified five 6-*O*-methyltransferases. M: marker; lane 1: *Gs*OMT1; lane 2: *Ps*OMT2; lane 3: *Rn*COMT; lane 4: *Tf*6OMT; lane 5*Cj*NCS. C) SDS-PAGE analysis of purified three 7-*O*-methyltransferases. M: marker; lane 1: *Mx*safc; lane 2: *Tf*S9OMT; lane 3: *Ps*N7OMT.

**
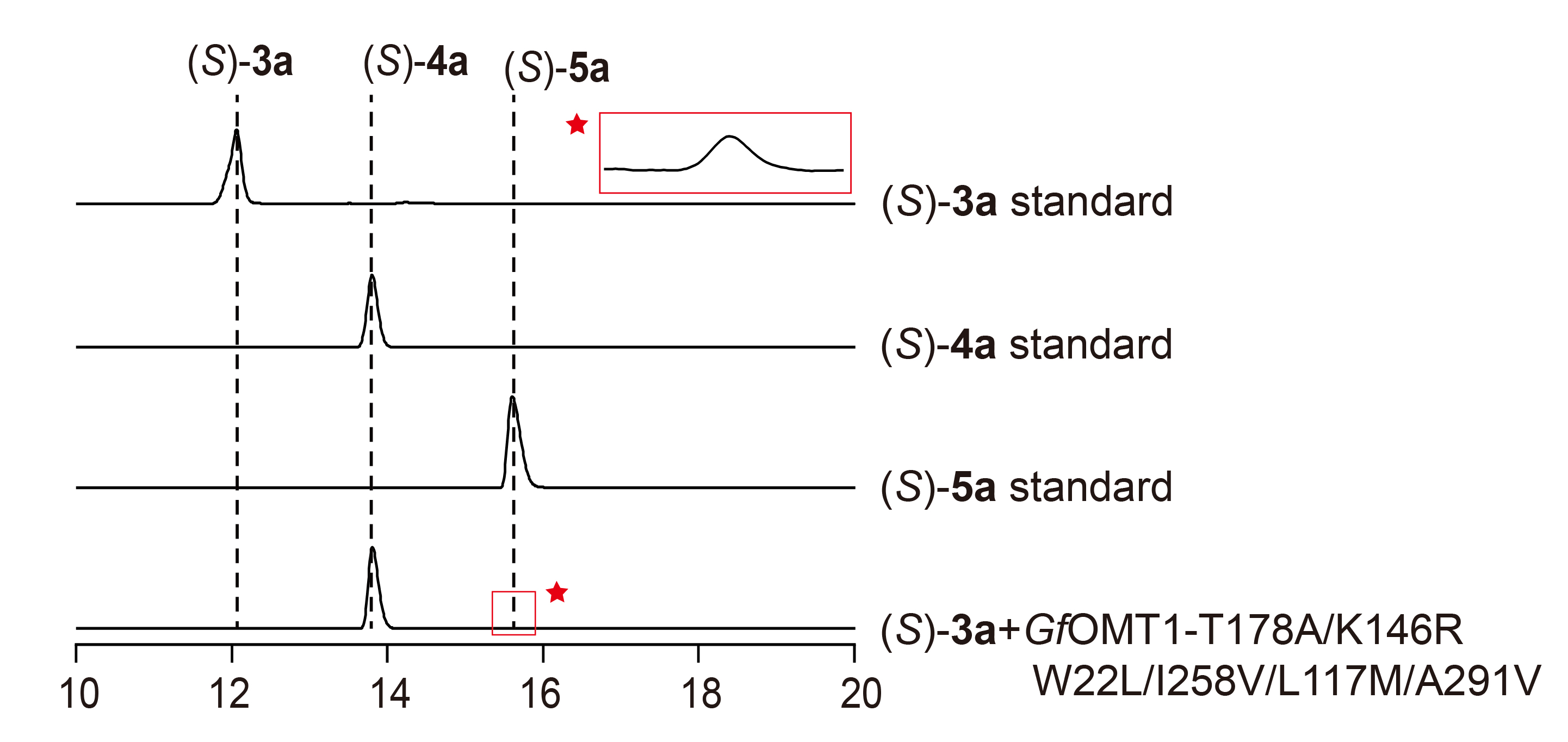
**

Figure S4. Screening of engineered methyltransferases *Gf*OMT1-T178A/K146R/W22L/I258V/L117M/A291V in vitro. The products (*S*)-5a was observed when enlarging the spectrum highlighted in red rectangle.


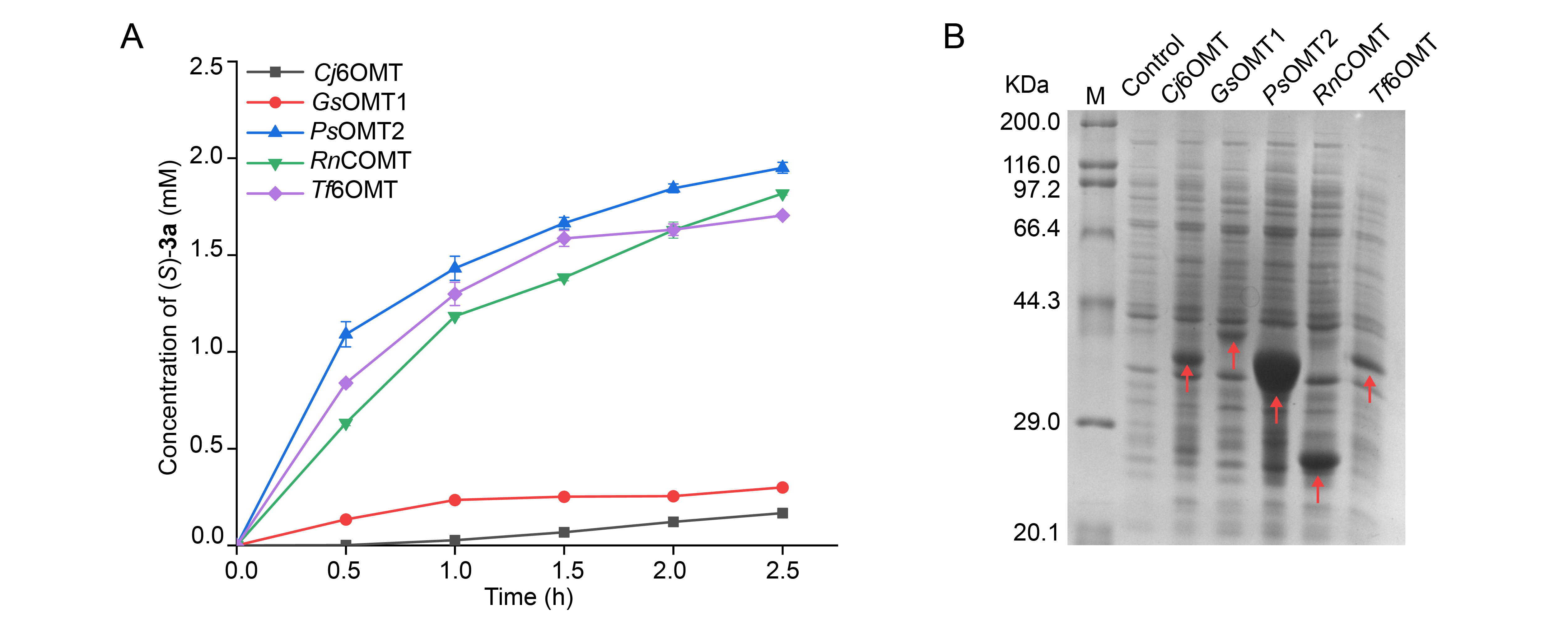


Figure S5. Screening of five different 6-*O*-methyltransferase for transforming (*S*)-3a to (*S*)-4a. A) Time course for the production of (*S*)-**3a** catalyzed by five 6-*O*-methyltransferase. All tests were performed with 2 mM (*S*)-**3a**. B) SDS-PAGE analysis for expression levels of five 6-*O*-methyltransferase in *E. coli* BL21 (*DE3*). M: marker; lane 1: no IPTG induction; lane 2: *Cj*NCS; lane 3: *Gs*OMT1; lane 4: *Ps*OMT2; lane 5: *Rn*COMT; lane 6: *Tf*6OMT. All proteins are marked with red arrows. All data are presented as mean values of three independent experiments and the error bars indicate ±sd.


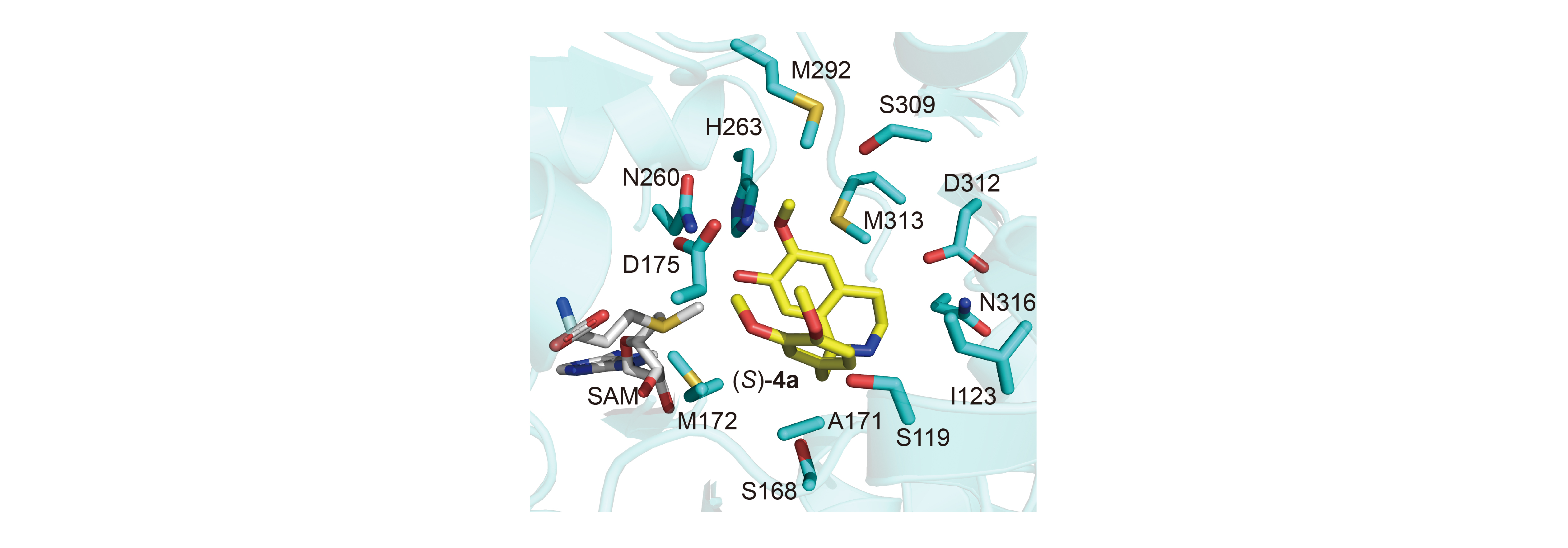


Figure S6. Molecular docking of *Ps*N7OMT with (*S*)-4a. The substrate (*S*)-**4a** is colored in yellow, SAM is colored in gray, and the selected residues are colored in cyan. Oxygen, nitrogen and sulfur atom are shown in red, blue and yellow, respectively.


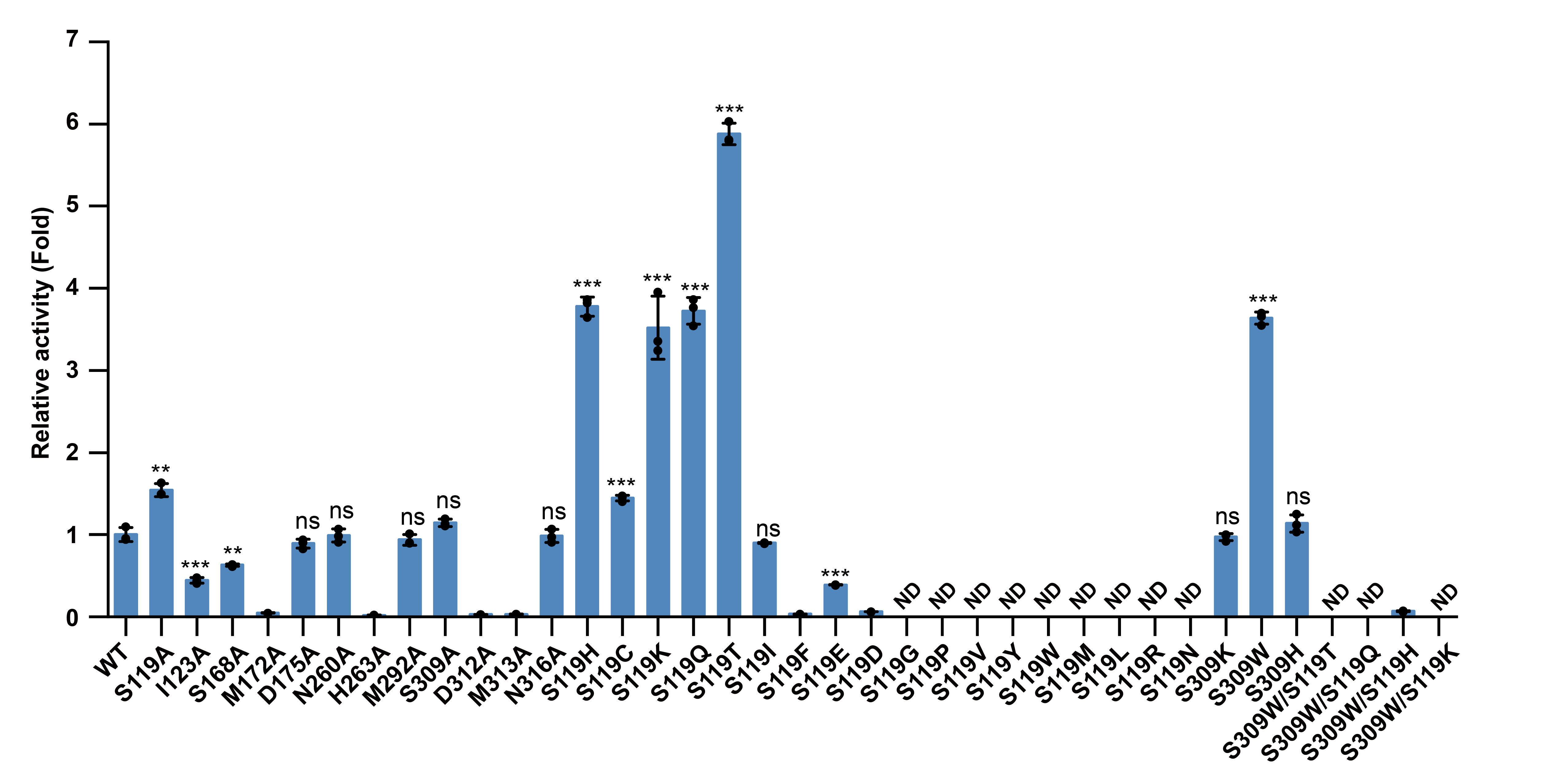


Figure S7. Relative catalytic activity of mutants of *Ps*N7OMT. ND refers to not detected. All data are presented as mean values of three independent experiments and the error bars indicate ±sd. Two-tailed Student’s t-test was conducted for analyzing the significant difference (P > 0.05, ns, no significance, **p* < 0.05, ***p* < 0.01, ****p* < 0.001).


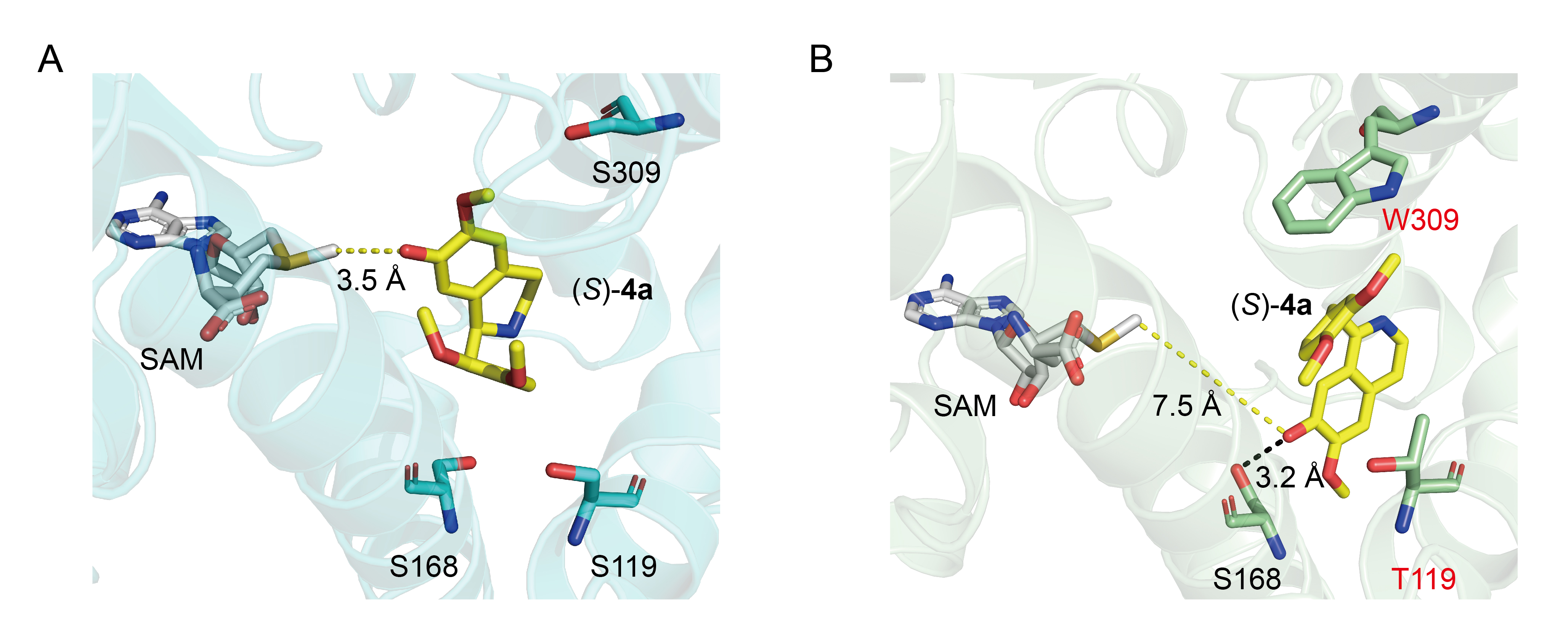


Figure S8. Structural analysis of *Ps*N7OMT and *Ps*N7OMT-S119T/S309W with the substrate (*S*)-4a. A) Structural model of PsN7OMT binding with (*S*)-4a. B) Structural model of PsN7OMT-S119T/S309W binding with (*S*)-4a. (*S*)-4a is colored in yellow. SAM is colored in grey. *Ps*N7OMT and selected residues are colored in cyan. *Ps*N7OMT-S119T/S309W and selected residues are colored in pale green. Oxygen, nitrogen and sulfur atom are colored in red, blue and yellow, respectively.


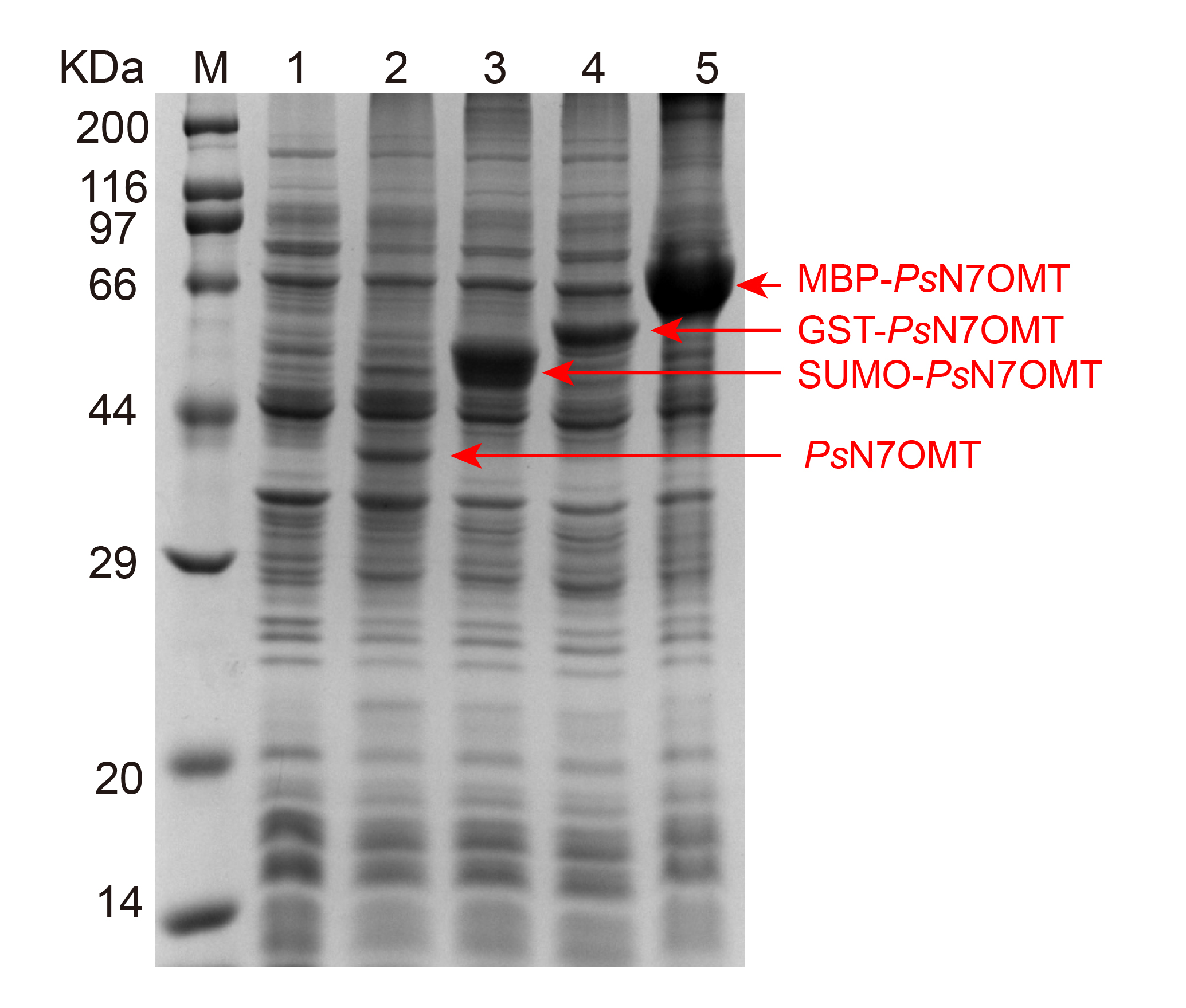


Figure S9. SDS-PAGE analysis for proteins expression of *Ps*N7OMT with different tags. M: marker; lane 1: no IPTG induction; lane 2, the supernatant of cell lysates expressing *Ps*N7OMT-S119T induced by IPTG; lane 3, the supernatant of cell lysates expressing SUMO-*Ps*N7OMT-S119T induced by IPTG; lane 4, the supernatant of cell lysates expressing GST-*Ps*N7OMT-S119T induced by IPTG; lane 5, the supernatant of cell lysates expressing MBP-*Ps*N7OMT induced by IPTG.


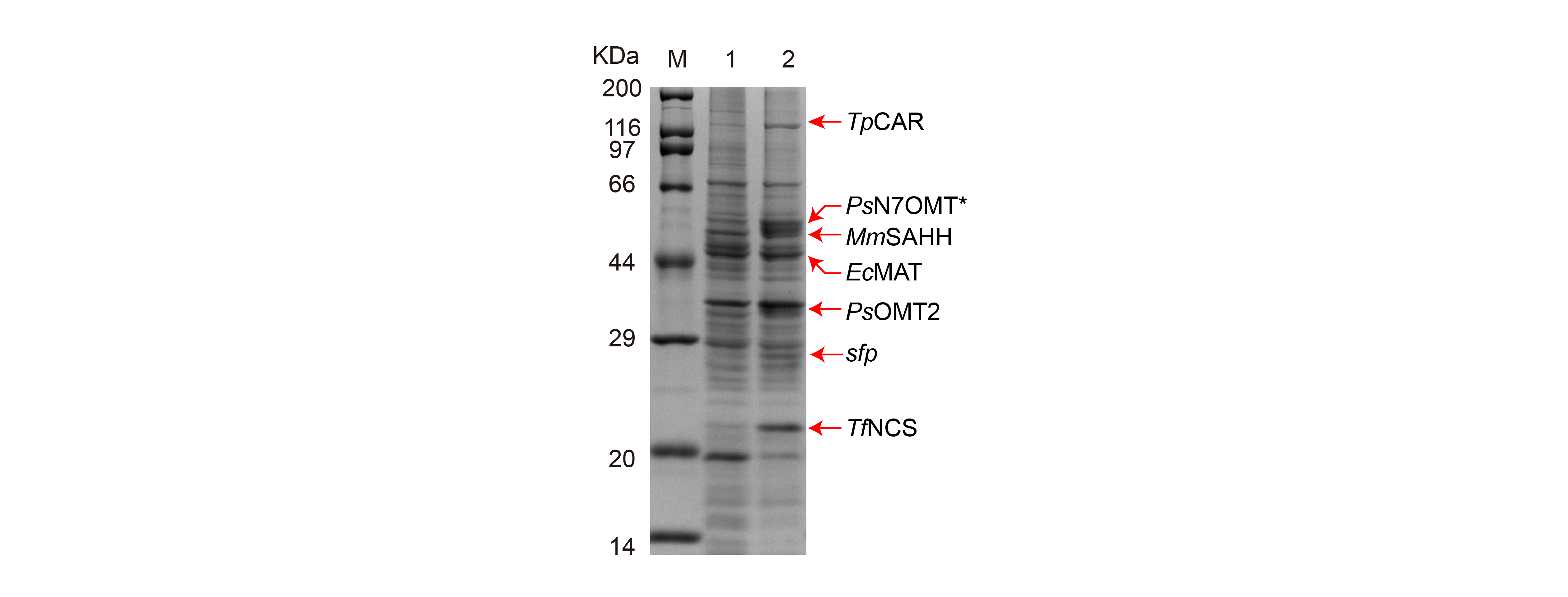


Figure S10. SDS-PAGE analysis for proteins expression of proteins in strain BM1. M: marker; lane 1: no IPTG induction; lane 2: the cell lysates supernatant of strain BM1 induced by IPTG.

**
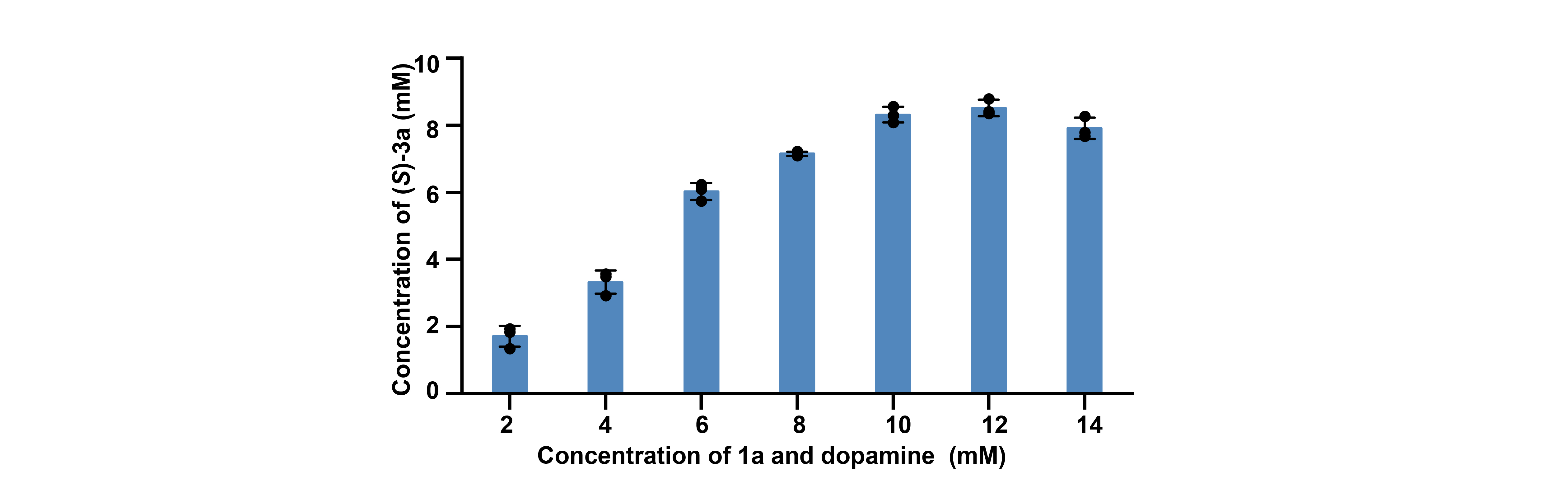
**

Figure S11. Effect of substrate concentrations on the titer of (*S*)-3a. All data are presented as mean values of three independent experiments and the error bars indicate ±sd.

**
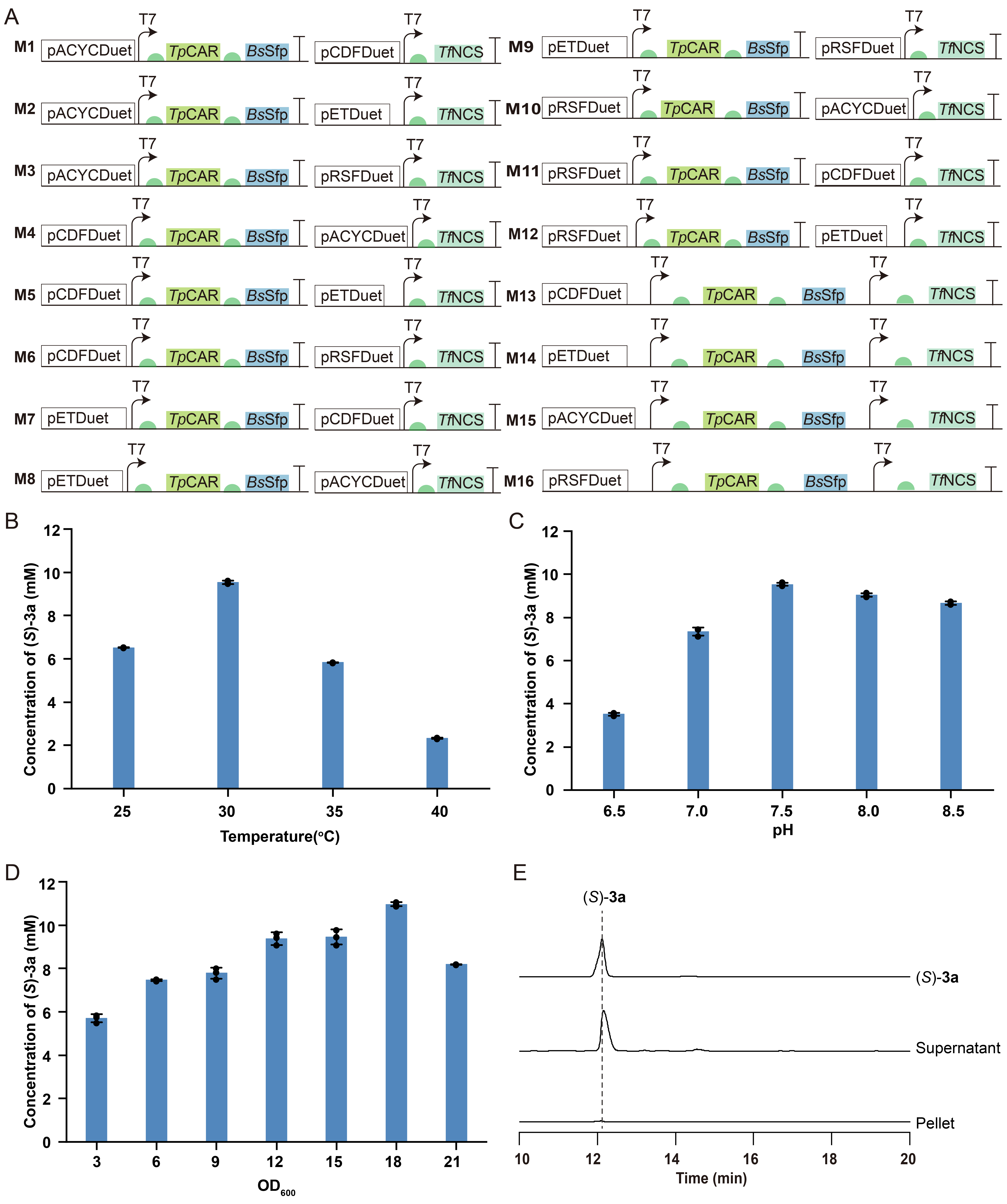
**

Figure S12. Optimization of the whole-cell reaction conditions of the BIA module. A) Construction of sixteen engineered strains with different plasmids expressing *Tp*CAR, *Bs*Sfp and *Tf*NCS. The green semicircle indicates the ribosome binding site. B) Optimization of temperature for the biosynthesis of (*S*)-**3a**. C) Optimization of pH for the biosynthesis of (*S*)-**3a**. D) Optimization of strain concentration for the biosynthesis of (*S*)-**3a**. E) Distribution analysis of (*S*)-**3a** in the supernatant and the pellet. All data are presented as mean values of three independent experiments and the error bars indicate ±sd.


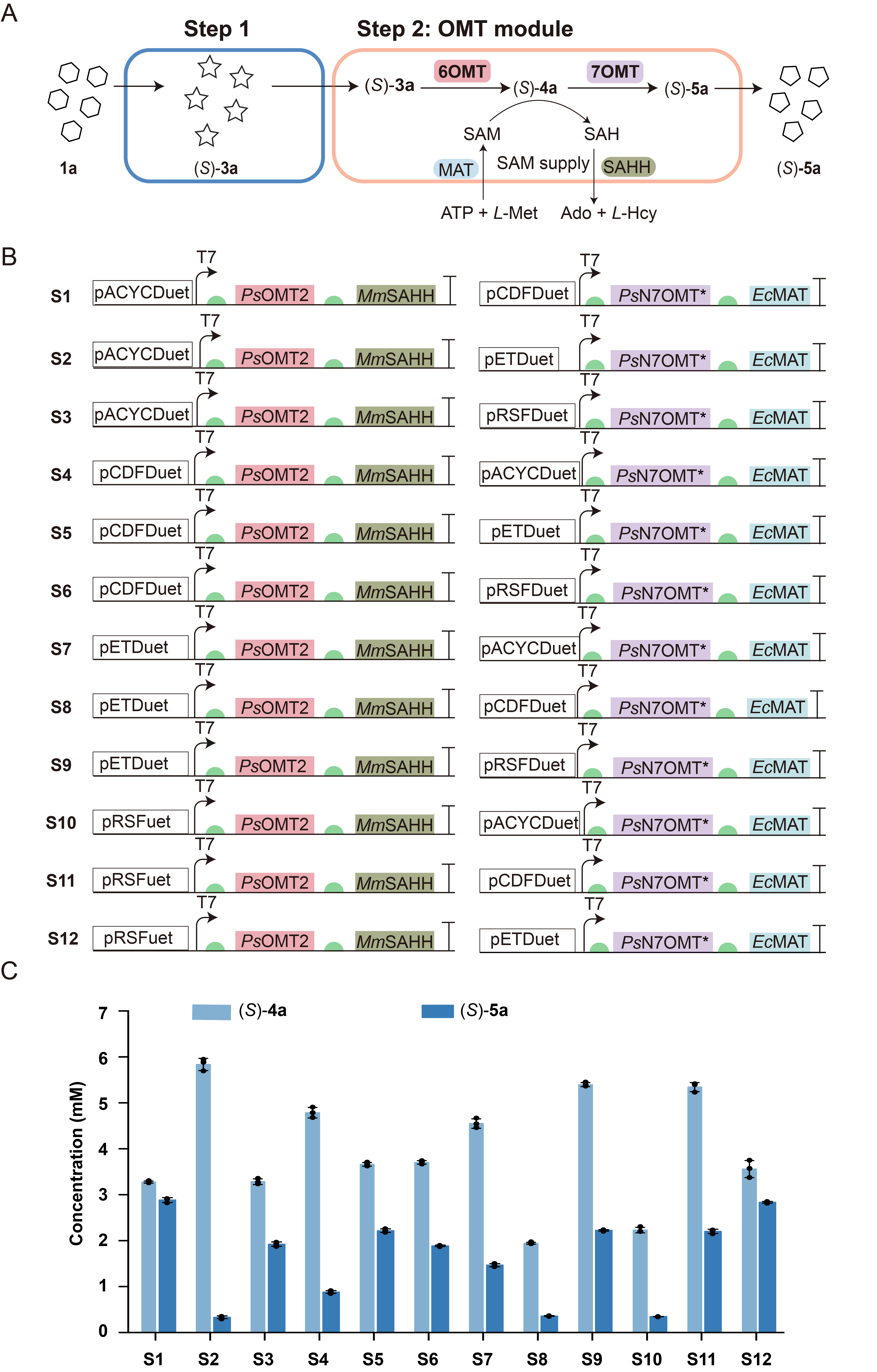


Figure S13. Design of OMT module for the biosynthesis of (*S*)-**5a** from (*S*)-**3a** via one-pot two-step strategy. The biosynthesis of (*S*)-**5a** is catalyzed by 6OMT and 7OMT from (*S*)-**3a**. A) Illustration of the designed whole-cell catalysis for the preparation of (*S*)-**5a** from (*S*)-**3a.** B) Construction of sixteen engineered strains with different plasmids expressing *Ps*6OMT and *Ps*N7OMT. C) The titer of (*S*)-**4a** and (*S*)-**5a** in engineered strains S1-S12. All data are presented as mean values of three independent experiments and the error bars indicate ±sd.


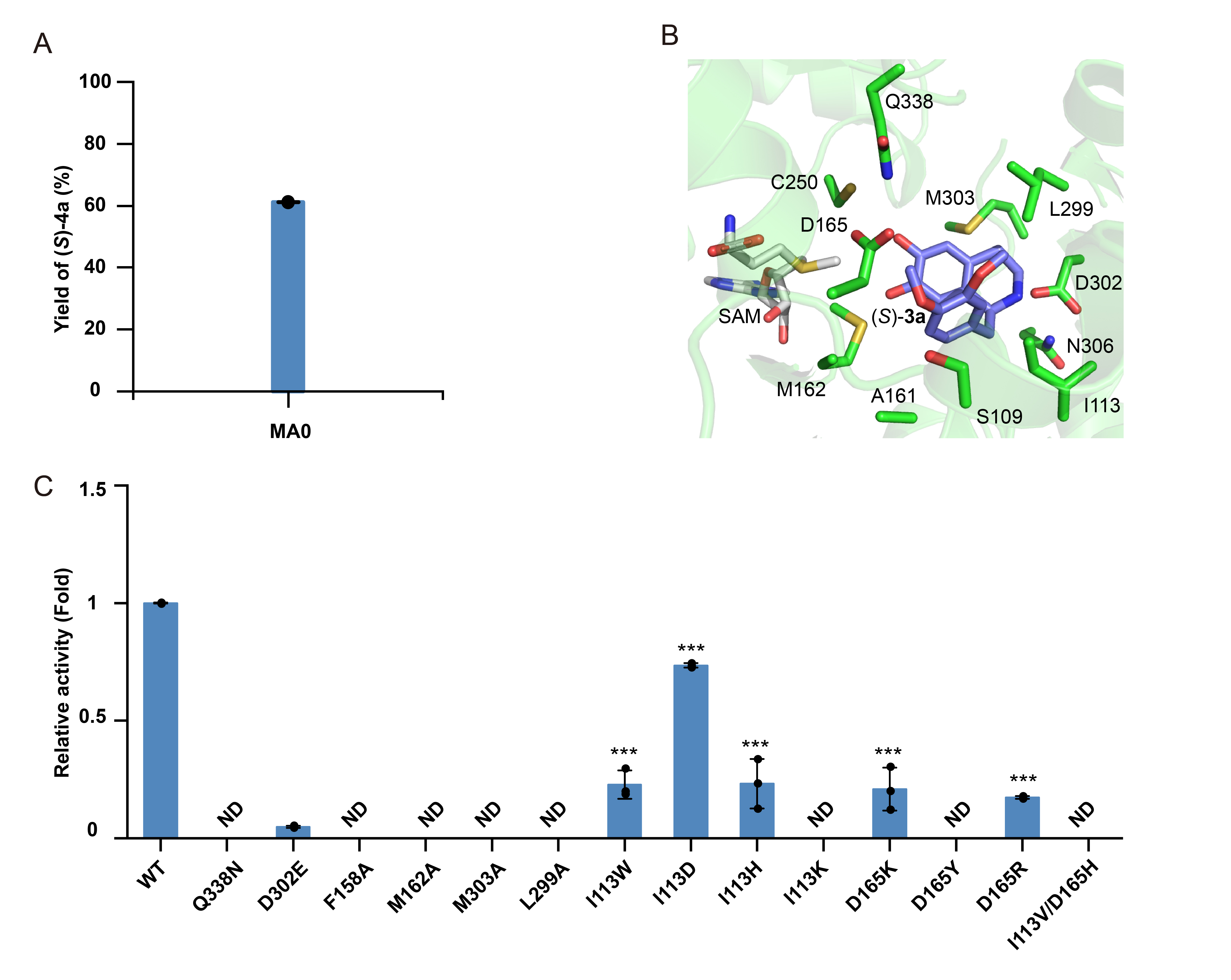


Figure S14. Structure-guided engineering to improve the enzymatic activity of *Ps*OMT2. A) Yield of (*S*)-**4a** catalyzed by engineering strain S1. It corresponds to a theoretical yield of (*S*)-**3a**. B) Molecular docking of *Ps*OMT2 with (*S*)-**3a**. The substrate (*S*)-**3a** is colored in purple. SAM is colored in gray, and the selected residues are colored in green. Oxygen, nitrogen and sulfur atom are shown in red, blue and yellow, respectively. C) Relative catalytic activity of mutants of *Ps*OMT2. All data are presented as mean values of three independent experiments and the error bars indicate ±sd. Two-tailed Student’s t-test was conducted for analyzing the significant difference (*p* > 0.05, ns, no significance, **p* < 0.05, ***p* < 0.01, ****p* < 0.001).
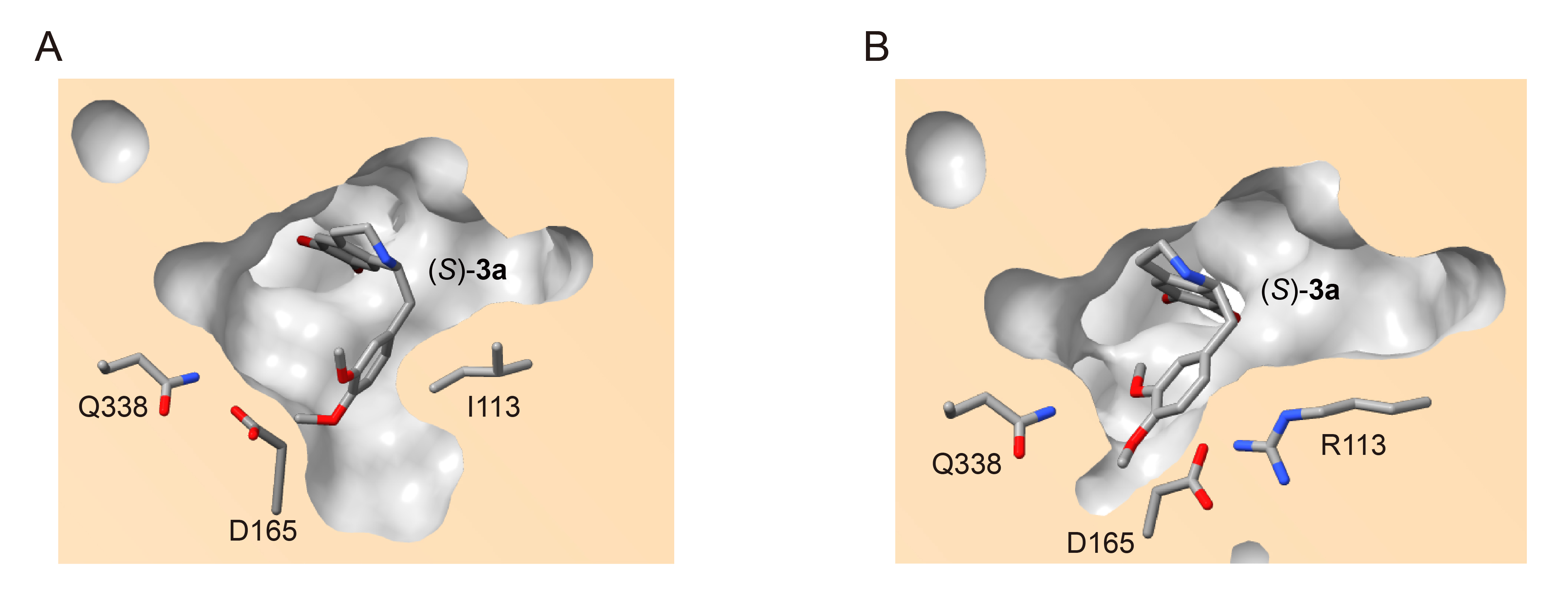


Figure S15. Cross-section of the substrate binding pocket of *Ps*OMT2. A) Substrate binding site highlighted in cross-section of *Ps*OMT2*.* B) Substrate binding site highlighted in cross-section of *Ps*OMT2*.

**
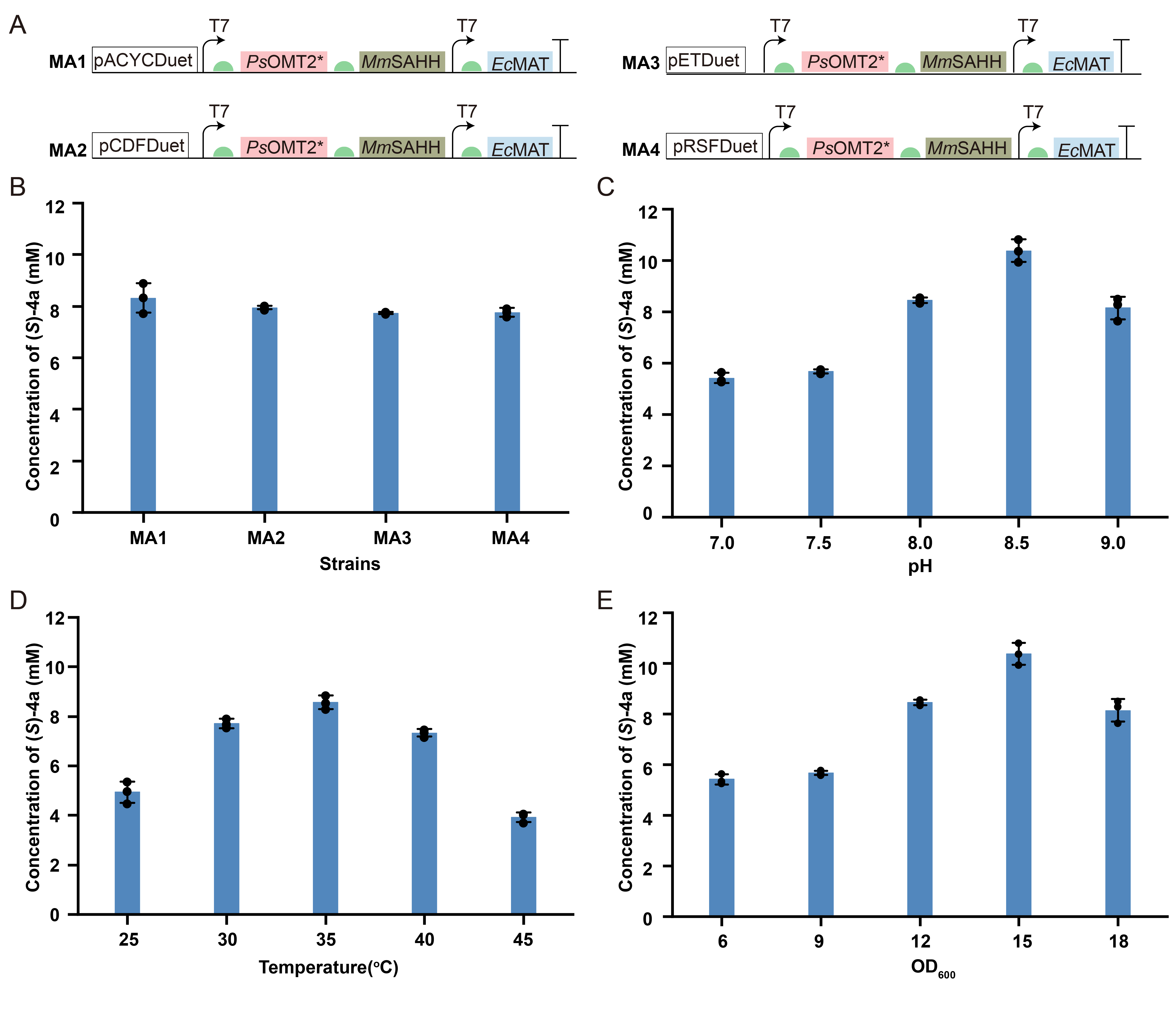
**

Figure S16. Optimization of the whole-cell reaction conditions of the 6OMT module. A) Construction of 4 engineered strains with different plasmids expressing *Ps*OMT2-I113R, *Mm*SAHH and *Ec*MAT. (B) Optimization of strains for the biosynthesis of (*S*)-**4a.** C) Optimization of temperature for the biosynthesis of (*S*)-**4a**. D) Optimization of pH for the biosynthesis of (*S*)-**4a**. (E) Optimization of strain concentration for the biosynthesis of (*S*)-**4a**. All data are presented as mean values of three independent experiments and the error bars indicate ±sd.

**
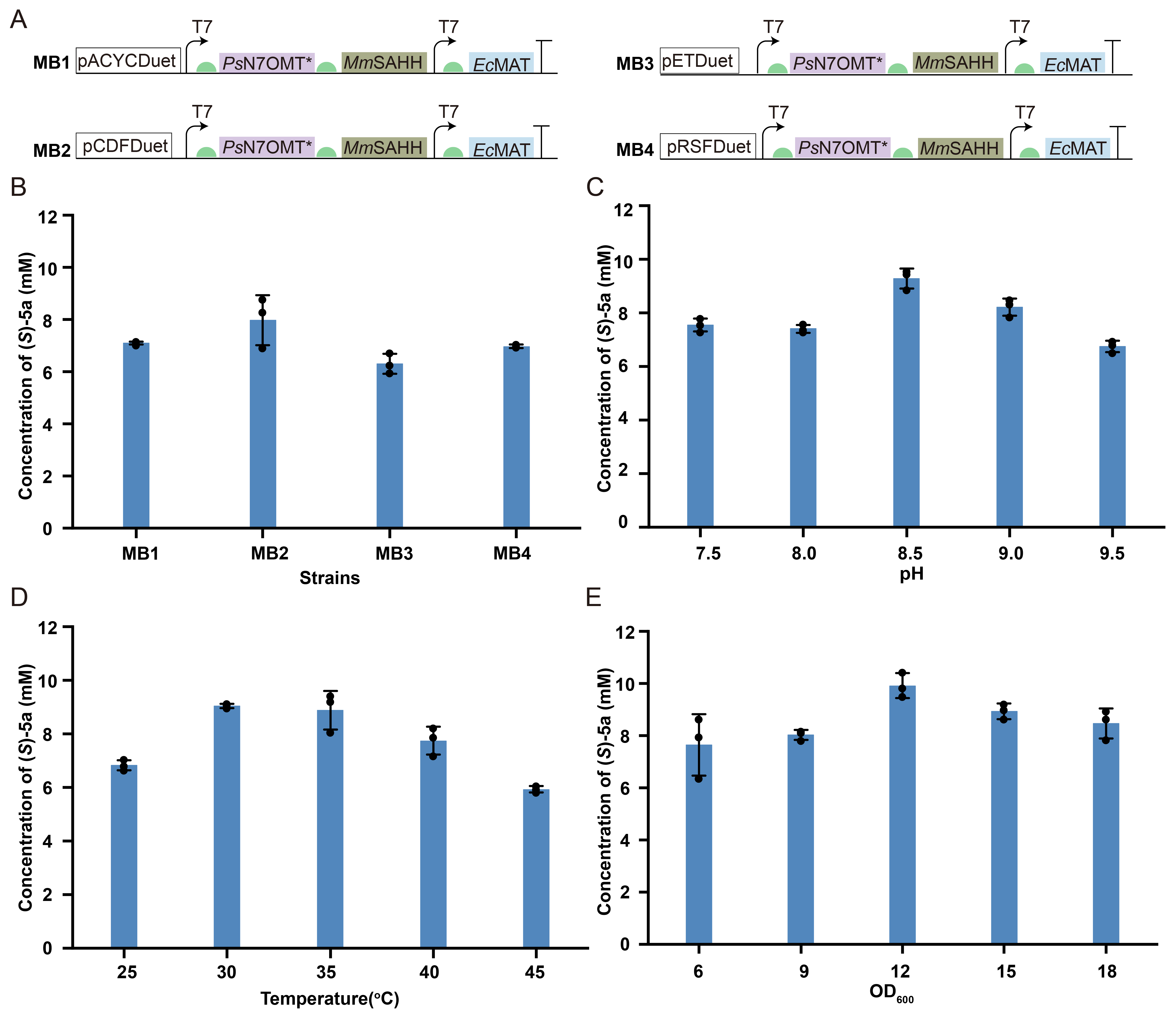
**

Figure S17. Optimization of the whole-cell reaction conditions of the 7OMT module. A) Construction of four engineered strains expressing *Ps*N7OMT2*, *Mm*SAHH and *Ec*MAT. B) Optimization of strains for the biosynthesis of (*S*)-**5a**. C) Optimization of temperature for the biosynthesis of (*S*)-**5a**. D) Optimization of pH for the biosynthesis of (*S*)-**5a**. (E) Optimization of strain concentration for the biosynthesis of (*S*)-**5a**. All data are presented as mean values of three independent experiments and the error bars indicate ±sd.


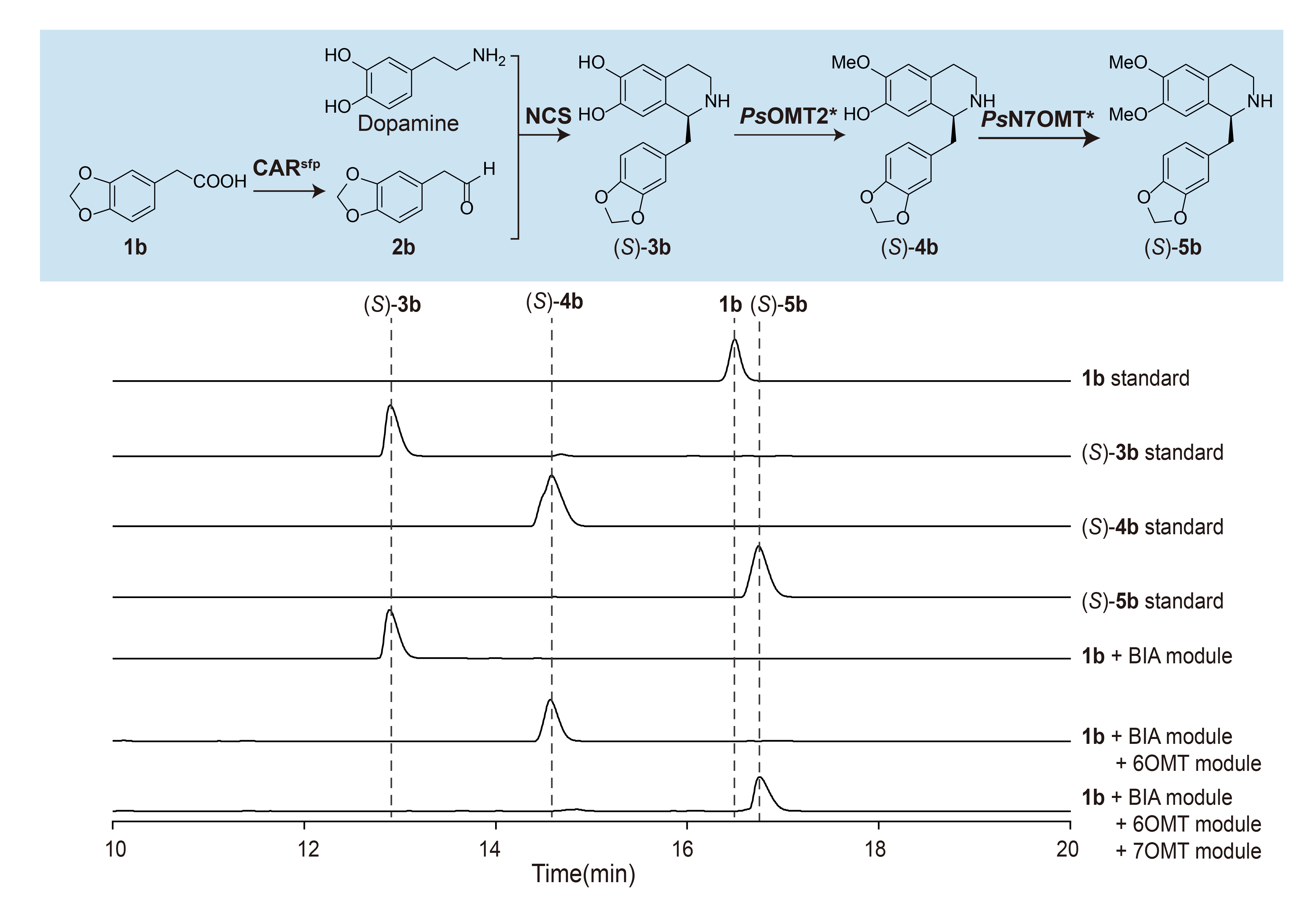


Figure S18. HPLC analysis of the biosynthesis of (*S*)-5b from 1b via whole-cell catalysis.


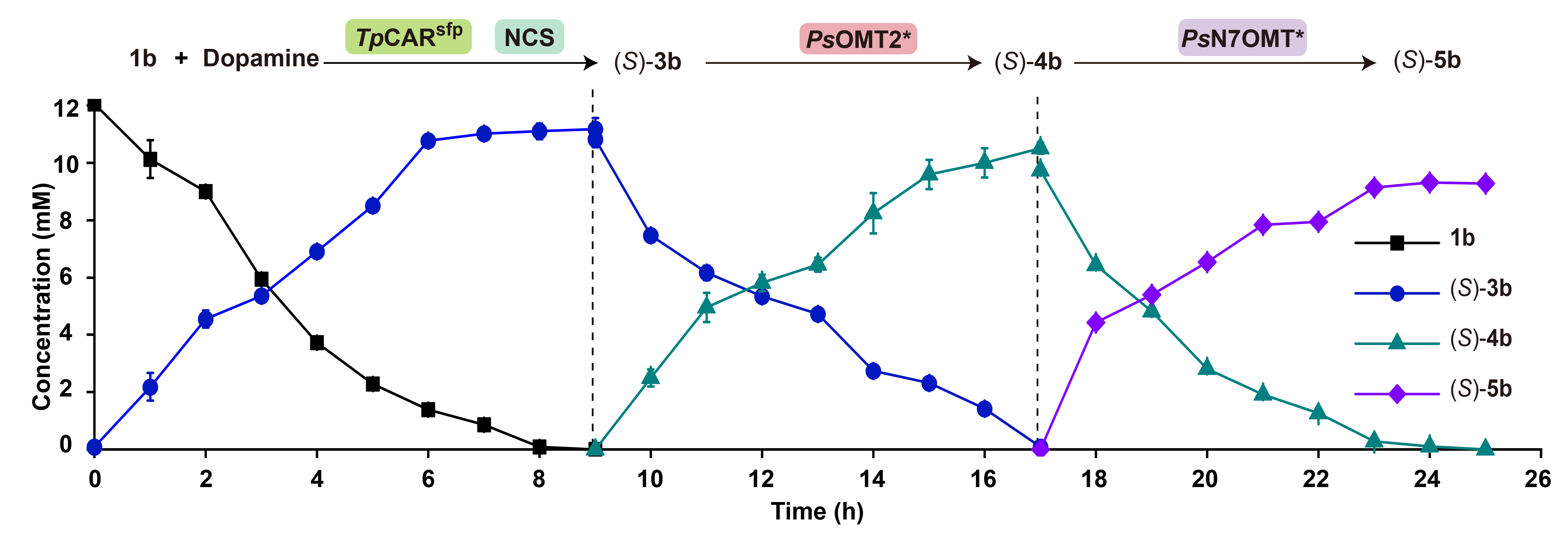


Figure S19. Time course of the biosynthesis of (*S*)-5b from 1b in 300 mL reaction system. All data are presented as mean values of three independent experiments and the error bars indicate ±sd.


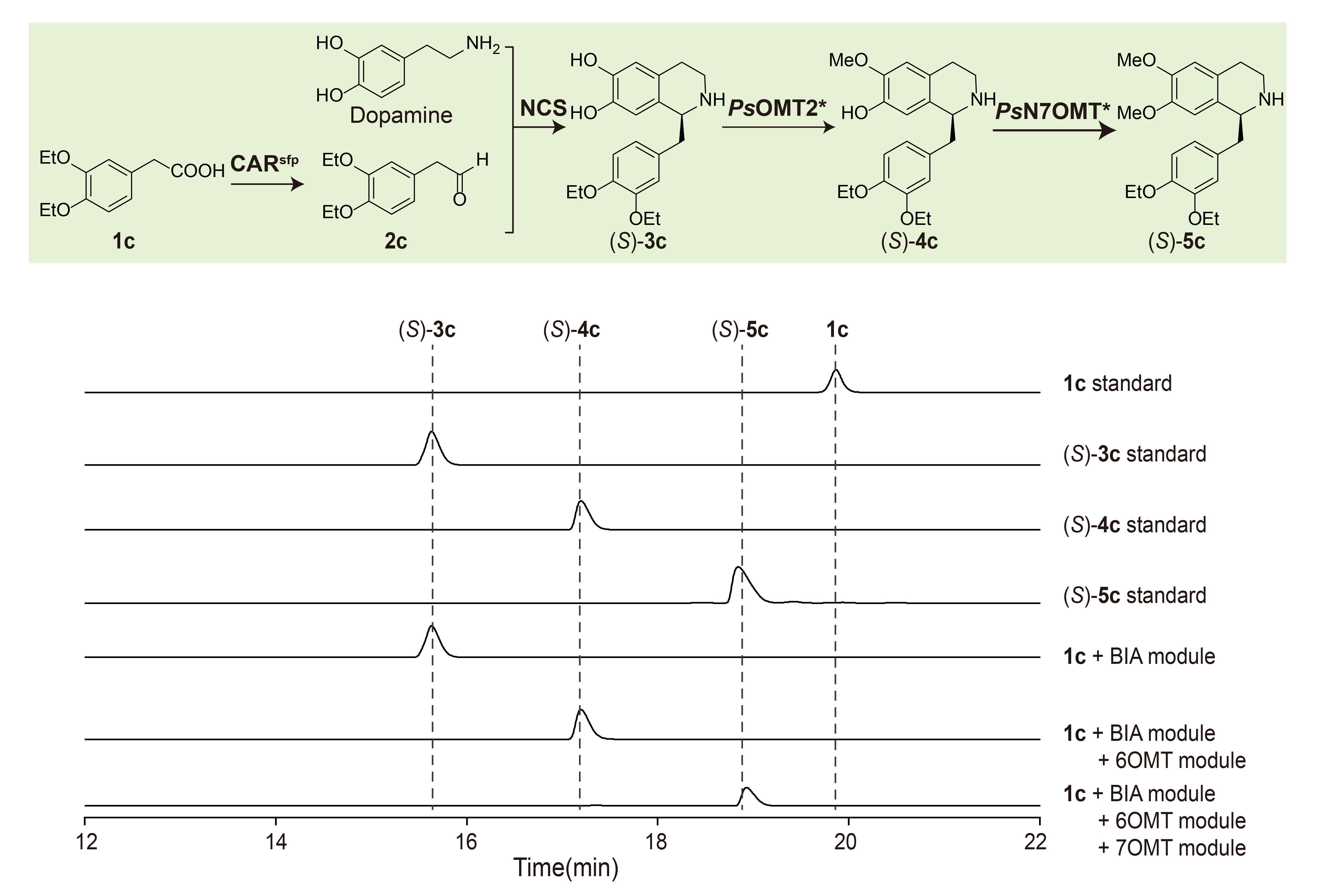


Figure S20. HPLC analysis of the biosynthesis of (*S*)-5c from 1c via whole-cell catalysis.


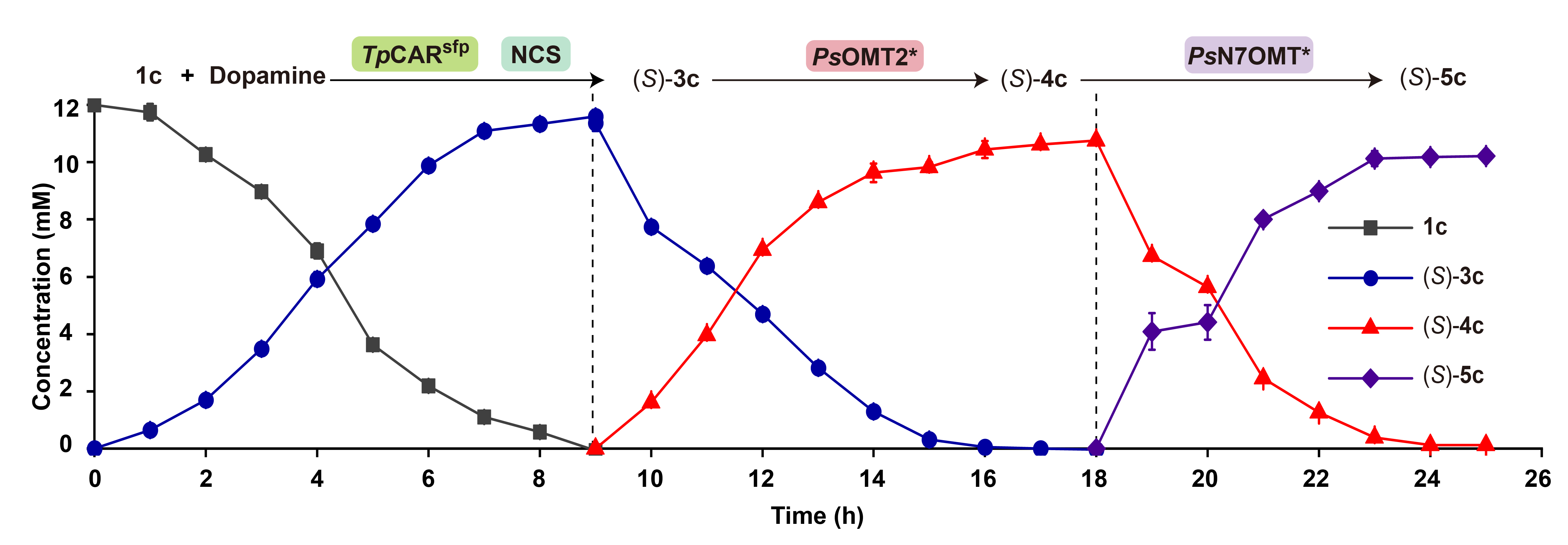


Figure S21. Time course of the biosynthesis of (*S*)-5c from 1c in 300 mL reaction system. All data are presented as mean values of three independent experiments and the error bars indicate ±sd.

**
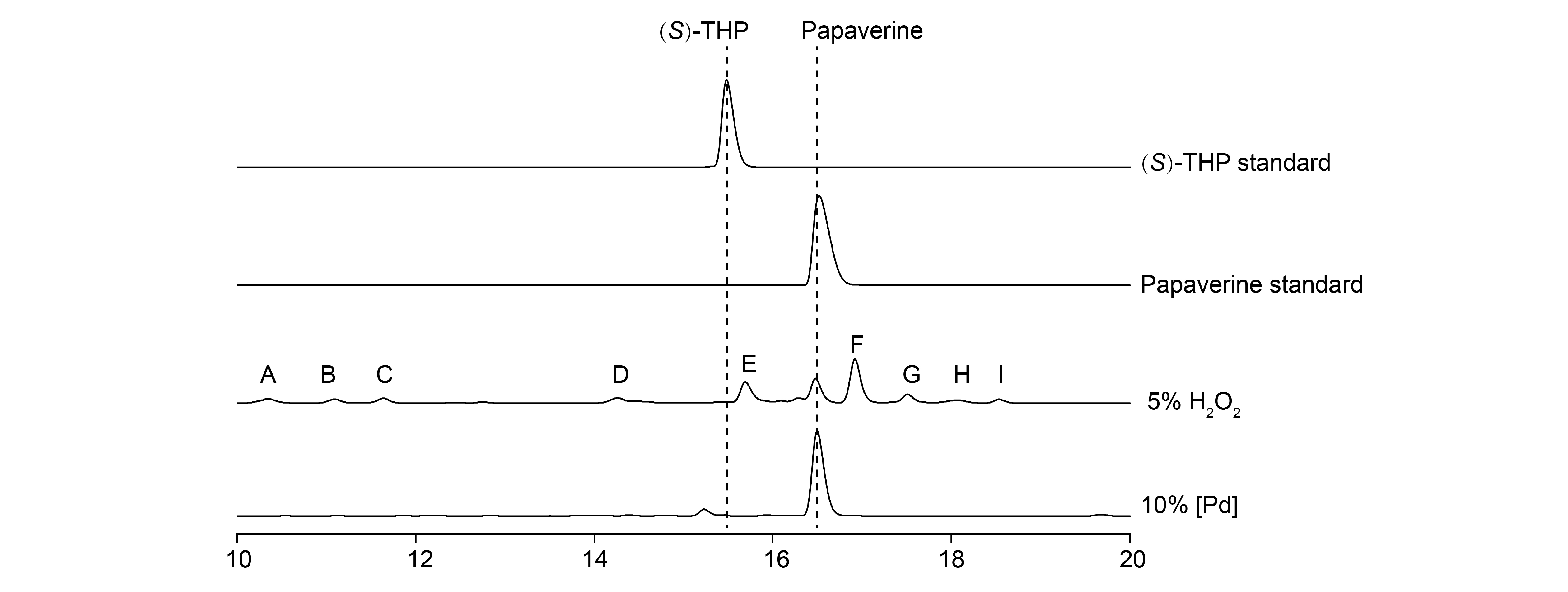
**

Figure S22. HPLC analysis of the oxidation reaction of (*S*)-THP to produce papaverine by hydrogen peroxide or Pd/C. Peaks A-I correspond to the byproducts detected by HPLC in hydrogen peroxide-mediated oxidation reaction.


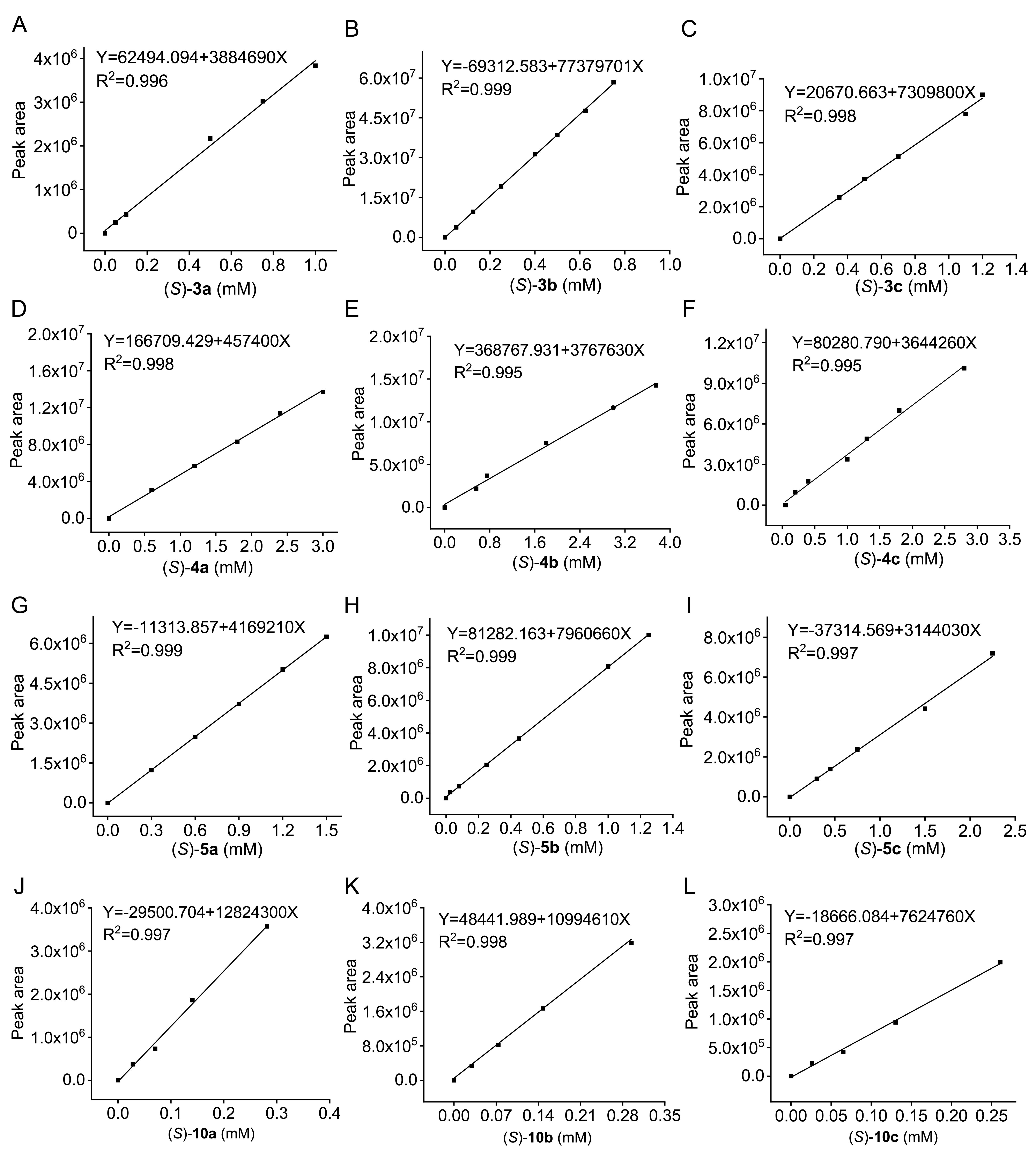


Figure S23. Standard curves of (*S*)-**3a-c,** (*S*)-**4a-c,** (*S*)-**5a-c,** and (*S*)-**10a-c**.

## Figure S24. ^1^H NMR and ^13^C NMR spectrum of (*S*)-3a.

## **Figure S25**. ^1^H NMR and ^13^C NMR spectrum of (*S*)-**3b**.

## Figure S26. ^1^H NMR and ^13^C NMR spectrum of (*S*)-5a.

## Figure S27. ^1^H NMR and ^13^C NMR spectrum of (*S*)-3b.

## **Figure S28.** ^1^H NMR and ^13^C NMR spectrum of (*S*)-**4b**

## **Figure S29.** ^1^H NMR and ^13^C NMR spectrum of (*S*)-**5b**.

## **Figure S30.** ^1^H NMR and ^13^C NMR spectrum of (*S*)-**3c.**

## **Figure S31.** ^1^H NMR and ^13^C NMR spectrum of (*S*)-**4c.**

## Figure S32. ^1^H NMR and ^13^C NMR spectrum of (*S*)-5c.

## **Figure S33.** ^1^H NMR and ^13^C NMR spectrum of **6a.**

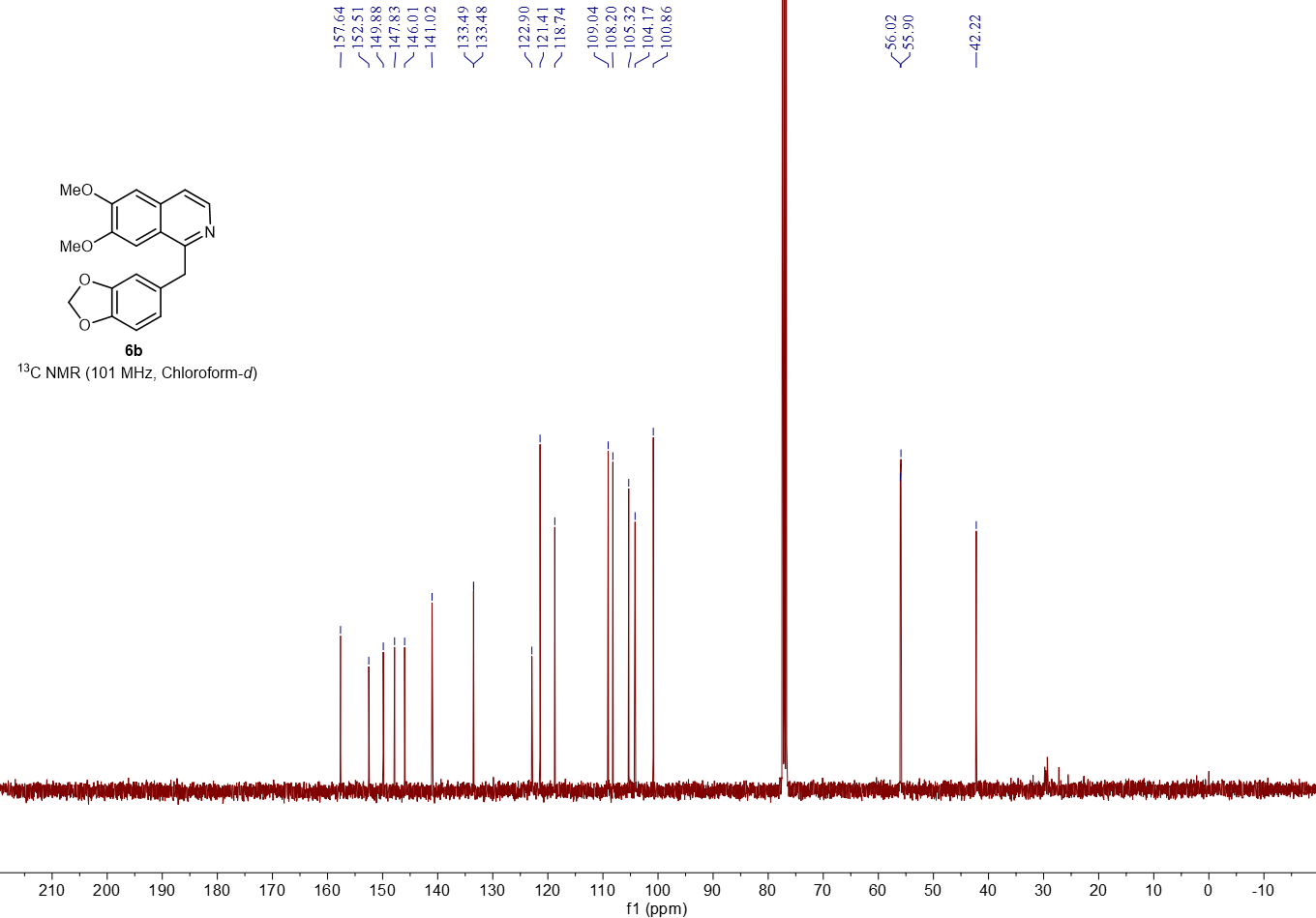


## **Figure S34.** ^1^H NMR and ^13^C NMR spectrum of **6b.**

## **Figure S35.** ^1^H NMR and ^13^C NMR spectrum of **6c.**

## **Figure S36.** ^1^H NMR and ^13^C NMR spectrum of (*S*)-**7a.**

## **Figure S37.** ^1^H NMR and ^13^C NMR spectrum of (*S*)-**7b.**

Figure S38. ^1^H NMR and ^13^C NMR spectrum of (*S*)-7c.

## **Figure S39.** ^1^H NMR and ^13^C NMR spectrum of (*S*)-**8a.**

## **Figure S40.** ^1^H NMR and ^13^C NMR spectrum of (*S*)-**8b.**

Figure S41. ^1^H NMR and ^13^C NMR spectrum of (*S*)-8c.

Figure S42. ^1^H NMR and ^13^C NMR spectrum of (*S*)-9a.

## **Figure S43.** ^1^H NMR and ^13^C NMR spectrum of (*S*)-**9b.**

## Figure S44. ^1^H NMR and ^13^C NMR spectrum of (*S*)-9c.

## Figure S45. ^1^H NMR and ^13^C NMR spectrum of (*S*)-10a.

## Figure S46. ^1^H NMR and ^13^C NMR spectrum of (*S*)-10b.

## **Figure S47.** ^1^H NMR and ^13^C NMR spectrum of (*S*)-**10c.**

## Reference

[1] G. Bringmann, R. Weirich, H. Reuscher, J. R. Jansen, L. Kinzinger, T. Ortmann, *Liebigs Annalen. der. Chemie.* **1993**, *1993*, 877-888.

[2] P. Peng, Z. Zheng, J. Yu, X. Gao, J. Yang, C. Zhao, F. Zhang, *Org. Process Res. Dev.* **2022**, *26*, 3106-3114.

[3] W.-S. Yu, Z.-N. Wu, Z.-F. Qiu, C.-J. Zhao, F.-L. Zhang, Z.-Z. Yang, *Russ. J. Org. Chem.* **2020**, *56*, 1295-1299.

[4] Y.-J. Wu, J.-H. Chen, M.-Y. Teng, X. Li, T.-Y. Jiang, F.-R. Huang, Q.-J. Yao, B.-F. Shi, *J. Am. Chem. Soc.* **2023**, *145*, 24499-24505.

[5] D. Badía, E. Anakabe, L. Carrillo, J. L. Vicario, M. Villegas, *Synthesis* **2004**, *2004*, 1093-1101.
